# Influence of current climate, historical climate stability and topography on species richness and endemism in Mesoamerican geophyte plants

**DOI:** 10.7717/peerj.3932

**Published:** 2017-10-20

**Authors:** Victoria Sosa, Israel Loera

**Affiliations:** Biología Evolutiva, Instituto de Ecologia AC, Xalapa, Veracruz, Mexico

**Keywords:** Trans-Mexican Volcanic Belt, Endemism, Diversity patterns, Ecological niche modeling, Spatial regression model

## Abstract

**Background:**

A number of biotic and abiotic factors have been proposed as drivers of geographic variation in species richness. As biotic elements, inter-specific interactions are the most widely recognized. Among abiotic factors, in particular for plants, climate and topographic variables as well as their historical variation have been correlated with species richness and endemism. In this study, we determine the extent to which the species richness and endemism of monocot geophyte species in Mesoamerica is predicted by current climate, historical climate stability and topography.

**Methods:**

Using approximately 2,650 occurrence points representing 507 geophyte taxa, species richness (SR) and weighted endemism (WE) were estimated at a geographic scale using grids of 0.5 × 0.5 decimal degrees resolution using Mexico as the geographic extent. SR and WE were also estimated using species distributions inferred from ecological niche modeling for species with at least five spatially unique occurrence points. Current climate, current to Last Glacial Maximum temperature, precipitation stability and topographic features were used as predictor variables on multiple spatial regression analyses (i.e., spatial autoregressive models, SAR) using the estimates of SR and WE as response variables. The standardized coefficients of the predictor variables that were significant in the regression models were utilized to understand the observed patterns of species richness and endemism.

**Results:**

Our estimates of SR and WE based on direct occurrence data and distribution modeling generally yielded similar results, though estimates based on ecological niche modeling indicated broader distribution areas for SR and WE than when species richness was directly estimated using georeferenced coordinates. The SR and WE of monocot geophytes were highest along the Trans-Mexican Volcanic Belt, in both cases with higher levels in the central area of this mountain chain. Richness and endemism were also elevated in the southern regions of the Sierra Madre Oriental and Occidental mountain ranges, and in the Tehuacán Valley. Some areas of the Sierra Madre del Sur and Sierra Madre Oriental had high levels of WE, though they are not the areas with the highest SR. The spatial regressions suggest that SR is mostly influenced by current climate, whereas endemism is mainly affected by topography and precipitation stability.

**Conclusions:**

Both methods (direct occurrence data and ecological niche modeling) used to estimate SR and WE in this study yielded similar results and detected a key area that should be considered in plant conservation strategies: the central region of the Trans-Mexican Volcanic Belt. Our results also corroborated that species richness is more closely correlated with current climate factors while endemism is related to differences in topography and to changes in precipitation levels compared to the LGM climatic conditions.

## Introduction

There is renewed interest in identifying centers of species richness and endemism because understanding the genesis of diversification makes it possible to identify the processes that maintain biodiversity and with this knowledge, strategies for long term conservation can be formulated (Brooks et al., 2006; Murray-Smith et al., 2009). Diversity richness patterns on Earth emerge from a combination of ecological and evolutionary processes such like speciation, migration and extinction (Ricklefs, 1987; Wiens & Donoghue, 2004).

Several of the drivers associated with patterns of species richness have been recently reviewed by Fine (2015) and summarized in five categories: (1) within-region species carrying capacity (depends on the amount of available resources on a given area, achieving an equilibrium of species diversity balanced by immigration, speciation or extinction); (2) species-energy combined with time-integrated area (integrated measures of area and energy over time should correlate with diversity or diversification rates at large taxonomic and temporal scales), in this hypothesis phylogenetic studies of large clades provide insights of the importance of area/energy/time in generating diversity gradients; (3) climate stability (areas that have been subjected to less severe changes in climate over time might accumulate more species); (4) biotic interactions (species from different trophic levels may interact more and influence speciation and extinction), more importantly predation, herbivory and mutualisms; and (5) temperature and evolutionary speed (temperature has a positive effect on the speed of physiological processes, and effective evolutionary speed should be higher in warmer areas).

Climate stability has been put forth as one of the most important drivers of global species richness patterns because climate has a direct impact on the probability of extinction (Fine, 2015). Different hypotheses have been proposed regarding the role that climate and topography play in patterns of species richness and endemism. For climate, the contemporary hypothesis suggests that the number of co-existing species varies according to current resource availability, while the historical climate hypothesis proposes that species differ in their ability to adapt to severe climate changes and that areas with a stable climate might favor long time persistence and speciation or by decreasing the opportunity for extinction (Dynesius & Jansson, 2000; Wiens & Donoghue, 2004; Jablonski, Kaustuv & Valentine, 2006). For historical climate in particular, the orbitally forced species’ range dynamics (ORD) hypothesis suggests that Milankovitch climate oscillations, especially the Quaternary glacial-interglacial shifts, limited the distribution of many species to zones of long term spatiotemporal climate stability as consequence of these climate oscillations (Dynesius & Jansson, 2000; Svenning et al., 2015). The influence of topographic features has also been considered a potential driver for species richness and endemism patterns. Greater topographic complexity, along with a heterogeneous climate, allows species with diverse evolutionary strategies to coexist at small geographic scales. Moreover, under scenarios of climate change, topographic variation might have an effect facilitating short-distance shifts in species distributions, making it possible for taxa with a low dispersal capability to survive even with low migration rates (Loarie et al., 2009). Nevertheless, the influence of historical climate, current climate and topography are not mutually exclusive, and both species richness and endemism are likely to be the consequence of their interaction (Francis & Currie, 2003). The impact of historical climate is probably greater on endemism, since endemic species have restricted distributions, and in many cases persisted in small refugia where habitat and climate were stable for long periods of time (Araújo et al., 2008; Gavin et al., 2014). This impact on endemism has been corroborated in different biogeographic areas and for various taxa (e.g., Baselga, 2008; Ohlemüller et al., 2008; Huang et al., 2012; Sandel et al., 2011; Abellán & Svenning, 2014; Rakotoarinivo et al., 2013; Feng et al., 2015; Ma, Sandel & Svenning, 2016; Noss et al., 2015; Molina-Venegas et al., 2016; Harrison & Noss, 2017), though its effect in tropical and inter-tropical regions like Mexico is not yet fully understood.

In this study we focus on current and historical climate variation and topographic features to understand the potential drivers of variation on species richness and endemism for a functional group of plants, the geophytes; herbaceous plants that lose their aerial organs annually and possess underground organs such like bulbs, corms, tubers or rhizomes (Kamenetsky, 2013). These organs act as nutrient reserves and allow plants to survive periods of severe environmental conditions, such as prolonged dry seasons, high and low temperatures, and high solar radiation, and to grow rapidly after the rainy season (Parsons, 2000). Organ size is probably related to microhabitats, to local drainage conditions, and thus represents habitat specialization (Procheş, Cowling & Du Preez, 2005; Procheş et al., 2006). Geophytism has evolved numerous times in different parts or the world and is common in areas with a mediterranean climate and probably geophytes have been successful in these biomes because they have a survival advantage in ecosystems with a high degree of seasonality in precipitation (Parsons, 2000; Procheş, Cowling & Du Preez, 2005; Procheş et al., 2006). Moreover, in several mountains, geophyte taxa are also found at high elevations (above 2,000 m) that do not have a mediterranean climate, with wet winters, mild summers but with precipitation seasonality. Furthermore numerous geophyte species only flower or germinate after burning and many of them lose their leaves during the flowering period (Procheş, Cowling & Du Preez, 2005; Procheş et al., 2006).

**Figure 1:**
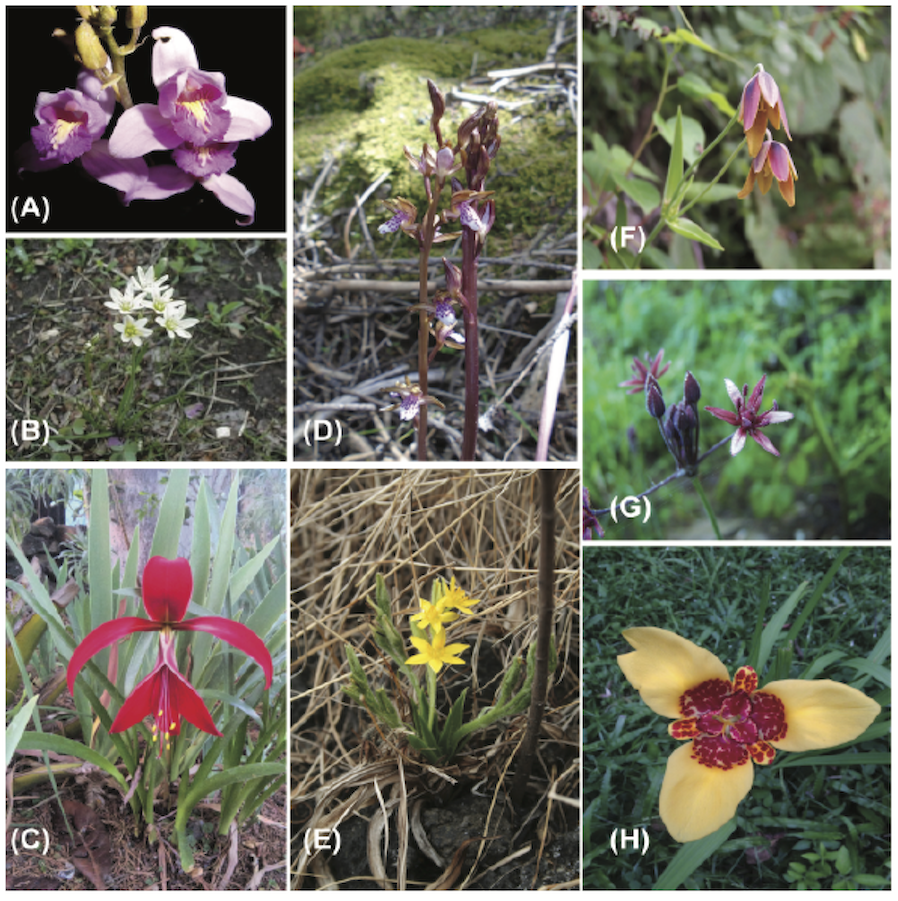
Representative monocot species of geophytes recorded in Mexico. (A) *Bletia* sp. (in the *reflexa* complex), (B) *Nothoscordum bivalve*, (C) *Sprekelia formossisima*, (D) *Corallorhiza maculata*, (E) *Hypoxis mexicana*, (F) *Calochortus barbatus*, (G) *Allium glandulosum*, (H) *Tigridia pavonia*. (Photo credits: (A, C, F, G) Eduardo Ruiz Sanchez; (B) Etelvina Gandara; (D) Rene Palestina; (E) Wynn Anderson; (H) Regina Cuevas Chavez).

**Figure 2:**
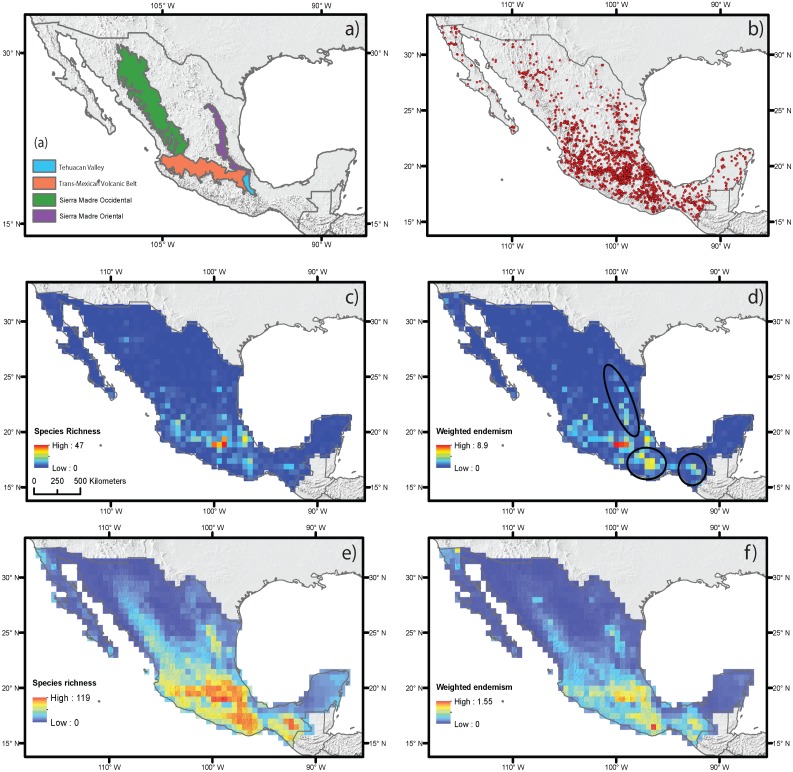
Estimated species richness and weighted endemism for the monocot geophyte species distributed in Mexico based on ecological niche modeling using current climate variables. (A) Main mountain chains in Mexico. (B) Occurrence records. (C) Species richness measured by direct records. (D) Weighted endemism measured by direct records. (E) Species richness estimated by ecological niche modeling. (F) Weighted endemism estimated by ecological niche modeling.

As indicated above, to understand the effect of current and historical climate on species richness and endemism in the Mesoamerican plants we selected the monocot geophytes of Mexico (see examples of species in Fig. 1). The country is megadiverse and has approximately 25,500 vascular plant species (CONABIO, 2016). Of these, around 4,500 are monocots, half of which are endemic (Espejo-Serna, 2012). This region is characterized by a complex geological and climate history with 60 different types of climate, the major climates in Mexico are tropical wet, tropical wet-and-dry, semi-arid, arid, temperate with dry winters, humid subtropical and mediterranean (Kottek et al., 2006) and 51 terrestrial eco-regions that correspond mostly to the mountain chains (Olson et al., 2001). The country is crossed by several large mountain ranges: the Sierra Madre Oriental, the Sierra Madre Occidental, the Trans-Mexican Volcanic Belt and the Sierra Madre del Sur (Fig. 2A). The origin of these mountain systems is complex, and is the result of different episodes of mountain uplift, mainly in the Early and Mid-Cenozoic with scarce orogenic activity in the Pleistocene when climate changes were more important factors driving changes in vegetation composition (Ferrusquía-Villafranca, 1993; Mastretta-Yanez et al., 2015). The Trans-Mexican Volcanic Belt originated later, in the Mid-Miocene, with some episodes of volcanism occurring more recently, in the Late Pleistocene (Ferrari et al., 2012). Climate change during the Pleistocene in these mountains is associated with the dynamic history of vegetation distribution and community composition which together with the topographic complexity of some of these areas may have contributed to the survival of species by promoting habitat heterogeneity (Toledo, 1982; Mastretta-Yanez et al., 2015). Paleorecords from the Miocene and Pliocene in several areas of Mexico identified complex plant communities reflecting a warmer, more humid climate compared with that of the Quaternary (Graham, 1999). Furthermore, there were important climate changes over the Late Pleistocene and Holocene in Central Mexico, the Yucatan and Northern Mexico, areas for which we have reliable paleoclimate records that reveal notable climate changes for that period (Metcalfe et al., 2000).

Endemism in the Mexican flora has been found to be associated with dry current climates and the endemism of shrubby and perennial herbaceous species in particular has been associated with more humid temperate climates (Rzedowski, 1993). By contrast, the most elevated richness has been associated with tropical wet climates typical of evergreen forests of the south (Rzedowski, 1993). Furthermore, a number of plant lineages are endemic to Mexico and endemism hotspots for these lineages have been identified as areas of dry climate with xeric vegetation comprising several monocot groups with geophyte life forms (Sosa & De-Nova, 2012; Gándara, Specht & Sosa, 2014). On the contrary, endemic Mexican avifauna is centered in more humid habitats in the Sierra Madre Occidental and in the Sierra Madre del Sur (Villalobos et al., 2013), endemism in amphibians in the tropical rainforests of southern Mexico (Aguilar-López et al., 2016), and in small endemic mammals, habitats characterized by climatic and topographic variability in northern Baja California and in the mountains of central and southern Mexico (Arita et al., 1997).

Our previous study identified approximately 500 Mexican geophyte monocot species, the majority belong to four plant families: Amaryllidaceae, Asparagaceae, Iridaceae and Orchidaceae, with ten small genera endemic to Mexico (Cuéllar-Martínez & Sosa, 2016). Moreover, these Mesoamerican geophytes are found in climates with temperature and precipitation seasonality and many have restricted distributions. Nonetheless it is essential to identify the role that historical climate played on species richness and endemism of this functional group of plants that is able to thrive in seasonal climates and harsh microhabitats.

Our goal was to determine the extent to which species richness and endemism in the monocot geophyte taxa of Mexico are associated with current and historical climate stability, and topography. Our hypothesis is that species richness is best predicted by current climate and endemism is best explained by climate stability from the time of the Last Glacial Maximum and by topography.

## Methods

### Species richness and weighted endemism based on presence data

Occurrence data for the Mexican geophyte taxa was based on Cuéllar-Martínez & Sosa (2016), comprising 2,629 occurrence points for 507 species (see Table S1 for complete species list and number of occurrences per species; see also Fig. 1 for examples). The monocot geophyte checklist was based on Espejo-Serna (2012), and complemented with recently described species. Georeferences were obtained by: (1) specimens from Mexican herbaria such as: ENCB, IBUG, IEB, MEXU, XAL (acronyms based on Thiers, 2017); (2) biodiversity databases such as the Global Biodiversity Information Facility, GBIF (http://www.gbif.org/), and the Mexican Biodiversity Database, REMIB (http://www.conabio.gob.mx/remib/); (3) complemented by monographs and floras. A grid with a pixel size of 0.5 decimal degrees was overlaid on a map of Mexico (796 pixels in total to cover the complete extent of Mexico). Species richness (SR) and weighted endemism (WE) were estimated directly using the occurrence points considering each species as absent or present on each pixel. SR was then calculated as the total number of species present on each pixel. For endemism we used the measure of weighted endemism proposed by Linder (2001a) and Linder (2001b), in which the area of interest is divided in grid squares labeled by the degrees of latitude and longitude and the occurrence records are mapped on the grids. The index for weighted endemism is divided by the grid-diversity; each species in every grid is weighted by the inverse of its distribution range in a way that a species found in a single grid is scored as 1. Grids with a small number of species in total, but proportionally with many range-restricted species, display more effectively than species-rich grids with proportionally range-restricted species, giving a corrected index of endemism (Linder, 2001a; Linder, 2001b). Weighted endemism is the sum of the reciprocal of the total number of cells in which each species is found. Thus, WE emphasizes areas that have a high proportion of species with restricted ranges (Crisp et al., 2001). SR and WE were calculated in ArcMap 10.3 using the toolbox SDMtoolbox v1.1c (Brown, 2014). We also estimated SR and WE using species distribution modeling (SDM) for the species with more than five spatially unique occurrence data (107 species, see Table S2) as explained below. We decided to use this reduced dataset of species because SDM is known to have usually low performance when modeling species with very restricted distributions especially when using datasets with less than five points.

### Species richness and weighted endemism based on species distribution models

We also used ecological niche modeling to predict both species richness (SR) and weighted endemism (WE) using the estimated distributions based on niche models. Ecological niche models were estimated for species that had at least five spatially unique occurrence points at a resolution of 10 min. Model performance was evaluated using the area under the curve (AUC) and the true skill statistic (TSS) for all estimated niche models and we only used the models that showed good model performance (see Supplementary S2 for AUC and TSS statistics). The niche models were estimated with MaxEnt 3.3 (Phillips & Dudík, 2008) using 10 replications and cross validation. Default values were used for other parameters. The environmental inputs for generating these niche models were based on WorldClim v. 1.4 (Hijmans et al., 2005) bioclimate variables at 10 min resolution for average conditions of 1960–1990 (available at: http://www.worldclim.org/version1). These variables were first selected based on a Pearson correlation analysis (selected variables with *r* values ≤ 0.70) for the climate data extracted from all spatially unique points. The variables used for niche modeling were: Bio1 = Annual mean temperature, Bio3 = Isothermality (Mean diurnal range/Temperature annual range) (*100), Bio4 = Temperature seasonality, Bio12 = Annual precipitation, Bio15 = Precipitation seasonality, and Bio16 = Precipitation of coldest quarter. Continuous suitability maps were converted to binary using a maximum training plus specificity threshold for species presence for each replicate (Liu, White & Newell, 2013). The latter were then summarized on a binary (presence/absence) map for each species modeled, assuming the presence of species in those pixels that were predicted in all replicates and using them to estimate SR and WE at a 0.5 decimal degrees resolution in SDMtoolbox v1.1c (Brown, 2014) with ArcMap 10.3. Additionally, we also calculated species richness and endemism independently for the four most diverse families of geophyte taxa in this study (Amaryllidaceae, Asparagaceae, Iridaceae and Orchidaceae).

### Current climate, historical climate stability and topography

Different predictor variables that represent variations in current climate, climate stability from the LGM, and topography were calculated for our study area. Current climate data was taken from the WorldClim database (Hijmans et al., 2005) using Mexico as the geographic extent. For the historical variables of climate stability we calculated the anomalies between averages for the bioclimate variables from the Last Glacial Maximum (LGM) and current climate scenarios. The climate model CCSM was utilized for LGM and it is available at WorldClim (http://www.worldclim.org/version1), we chose this model based on previous research analyzing climate for LGM in Mexico as well as tests comparing performance of the main models for this period (Ramírez-Barahona & Eguiarte, 2013; Varela, Lima-Riberio & Terribile, 2015). The anomalies between current and past climate were calculated as the absolute value of the difference between present and past variables (e.g., the absolute value of the difference between BIO1 at present and average BIO1 during the LGM). Thus, these historical variables reflect how similar the present climate of each pixel is relative to the LGM average, with higher values indicating larger anomalies. For topography we used elevation (EL) and the topographic roughness index (TRI). TRI shows the variance on elevation of the group of adjacent pixels at a given area, thus reflecting areas that have elevated altitude heterogeneity.

### Simultaneous autoregressive models

The estimates based on climate and topographic data were used as predictor variables using spatial autoregressive models (SAR) implemented in SAM (Rangel, Diniz-Filho & Bini, 2010). We decided to use spatial regression models because they are suitable for data with spatial autocorrelation (i.e., adjacent pixels are more similar among themselves than expected by chance) as expected for environmental and species distribution data (Kissling & Carl, 2008). Prior to running these analyses all predictor and response variables were log transformed: log 10 (*x* + 1). The spatial regressions were carried out taking all predictor variables simultaneously and independently using SR and WE based on both approaches (i.e., direct occurrence points and distribution modeling) as response variables. We tested alpha values from one to five units and by visualizing Moran’s I correlograms for all distance units residual errors of spatial correlations were removed. The optimal value of alpha was determined to be five for the SR and WE estimated with SDM and an alpha value of two was used for the dataset based on direct occurrence data. Only the predictor variables that were significant in the regression analyses were used as a measure of their association with the response variable. We utilized the estimated standardized coefficients of these variables as a measure of the extent of their association to explain variation in SR and WE and to discuss the differences in their contribution to the alternative predictor variables according to our research hypotheses.

## Results

### Geographic patterns of species richness and endemism

The estimation of SR and WE using direct occurrence data and SDM yielded similar results (Figs. 2C–2F), but distribution modeling had more areas with the highest richness and endemism. The estimated SR and WE of Mexican monocot geophytes mostly coincided in the same areas, though the extent of the area varied. Richness was highest in the mountainous areas of central Mexico, on the Trans-Mexican Volcanic Belt, with lower values in the southern areas of the Sierra Madre Occidental and Sierra Madre Oriental mountain ranges and in the Balsas Basin and the Tehuacán Valley (Figs. 2C and 2E). The highest degree of endemism was restricted to the central area of the Trans-Mexican Volcanic Belt and to particular areas in the Tehuacán Valley and the Balsas Basin (Figs. 2D and 2F). Although SR and WE had similar geographic patterns, some important differences were observed in different mountainous regions of Mexico such as the Sierra Madre del Sur, Chiapas and Sierra Madre Oriental that had some of the highest levels of endemism but did not coincide with the highest species richness particularly for the direct estimates based on occurrence data (dark circles Fig. 2B).

Species richness and endemism based on current climate variables for Amaryllidaceae, Asparagaceae, Iridaceae, and Orchidaceae are shown in Fig. S1. Species richness for Amaryllidaceae and Iridaceae had similar geographic patterns with the highest richness in the eastern section of the Trans-Mexican Volcanic Belt decreasing to the western section. For Asparagaceae and Orchidaceae, species richness was greatest in the central area of the Trans-Mexican Volcanic Belt, decreasing in the western section towards the southern area of the Sierra Madre Occidental. The weighted endemism analysis revealed differences in the area occupied by groups, with Asparagaceae and Orchidaceae more restricted. The geographic patterns of weighted endemism were very similar to those of species richness (Fig. S1).

**Table 1:** Simultaneous autoregressive models between estimated species richness and weighted endemism for monocot geophytes with current and historical climate variables.

Abiotic drivers	Variable	Estimates based on occurrence data				Estimates based on distribution modelling			
		Species richness		Weighted endemism		Species richness		Weighted endemism	
		Std coeff.	*P* value	Std coeff.	*P* value	Std coeff.	*P* value	Std coeff.	*P* value
Current climate	Mean annual temperature (MAT)	−0.129	0.231	0.099	0.304	0.011	0.904	−0.038	0.65
	Mean annual precipitation (MAP)	**0.313**	<**.001**	0.154	0.054	**0.37**	<**.001**	**0.218**	**0.001**
Historical climate	LGM Stability (SMAT)	0.034	0.65	0.099	0.189	−**0.236**	<**.001**	−**0.13**	**0.049**
	LGM Stability (SMAP)	**−0.156**	**0.019**	−**0.181**	**0.007**	**0.164**	**0.005**	0.07	0.195
Topography	Topographic ruggedness index (TRI)	**0.144**	**0.04**	0.108	0.147	**0.281**	<**.001**	**0.257**	<**.001**
	Elevation (EL)	−0.02	0.836	**0.175**	**0.041**	0.059	0.36	0.049	0.419
		(Predictor) *R*^2^ = 0.27		(Predictor) *R*^2^ = 0.18		(Predictor) *R*^2^ = 0.64		(Predictor) *R*^2^ = 0.37	
		(Predictor + space) *R*^2^ = 0.35		(Predictor + space) *R*^2^ = 0.25		(Predictor + space) *R*^2^ = 0.87		(Predictor + space) *R*^2^ = 0.64	

**Notes.**

MAT Annual mean temperature MA Annual mean precipitation SMAT stability on mean annual temperature SMAP stability on mean annual precipitation TRI topographic roughness index EL elevation

### Spatial regression analyses

Spatial regression analyses for the estimates based on direct occurrence data showed that the predictor variables explained SR (*R*^2^ = 0.35) (Table 1). Variables with the highest standardized coefficients in the regression using SR included the three types of abiotic predictors: current climate, historical climate and topography. These variables were annual mean temperature (MAP), topographic roughness index (TRI) and stability of mean annual precipitation (SMAP) (Table 1). Among them, the variable with the highest coefficient was MAP, which was positively correlated with species richness, suggesting that the areas with the highest current annual precipitation (green shading in Fig. 2D) harbor more geophyte species. SMAP and TRI showed a weaker but significant association, with negative and positive associations with SR, respectively. This suggests that areas that have changed more in their annual precipitation regimes from the LGM to the present (i.e., less stable precipitation, orange shading in Fig. 2B) have higher species richness. Spatial regressions using WE as the response variable showed that the predictor variables were able to explain SR (*R*^2^ = 0.25). Significant predictors with the highest standardized coefficients were historical and topographic variables (SMAP and ALT). Both predictors were positively associated with WE, suggesting that areas with the highest geophyte endemism are characterized by significant changes in the level of precipitation since the LGM and high elevations (represented in Figs. 2B and 2E by red and dark shading, respectively).

**Figure 3:**
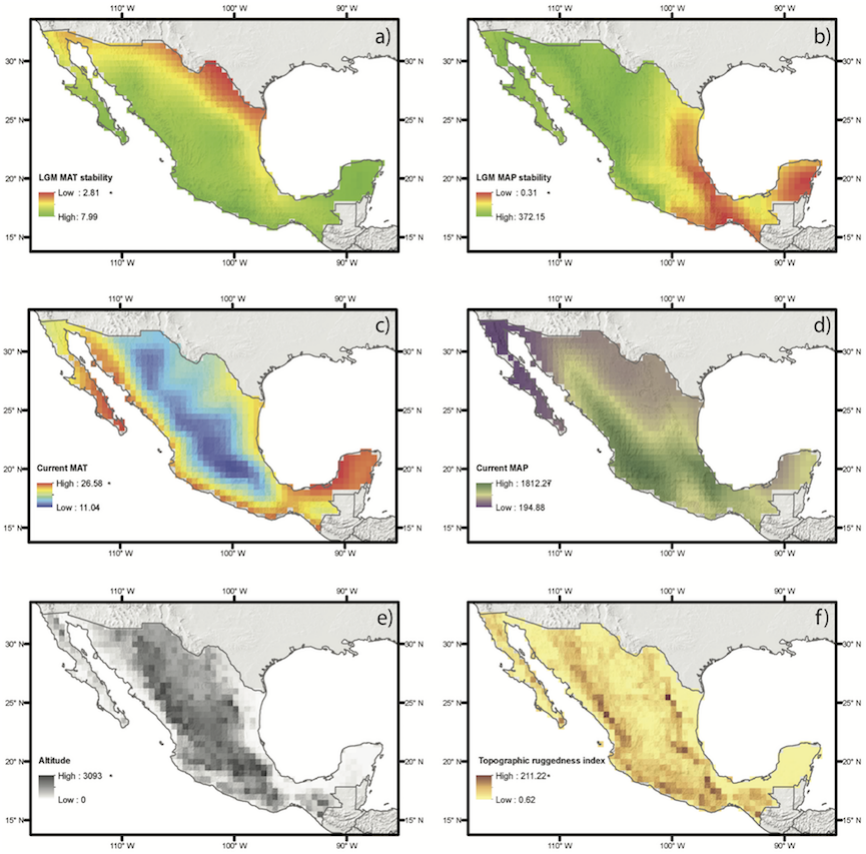
Abiotic drivers of variation on species richness and weighted endemism tested in this study. Each map shows a color ramp that represents the values of each continuous variable for each group, which includes historical climate (A, B), current climate (C, D) and elevation and topography (E, F).

Spatial autoregressive analyses based on estimates of SR and WE using distribution modeling for the species with at least ten occurrence data points (Table 1, Fig. 3) yielded results similar to those of the regressions based on direct occurrence data, though with stronger associations (*R*^2^ = 0.87 and *R*^2^ = 0.64 for SR and WE, respectively). SR and WE were predicted by the three abiotic factors: current climate, historical climate and topography (Fig. 3).

## Discussion

### Estimation of species richness and weighted endemism based on occurrence data and ecological niche modeling

The availability of online georeferenced data for taxonomic groups and high-resolution environmental information has facilitated research on species richness-environment relationships and has also allowed for an understanding of the historical and current drivers of variation in species richness (Araújo & Guisan, 2006; Araújo et al., 2008; Loarie et al., 2009). However, even comprehensive distribution databases are expected to underestimate species richness. Particularly for plants, inventory is incomplete for many groups and many regions or species have very restricted distributions with a few records and this might affect the reliability of environment-spatial prediction of species richness (e.g., Yan, Yang & Tang, 2013; Meyer, Weigelt & Kreft, 2016). Moreover, it has been demonstrated that larger sample sizes are needed to correctly map the distribution ranges of species (Feeley & Silman, 2011). Many Mesoamerican monocot geophytes have limited distributions and although they have been collected throughout their range they do not reach an elevated number of records, and some of them are known exclusively from the type collection in a single locality. Ecological niche modeling has been proposed as an alternative for improving the estimation of species distribution when sampling might be incomplete for some regions or scarce for certain groups of organisms and we followed this approach (Graham & Hijmans, 2006). Here we found that estimates of SR and WE, whether by occurrence data or by ecological niche models, yielded similar results, although for WE we only included species with wider distributions and those that had good model performance compared with the occurrence dataset that included all species georeferences (Van Proosdij et al., 2016). These results suggest that there is a strong relationship between climate and species richness for geophyte taxa, for both the species that have restricted distributions (e.g., *Petronymphe decora, Milla valliflora*) and those that have wider distributions (e.g., *Nothoscordum bivalve, Sprekelia formossisima*, see Fig. 1). Thus, factors limiting the extent of the distribution of some species might be related to dispersal ability, biotic interactions or time since speciation.

### Patterns of species richness and weighted endemism

According to our results, the richness and endemism of monocot geophytes are highest in mountain chains such as the Trans-Mexican Volcanic Belt and the southern parts of two mountain ranges: the Sierra Madre Oriental and the Sierra Madre Occidental (see Fig. 2). These patterns were identical when the four most diverse families are analyzed (Orchidaceae: 181 species, Asparagaceae: 114 species, Amaryllidaceae: 106 species and Iridaceae: 92 species; see Fig. S1).

Little is known about climate change in the mountains of Mexico, however it has been reported that during the glacial periods of the Pleistocene temperatures decreased and precipitation regimes changed, as did seasonality along the Trans-Mexican Volcanic Belt (Metcalfe, 2006). In the late Pleistocene the western section of this ridge was wetter than it is at present, while the eastern part was drier than at present (Bradbury, 1997; see also Fig. 3B). At the end of the Pleistocene the Trans-Mexican Volcanic Belt was very dry in many areas and wetter conditions were identified at the beginning of the Holocene (Metcalfe et al., 2000). Seasonal climates during these periods likely favored the establishment of these taxa along this range. Seasonal climates have been identified as suitable for monocots with this life form here and in a previous study (Cuéllar-Martínez & Sosa, 2016). Moreover, the same areas of the Trans-Mexican Volcanic Belt are also associated with a high degree of richness and endemism for other plant groups, such as the perennial species of *Cosmos* (Asteraceae, Vargas-Amado et al., 2013), tribe Tigridieae (Iridaceae), which includes most of the geophytes studied here (Munguía-Lino et al., 2015), and fern species in the order Polypodiales (Sanginés-Franco et al., 2015).

It has been indicated that rise of temperature is one of the most important drivers of shifts of species ranges in a number of plant life forms such like trees, epiphytes and understorey plants and that species are limited in their capacity to track climate warming (Hsu et al., 2012; Dullinger et al., 2015; Sittaro et al., 2017). Furthermore climate velocity has been calculated in some areas such like North America finding that vectors for climate variables display a complex mosaic of patterns that vary in space and time and are dependent on the spatial resolution of input data (Dobrowski et al., 2013). On the other hand, it has been pointed out that plant movements are complex and that are not realistically represented in models actually utilized to predict future vegetation changes and that many plant species will probably lag behind broad-scale patterns of climate change (Corlett & Westcott, 2013). The grid utilized by us was of 50 × 50 km and based on the historical climate variables and on the predictive ecological niche models, species movements could be estimated. However in view of the complexity of plant movement, the migration lag and the complex pattern of climate change in regions we decided not to estimate this movement. Nevertheless more accurate models in the future might assess the movement of geophytes in Mesoamerica.

### Abiotic drivers of variation in species richness and endemism

Our results suggest that although species richness is mostly influenced by the current climate, there is a strong historical component that has shaped the patterns of geophyte species diversity and affected patterns of endemism. Our hypothesis was that species richness would be better predicted by the variability in current climate and that endemism would be better predicted by climate stability from the time of the Last Glacial Maximum to the present and by topographic features. However, spatial regressions showed that historical climate variables also played an important role in shaping species richness, particularly on mean annual precipitation stability. Mean annual precipitation stability was negatively associated to SR suggesting that the areas that currently have higher precipitation respect to the LGM prediction have more species. These results coincide with those reported for other plants and regions. For example, palm species richness in Madagascar was strongly influenced by paleo-precipitation since the LGM, suggesting that long-term climate history played an important role in distribution patterns (Rakotoarinivo et al., 2013) For patterns of species diversity based solely on current climate, instability of variables such as water deficit and temperature has been reported to be associated with differences in plant diversity in different biogeographical regions in China (Zhang, Silk & Ma, 2016). On the Qinghai-Tibetan Plateau Yan, Yang & Tang (2013) found that differences in climate were associated with life form and the richness of woody plants was correlated with all climate variables, while that of herbaceous plants was associated with water availability. Moreover, we suggest that life forms, like the geophytes might be less constrained in their response to climate change and persist in areas with changing climate because of their perennating organs.

Monocot geophytes are clearly a functional group, they are not restricted to a certain region, in contrast they are distributed not only in mediterranean areas but also in zones with seasonal climates in low to high elevations and they belong to different lineages, the majority in the most diverse groups, such like Orchidaceae, Amaryllidaceae, Iridaceae and Asparagaceae. Mountain chains in Mexico have a complex array of habitats and pine or oak forests are found in soils with low drainage where some of the geophytes were recorded (Cuéllar-Martínez & Sosa, 2016). Their underground organs that vary in form, size and position permit growth in poor rocky soils or even in gypsum soils (Cuéllar-Martínez & Sosa, 2016). Geophytes represent an interesting group to study adaptations to seasonal and harsh habitats.

We focused in the role that current and historic climate play in patterns of species richness and endemism, however additional abiotic factors such as soils were not addressed and they probably influence these patterns. In Asparagaceae, some geophytes exclusively grow in gypsum soils in the Chihuahuan Desert and thus further research might analyze adaptations of these geophytes to this type of soil. Moreover, biotic interactions like pollination most probably play an important role as well. Many orchids, the most diverse geophyte group in Mesoamerica, are known to have specific pollination systems and they need to be determined to add understanding to the diversity and endemism patterns found in our study.

##  Supplemental Information

10.7717/peerj.3932/supp-1 Supplemental Information 1 Figure S1 Estimated species richness and weighted endemism based on ecological niche modeling for the four most diverse monocot families, considering current climate variables. (A, B) Amaryllidaceae. (C, D) Asparagaceae. (E, F) Iridaceae. (G, H) Orchidaceae.

10.7717/peerj.3932/supp-2 Table S1 List of species and number of occurrence data points used to estimate species richness and endemism using the direct approach based on observed localities

10.7717/peerj.3932/supp-3 Table S2 List of species with unique occurrence data used for species distribution modeling

10.7717/peerj.3932/supp-4 Table S3 Model performance based on AUC and true skill statistic (TSS) for the geophyte taxa which distributions were estimated with species distribution modeling Values are averages of ten cross-validated iterations.

## Additional Information and Declarations

### Data Availability

The following information was supplied regarding data availability: The raw data has been provided in the Supplemental Files.

## References

[ref-1] Abellán P, Svenning J-C (2014). Refugia within refugia—patterns in endemism and genetic divergence are linked to Late Quaternary climate stability in the Iberian Peninsula. Biological Journal of the Linnean Society.

[ref-2] Aguilar-López JL, Pineda E, Luría-Manzano R, Canseco-Márquez L (2016). Species diversity, distribution and conservation status in a Mesoamerican region: amphibians of the Uxpanapa-Chimalapas region, Mexico. Tropical Conservation Science.

[ref-3] Araújo MB, Guisan A (2006). Five (or so) challenges for species distribution. Journal of Biogeography.

[ref-4] Araújo MB, Nogués-Bravo D, Alexandre J, Diniz-Filho F, Haywood AM, Valdes PJ, Rahbek C (2008). Quaternary climate changes explain diversity among reptiles and amphibians. Ecography.

[ref-5] Arita HT, Figueroa F, Frisch A, Rodríguez P, Santos-Del-Prado K (1997). Geographical range size and the conservation of Mexican mammals. Conservation Biology.

[ref-6] Baselga A (2008). Determinants for species richness, endemism and turnover in European longhorn beetles. Ecography.

[ref-7] Bradbury JP (1997). Sources of glacial moisture in Mesoamerica. Quaternary International.

[ref-8] Brooks TM, Mittermeier RA, Da Fonseca GAB, Gerlach J, Hoffman M, Lamoreux JF, Mittermeier CG, Pilgrim JD, Rodrigues ASL (2006). Global biodiversity conservation priorities. Science.

[ref-9] Brown JL (2014). SDMtoolbox: a python-based GIS toolkit for landscape genetic, biogeographic and species distribution model analyses. Methods in Ecology and Evolution.

[ref-10] Corlett RT, Westcott DA (2013). Will plants movements keep up with climate change?. Trends in Ecology and Evolution.

[ref-11] Crisp MD, Laffan S, Linder HP, Monro A (2001). Endemism in the Australian flora. Journal of Biogeography.

[ref-12] Cuéllar-Martínez M, Sosa V (2016). Diversity patterns of monocotyledonous geophytes in Mexico. Botanical Sciences.

[ref-13] Dobrowski SZ, Abatzoglou J, Swanson AK, Greenberg ZA, Schwartz MK (2013). The climate veolocity of the contiguous United States during the 20th century. Global Change Biology.

[ref-14] Dullinger S, Dendoncker N, Gattinger A, Leitner M, Mang T, Moser D, Mücher CA, Plutzar C, Rounsevell M, Willner W, Zimmermann NE, Hülber (2015). Modelling the effect of habitat fragmentation of European forest understorey plants. Diversity and Distributions.

[ref-15] Dynesius M, Jansson R (2000). Evolutionary consequences of changes in species’ geographical distributions driven by Milankovitch climate oscillations. Proceedings of the National Academy of Sciences of the United States of America.

[ref-16] Espejo-Serna A (2012). The endemism in Mexican Liliopsida. Acta Botanica Mexicana.

[ref-17] Feeley KJ, Silman MR (2011). Keep collecting: accurate species distribution modelling requires more collections than previously thought. Diversity and Distribution.

[ref-18] Feng G, Mao L, Dandel B, Swenson NG, Svenning J-C (2015). High plant endemism in China is partially linked to reduced glacial-interglacial climate change. Journal of Biogeography.

[ref-19] Ferrari L, Orozco-Esquivel T, Manea V, Manea M (2012). The dynamic history of the Trans-Mexican Volcanic Belt and the Mexico subduction zone. Tectonophysics.

[ref-20] Ferrusquía-Villafranca I, Ramamoorthy TP, Bye R, Lot A, Fa J (1993). Geology of Mexico: a synopsis. Biological diversity of Mexico: origins and distribution.

[ref-21] Fine PVA (2015). Ecological and evolutionary drivers of geographic variation in species diversity. Annual Review of Ecology and Evolution.

[ref-22] Francis AP, Currie DJ (2003). A globally consistent richness-climate relationship for angiosperms. American Naturalist.

[ref-23] Gándara E, Specht CD, Sosa V (2014). Origin and diversification of the *Milla* clade (Brodiaeoideae, Asparagaceae): a Neotropical group of six geophytic genera. Molecular Phylogenetics and Evolution.

[ref-24] Gavin DG, Fitzpatrick MC, Gugger PF, Heath KD, Rodríguez-Sánchez F, Dobrowski S, Hampe A, Hu FS, Ashcroft MB, Bartlein PJ, Blois JL, Carstens BC, Davis EB, Lafontaine G, Edwards ME, Fernandez M, Henne PD, Herring EM, Holden ZA, Kon W-S, Liu J, Magri D, Matzke NJ, McGloene M, Saltré F, Stigall AL, Tsai Y.HE, Williams JW (2014). Climate refugia: joint inference from fossil records, species distribution models and phylogeography. New Phytologist.

[ref-25] Graham A (1999). The Tertiary history of the northern temperate element in the northern Latin America biota. American Journal of Botany.

[ref-26] Graham CH, Hijmans RJ (2006). A comparison of methods for mapping species ranges and species richness. Global Ecology and Biogeography.

[ref-27] Harrison S, Noss R (2017). Endemism hotspots are linked to stable climatic refugia. Annals of Botany.

[ref-28] Hijmans RJ, Cameron SE, Parra JL, Jones PG, Jarvis A (2005). Very high resolution interpolated climate surfaces for global land areas. International Journal of Climatology.

[ref-29] Hsu RC-C, Tamis WL, Raes N, De Snoo GR, Wolf JHD, Oostermeijer G, Lin S-H (2012). Simulating climate change impacts on forests and associated vascular epiphytes in a subtropical island of East Asia. Diversity and Distributions.

[ref-30] Huang J, Chen B, Liu C, Lai J, Zhang J, Ma K (2012). Identifying hotspots of endemic woody seed plant diversity in China. Diversity and Distributions.

[ref-31] Jablonski D, Kaustuv R, Valentine JW (2006). Out of the tropics: evolutionary dynamics of the latitudinal diversity gradient. Science.

[ref-32] Kamenetsky R, Kamenetsky R, Okubo H (2013). Biodiversity of geophytes, phytogeography, morphology and survival strategies. Ornamental geophytes: from basic science to sustainable production.

[ref-33] Kissling WD, Carl G (2008). Spatial autocorrelation and the selection of simultaneous autoregressive models. Global Ecology and Biogeography.

[ref-34] Kottek M, Grieser J, Beck C, Rudolf B, Rubel F (2006). World map of the Köppen-Geiger climate classification updated. Meteorologische Zeitschrift.

[ref-35] La Comisión Nacional para el Conocimiento y Uso de la Biodiversidad (CONABIO) (2016). Catálogo de autoridades taxonómicas de la flora con distribución en México.

[ref-36] Linder HP (2001a). On areas of endemism with an example from the African Restoniaceae. Systematic Biology.

[ref-37] Linder HP (2001b). Plant diversity and endemism in sub-Saharan tropical Africa. Journal of Biogeography.

[ref-38] Liu C, White M, Newell G (2013). Selecting thresholds for the prediction of species occurrence with presence-only data. Journal of Biogeography.

[ref-39] Loarie SR, Duffy PB, Hamilton H, Asner GP, Field CB, Ackerly DD (2009). The velocity of climate change. Nature.

[ref-40] Ma Z, Sandel B, Svenning J-C (2016). Phylogenetic assemblage structure of North American trees is more strongly shaped by glacial-interglacial climate variability in gymnosperms than in angiosperms. Ecology and Evolution.

[ref-41] Mastretta-Yanez A, Moreno-Letelier A, Piñero D, Jorgensen TH, Emerson BC (2015). Biodiversity in the Mexican highlands and the interaction of geology, geography and climate within the Trans-Mexican Volcanic Belt. Journal of Biogeography.

[ref-42] Metcalfe SE (2006). Late Quaternary environments of the northern deserts and central Transvolcanic Belt of Mexico. Annals of the Missouri Botanical Garden.

[ref-43] Metcalfe SE, O’Hara SL, Caballero M, Davies SJ (2000). Records of late Pleistocene-Holocene climatic change in Mexico: a review. Quaternary Science Reviews.

[ref-44] Meyer C, Weigelt P, Kreft H (2016). Multidimensional biases, gaps and uncertainties in global plant occurrence information. Ecology Letters.

[ref-45] Molina-Venegas R, Aparicio A, Lavergne S, Arroyo J (2016). Climatic and topographical correlates of plant paleo- and neoendemism in a mediterranean biodiversity hotspot. Annals of Botany.

[ref-46] Munguía-Lino G, Vargas-Amado G, Vázquez-García LM, Rodríguez A (2015). Richness and geographic distribution of the Tribe Tigrideae (Iridaceae) in North America. Revista Mexicana de Biodiversidad.

[ref-47] Murray-Smith C, Brummit NA, Oliveira-Filho AT, Bachman S, Moat J, Nic Lughadha EM, Lucas EJ (2009). Plant diversity hotspots in the Atlantic Coastal Forest of Brazil. Conservation Biology.

[ref-48] Noss RF, Platt WJ, Sorrie BA, Weakley AS, Means DB, Constanza J, Peet RK (2015). How global biodiversity hotspots may go unrecognized: lessons from the North American Coastal Plain. Diversity and Distributions.

[ref-49] Ohlemüller R, Anderson BJ, Araújo MB, Butchart SHM, Kudrna O, Ridgely RS, Thomas CD (2008). The coincidence of climatic and species rarity: high risk to small-range species from climate change. Biology Letters.

[ref-50] Olson DM, Dinerstein E, Wikramanayake ED, Burgess ND, Powell GVN, Underwood EC, D’amico JA, Itoua I, Strand HE, Morrison JC, Loucks CJ, Allnutt TF, Ricketts TH, Kura Y, Lamoreux JF, Wettengel WW, Hedao P, Kassem KR (2001). Terrestrial ecoregions of the world: a new map of life on Earth. BioScience.

[ref-51] Parsons RF (2000). Monocotyledonous geophytes: comparison of California with Victoria, Australia. Australian Journal of Botany.

[ref-52] Phillips SJ, Dudík M (2008). Modeling of species distributions with Maxent: new extensions and a comprehensive evaluation. Ecography.

[ref-53] Procheş S, Cowling RM, Goldblatt P, Manning JC, Snijman DA (2006). An overview of the Cape geophytes. Biological Journal of the Linnean Society.

[ref-54] Procheş S, Cowling RM, Du Preez DR (2005). Patterns of geophyte and storage organ size in the winter-rainfall region of southern Africa. Diversity and Distributions.

[ref-55] Rakotoarinivo M, Blach-Overgaard A, Baker WJ, Dransfield J, J. Moat J, Svenning J-C (2013). Palaeo-precipitation is a major determinant of palm species richness patterns across Madagascar: a tropical biodiversity hotspot. Proceedings of the Royal Society B.

[ref-56] Ramírez-Barahona S, Eguiarte LE (2013). The role of glacial cycles in promoting genetic diversity in the Neotropics: the case of cloud forests during the Last Glacial Maximum. Ecology and Evolution.

[ref-57] Rangel TF, Diniz-Filho JAF, Bini LM (2010). SAM: a comprehensive application for spatial analysis in macroecology. Ecography.

[ref-58] Ricklefs RE (1987). Community diversity: relative roles of local and regional processes. Science.

[ref-59] Rzedowski J, Ramamoorthy TP, Bye R, Lot A, Fa J (1993). Diversity and origins of the phanerogamic flora of Mexico. Biological diversity of Mexico: origins and Distribution.

[ref-60] Sandel B, Arge L, Dalsgaard B, Davies RG, Gaston KJ, Sutherland WJ, Svenning J-C (2011). The influence of late Quaternary climate-change velocity on species endemism. Science.

[ref-61] Sanginés-Franco C, Luna-Vega I, Contreras-Medina R, Espinosa D, Tejero-Díez JD, Rivas G (2015). Diversity endemism and conservation of ferns (Polypodiales) in the Mexican mountain component. Journal of Mountain Science.

[ref-62] Sittaro F, Paquette A, Messier C, Nock CA (2017). Tree range expansion in eastern North America fails to keep pace with climate warming at northern range limits. Global Change Biology.

[ref-63] Sosa V, De-Nova JA (2012). Endemic angiosperm lineages in Mexico: hotspots for conservation. Acta Botanica Mexicana.

[ref-64] Svenning J-C, Eiserhardt WL, Normand S, Ordonez A, Sandel B (2015). The influence of Paleoclimate on present-day patterns in biodiversity and ecosystems. Annual Review of Ecology, Evolution and Systematics.

[ref-65] Thiers B (2017). Index Herbariorum. http://sweetgum.nybg.org/science/ih/.

[ref-66] Toledo VM, Prance GT (1982). Pleistocene changes of vegetation in tropical Mexico. Biological diversification in the tropics.

[ref-67] Van Proosdij ASJ, Sosef MSM, Wieringa JJ, Raes N (2016). Minimum required number of specimen records to develop accurate species distribution models. Ecography.

[ref-68] Varela S, Lima-Riberio M, Terribile LC (2015). A short guide to the climate variables of the Last Glacial Maximum for biogeographers. PLOS ONE.

[ref-69] Vargas-Amado G, Castro-Castro A, Harker M, Villaseñor JL, Ortiz E, Rodríguez A (2013). Geographic distribution and richness of the genus *Cosmos* (Asteraceae: Coreopsidae). Revista Mexicana de Biodiversidad.

[ref-70] Villalobos F, Lira-Noriega A, Soberón J, Arita HT (2013). Range-diversity plots for conservation assessments: usi8gn richness and rarity in priority setting. Biological Conservation.

[ref-71] Wiens JJ, Donoghue MJ (2004). Historical biogeography, ecology and species richness. Trends in Ecology and Evolution.

[ref-72] Yan Y, Yang X, Tang Z (2013). Patterns of species diversity and phylogenetic structure of vascular plants on the Qinghai-Tibetan Plateau. Ecology and Evolution.

[ref-73] Zhang M-G, Silk JWF, Ma K-P (2016). Using species distribution modeling to delineate the botanical richness patterns and phytogeographical regions of China. Scientific Reports.

