# Supplementary figures and images for: Influence of current climate, historical climate stability and topography on species richness and endemism in Mesoamerican geophyte plants
Source: PeerJ. 2017 Oct 20;5:e3932. doi: 10.7717/peerj.3932 (PMC5652257; doi:10.7717/peerj.3932)

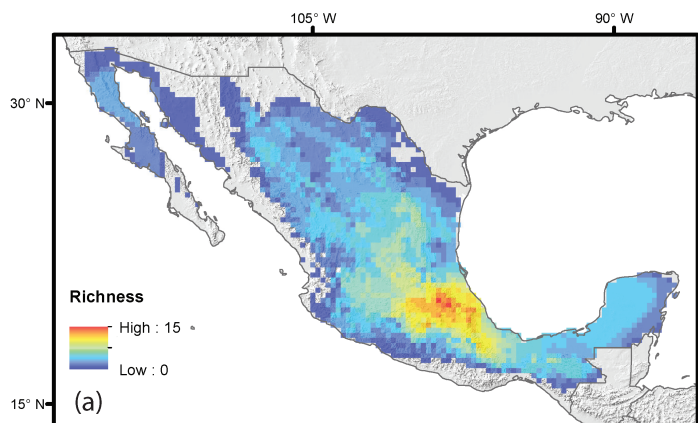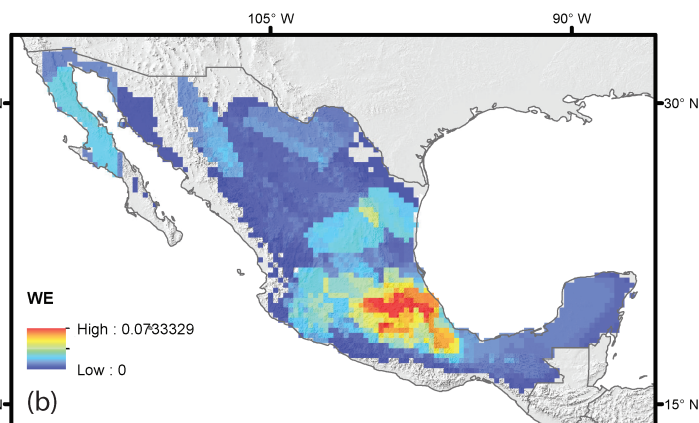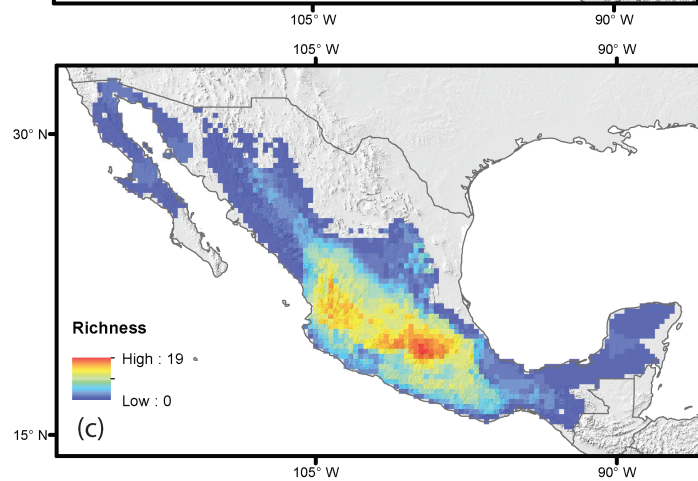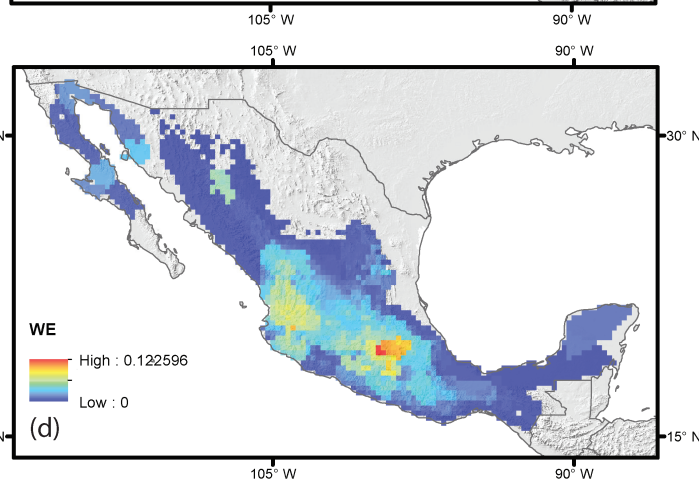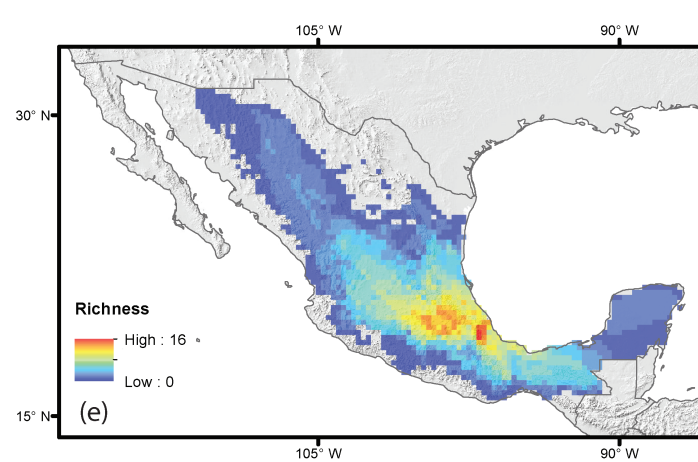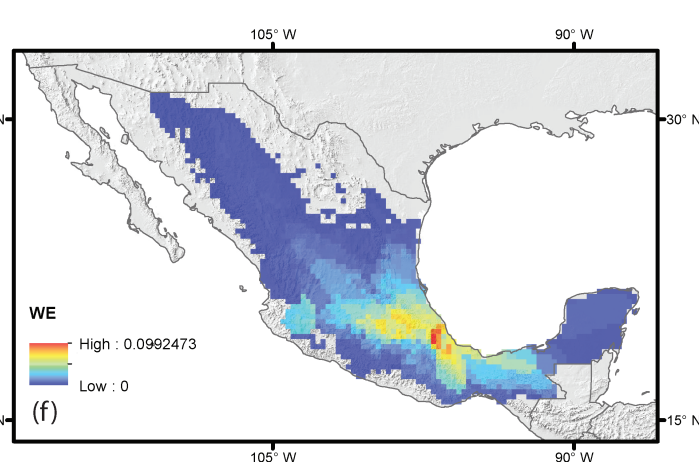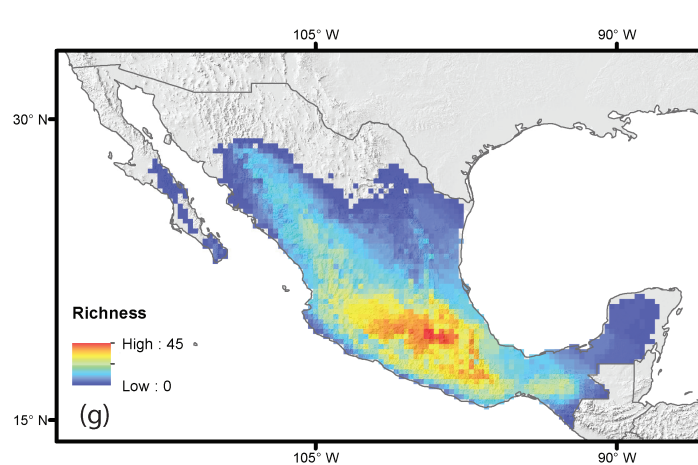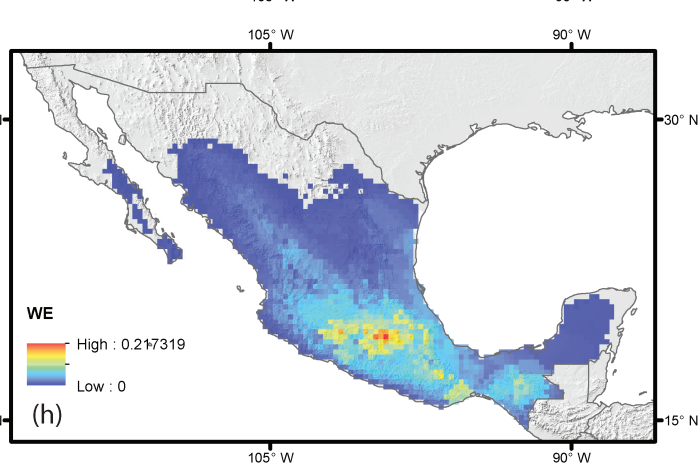

Supplement: Supplemental Information 1 — Estimated species richness and weighted endemism based on ecological niche modeling for the four most diverse monocot families, considering current climate variables. (A, B) Amaryllidaceae. (C, D) Asparagaceae. (E, F) Iridaceae. (G, H) Orchidaceae. [file peerj-05-3932-s001.pdf]
