# Supplementary material for: Influence of current climate, historical climate stability and topography on species richness and endemism in Mesoamerican geophyte plants
Source: PeerJ. 2017 Oct 20;5:e3932. doi: 10.7717/peerj.3932 (PMC5652257; doi:10.7717/peerj.3932)
Supplement: Table S1 [file peerj-05-3932-s002.pdf]

Table S1. List of studied species with their georeferences.

Record numb	Family	Species	lat.
1	Hypoxidaceae	Hypoxys mexicana	26.7933333
2	Hypoxidaceae	Hypoxys mexicana	19.1441667
3	Hypoxidaceae	Hypoxys mexicana	18.2547222
4	Hypoxidaceae	Hypoxys mexicana	19.5030556
5	Hypoxidaceae	Hypoxys mexicana	19.3425
6	Hypoxidaceae	Hypoxys mexicana	19.7680556
7	Hypoxidaceae	Hypoxys mexicana	20.1297222
8	Hypoxidaceae	Hypoxys mexicana	19.4180556
9	Hypoxidaceae	Hypoxys mexicana	19.2022222
10	Hypoxidaceae	Hypoxys mexicana	19.6447222
11	Hypoxidaceae	Hypoxys mexicana	19.7211111
12	Hypoxidaceae	Hypoxys mexicana	28.175
13	Hypoxidaceae	Hypoxys mexicana	27.3166667
14	Hypoxidaceae	Hypoxys mexicana	28.3083333
15	Hypoxidaceae	Hypoxys mexicana	23.9786111
16	Hypoxidaceae	Hypoxys mexicana	19.4216667
17	Hypoxidaceae	Hypoxys mexicana	21.5166667
18	Hypoxidaceae	Hypoxys mexicana	19.705
19	Hypoxidaceae	Hypoxys mexicana	22.97
20	Hypoxidaceae	Hypoxys mexicana	18.998952
21	Hypoxidaceae	Hypoxys mexicana	21.7633333
22	Hypoxidaceae	Hypoxys mexicana	23.2469444
23	Hypoxidaceae	Hypoxys mexicana	21.5716667
24	Hypoxidaceae	Hypoxis lucens	21.977705
25	Hypoxidaceae	Hypoxis lucens	20.4275
26	Hypoxidaceae	Hypoxis lucens	19.5113889
27	Hypoxidaceae	Hypoxys decumbens	21.9422222
28	Hypoxidaceae	Hypoxys decumbens	19.285535
29	Hypoxidaceae	Hypoxys decumbens	19.559529
30	Hypoxidaceae	Hypoxys decumbens	18.8497222
31	Hypoxidaceae	Hypoxys decumbens	19.536662
32	Hypoxidaceae	Hypoxys decumbens	19.637911
33	Hypoxidaceae	Hypoxys decumbens	19.0816667
34	Hypoxidaceae	Hypoxys decumbens	19.405247
35	Hypoxidaceae	Hypoxys decumbens	19.4775
36	Hypoxidaceae	Hypoxys decumbens	18.9608333
37	Hypoxidaceae	Hypoxys decumbens	18.9275
38	Hypoxidaceae	Hypoxys decumbens	19.025
39	Hypoxidaceae	Hypoxys decumbens	23.7791667
40	Hypoxidaceae	Hypoxys decumbens	21.2441667
41	Hypoxidaceae	Hypoxys decumbens	22.8841806
42	Hypoxidaceae	Hypoxys decumbens	21.8772194
43	Hypoxidaceae	Hypoxys decumbens	19.203979

44	Hypoxidaceae	Hypoxys decumbens	21.0242611
45	Hypoxidaceae	Hypoxys decumbens	18.3129861
46	Hypoxidaceae	Hypoxys decumbens	17.2915444
47	Hypoxidaceae	Hypoxys decumbens	19.4333333
48	Hypoxidaceae	Hypoxys decumbens	23.9972222
49	Hypoxidaceae	Hypoxys decumbens	16.8336111
50	Hypoxidaceae	Hypoxys decumbens	24.1629556
51	Hypoxidaceae	Hypoxys decumbens	17.6941667
52	Hypoxidaceae	Hypoxis hirsuta	19.469748
53	Hypoxidaceae	Hypoxis fibrata	20.2916667
54	Hypoxidaceae	Hypoxys decumbens	17.5775
55	Hypoxidaceae	Hypoxis pulchella	24.0166667
56	Hypoxidaceae	Hypoxis pulchella	25.4429667
57	Hypoxidaceae	Hypoxis pulchella	25.2166667
58	Hypoxidaceae	Hypoxis tepicensis	19.0369389
59	Hypoxidaceae	Hypoxis wrightii	20.9622222
60	Hypoxidaceae	Hypoxis tepicensis	19.4333333
61	Hypoxidaceae	Hypoxis potosina	22.5
62	Hypoxidaceae	Hypoxis potosina	18.987
63	Hypoxidaceae	Hypoxis potosina	18.863
64	Hypoxidaceae	Hypoxis potosina	19.177
65	Hypoxidaceae	Hypoxis potosina	20.283
66	Hypoxidaceae	Hypoxis potosina	19.903
67	Hypoxidaceae	Hypoxis colliculata	17.561656
68	Hypoxidaceae	Hypoxis colliculata	16.710977
69	Hypoxidaceae	Hypoxis colliculata	16.742662
70	Hypoxidaceae	Hypoxis colliculata	16.505816
71	Hypoxidaceae	Hypoxis colliculata	17.519412
72	Hypoxidaceae	Hypoxis colliculata	17.537078
73	Amaryllidaceae	Allium cernuum	29.0916056
74	Amaryllidaceae	Allium cernuum	29.3504611
75	Amaryllidaceae	Allium cernuum	29.05
76	Amaryllidaceae	Allium cernuum	27.4325
77	Amaryllidaceae	Allium drummondii	26.854748
78	Amaryllidaceae	Allium drummondii	29.526831
79	Amaryllidaceae	Allium durangoense	37.271412
80	Amaryllidaceae	Allium eurotophilum	30.916188
81	Amaryllidaceae	Allium eurotophilum	30.906649
82	Amaryllidaceae	Allium eurotophilum	30.999113
83	Amaryllidaceae	Allium eurotophilum	30.965805
84	Amaryllidaceae	Allium eurotophilum	31.4
85	Amaryllidaceae	Allium eurotophilum	31.05
86	Amaryllidaceae	Allium eurotophilum	26.9275
87	Amaryllidaceae	Allium fantasmosense	22.232639
88	Amaryllidaceae	Allium fimbriatum	32.4333333

89	Amaryllidaceae	Allium fimbriatum	32.0333333
90	Amaryllidaceae	Allium glandulosum	22.0797222
91	Amaryllidaceae	Allium glandulosum	21.3283333
92	Amaryllidaceae	Allium glandulosum	20.2175
93	Amaryllidaceae	Allium glandulosum	19.7
94	Amaryllidaceae	Allium glandulosum	20.2841667
95	Amaryllidaceae	Allium glandulosum	16.7141667
96	Amaryllidaceae	Allium glandulosum	19.587289
97	Amaryllidaceae	Allium glandulosum	22.1547222
98	Amaryllidaceae	Allium glandulosum	19.5775
99	Amaryllidaceae	Allium glandulosum	22.0775
100	Amaryllidaceae	Allium glandulosum	18.515505
101	Amaryllidaceae	Allium glandulosum	20.420249
102	Amaryllidaceae	Allium glandulosum	19.713401
103	Amaryllidaceae	Allium glandulosum	18.8113889
104	Amaryllidaceae	Allium glandulosum	19.2775
105	Amaryllidaceae	Allium glandulosum	19.5966667
106	Amaryllidaceae	Allium glandulosum	19.5275
107	Amaryllidaceae	Allium glandulosum	19.6941667
108	Amaryllidaceae	Allium glandulosum	20.3441667
109	Amaryllidaceae	Allium glandulosum	19.7441667
110	Amaryllidaceae	Allium glandulosum	19.0941667
111	Amaryllidaceae	Allium glandulosum	19.3275
112	Amaryllidaceae	Allium glandulosum	18.9441667
113	Amaryllidaceae	Allium glandulosum	20.0441667
114	Amaryllidaceae	Allium glandulosum	20.9441667
115	Amaryllidaceae	Allium glandulosum	19.8441667
116	Amaryllidaceae	Allium glandulosum	20.7775
117	Amaryllidaceae	Allium glandulosum	15.1441667
118	Amaryllidaceae	Allium glandulosum	20.0991667
119	Amaryllidaceae	Allium glandulosum	19.7680556
120	Amaryllidaceae	Allium glandulosum	19.8730556
121	Amaryllidaceae	Allium glandulosum	19.8455556
122	Amaryllidaceae	Allium glandulosum	19.4455556
123	Amaryllidaceae	Allium glandulosum	19.3063889
124	Amaryllidaceae	Allium glandulosum	20.185
125	Amaryllidaceae	Allium glandulosum	19.2083333
126	Amaryllidaceae	Allium glandulosum	19.5519444
127	Amaryllidaceae	Allium glandulosum	19.32
128	Amaryllidaceae	Allium glandulosum	19.2013889
129	Amaryllidaceae	Allium glandulosum	19.5366667
130	Amaryllidaceae	Allium glandulosum	19.8425
131	Amaryllidaceae	Allium glandulosum	19.5052778
132	Amaryllidaceae	Allium glandulosum	19.37
133	Amaryllidaceae	Allium glandulosum	19.5719444

134	Amaryllidaceae	Allium glandulosum	19.3083333
135	Amaryllidaceae	Allium glandulosum	19.2252778
136	Amaryllidaceae	Allium glandulosum	19.6333333
137	Amaryllidaceae	Allium glandulosum	19.3425
138	Amaryllidaceae	Allium glandulosum	19.5322222
139	Amaryllidaceae	Allium glandulosum	19.3061111
140	Amaryllidaceae	Allium glandulosum	17.2261111
141	Amaryllidaceae	Allium glandulosum	23.2208333
142	Amaryllidaceae	Allium glandulosum	24.5166667
143	Amaryllidaceae	Allium glandulosum	30.05
144	Amaryllidaceae	Allium glandulosum	20.0998472
145	Amaryllidaceae	Allium glandulosum	22.1639
146	Amaryllidaceae	Allium glandulosum	20.3811389
147	Amaryllidaceae	Allium glandulosum	25.6711111
148	Amaryllidaceae	Allium glandulosum	23.9857472
149	Amaryllidaceae	Allium glandulosum	20.9294667
150	Amaryllidaceae	Allium glandulosum	25.5833333
151	Amaryllidaceae	Allium glandulosum	20.3716667
152	Amaryllidaceae	Allium glandulosum	28.5584306
153	Amaryllidaceae	Allium glandulosum	19.0819389
154	Amaryllidaceae	Allium glandulosum	19.2161111
155	Amaryllidaceae	Allium glandulosum	19.5719389
156	Amaryllidaceae	Allium glandulosum	20.3433333
157	Amaryllidaceae	Allium glandulosum	19.4047222
158	Amaryllidaceae	Allium glandulosum	18.1680556
159	Amaryllidaceae	Allium glandulosum	20.5083333
160	Amaryllidaceae	Allium glandulosum	20.1233333
161	Amaryllidaceae	Allium glandulosum	19.2166667
162	Amaryllidaceae	Allium glandulosum	24.4183333
163	Amaryllidaceae	Allium glandulosum	26.1133333
164	Amaryllidaceae	Allium glandulosum	19.9563889
165	Amaryllidaceae	Allium glandulosum	22.3533333
166	Amaryllidaceae	Allium glandulosum	19.9133333
167	Amaryllidaceae	Allium glandulosum	20.4166667
168	Amaryllidaceae	Allium glandulosum	19.3416667
169	Amaryllidaceae	Allium glandulosum	17.4616667
170	Amaryllidaceae	Allium glandulosum	20.5316667
171	Amaryllidaceae	Allium glandulosum	22.2513889
172	Amaryllidaceae	Allium glandulosum	18.85
173	Amaryllidaceae	Allium glandulosum	15.0625
174	Amaryllidaceae	Allium glandulosum	24.025
175	Amaryllidaceae	Allium glandulosum	17.2683333
176	Amaryllidaceae	Allium glandulosum	23.6316667
177	Amaryllidaceae	Allium glandulosum	29.1083333
178	Amaryllidaceae	Allium glandulosum	19.1075

179	Amaryllidaceae	Allium glandulosum	19.53
180	Amaryllidaceae	Allium glandulosum	20.1966667
181	Amaryllidaceae	Allium glandulosum	22.1347222
182	Amaryllidaceae	Allium glandulosum	19.4666667
183	Amaryllidaceae	Allium glandulosum	20.3366667
184	Amaryllidaceae	Allium glandulosum	21.05
185	Amaryllidaceae	Allium glandulosum	19.5716667
186	Amaryllidaceae	Allium glandulosum	21.2986111
187	Amaryllidaceae	Allium glandulosum	21.015
188	Amaryllidaceae	Allium glandulosum	21.3683333
189	Amaryllidaceae	Allium glandulosum	28.4030556
190	Amaryllidaceae	Allium glandulosum	17.4333333
191	Amaryllidaceae	Allium glandulosum	23.121129
192	Amaryllidaceae	Allium glandulosum	17.7108333
193	Amaryllidaceae	Allium glandulosum	18.1275
194	Amaryllidaceae	Allium glandulosum	18.5108333
195	Amaryllidaceae	Allium glandulosum	18.2941667
196	Amaryllidaceae	Allium glandulosum	18.2941667
197	Amaryllidaceae	Allium glandulosum	31.9
198	Amaryllidaceae	Allium glandulosum	28.1583333
199	Amaryllidaceae	Allium haematochiton	27.54
200	Amaryllidaceae	Allium haematochiton	29.06
201	Amaryllidaceae	Allium haematochiton	27.54
202	Amaryllidaceae	Allium haematochiton	29.06
203	Amaryllidaceae	Allium haematochiton	26.9775
204	Amaryllidaceae	Allium haematochiton	32.505218
205	Amaryllidaceae	Allium haematochiton	29.0169444
206	Amaryllidaceae	Allium haematochiton	32.336424
207	Amaryllidaceae	Allium haematochiton	32.0333333
208	Amaryllidaceae	Allium haematochiton	30.965805
209	Amaryllidaceae	Allium hintoniorum	24.113
210	Amaryllidaceae	Allium hintoniorum	25.2166667
211	Amaryllidaceae	Allium hintoniorum	24.7533306
212	Amaryllidaceae	Allium hintoniorum	23.9786111
213	Amaryllidaceae	Allium huntiae	24.02
214	Amaryllidaceae	Allium mannii	37.290111
215	Amaryllidaceae	Allium melliferum	20.399999
216	Amaryllidaceae	Allium mexicanum	24.049556
217	Amaryllidaceae	Allium ownbeyi	26.14736
218	Amaryllidaceae	Allium peninsulare	32.5627778
219	Amaryllidaceae	Allium peninsulare	29.9720667
220	Amaryllidaceae	Allium plummerae	30.9608333
221	Amaryllidaceae	Allium plummerae	30.9608333
222	Amaryllidaceae	Allium plummerae	22.15
223	Amaryllidaceae	Allium praecox	31.5775

224	Amaryllidaceae	Allium praecox	30.05
225	Amaryllidaceae	Allium pueblanum	18.314541
226	Amaryllidaceae	Allium rhizomatum	28.2780556
227	Amaryllidaceae	Allium rhizomatum	19.3700083
228	Amaryllidaceae	Allium rhizomatum	29.7588889
232	Amaryllidaceae	Allium rhizomatum	23.62
233	Amaryllidaceae	Allium stoloniferum	20.2608333
234	Amaryllidaceae	Allium stoloniferum	20.185
235	Amaryllidaceae	Allium stoloniferum	19.3488889
236	Amaryllidaceae	Allium stoloniferum	18.4883306
237	Amaryllidaceae	Allium telaponense	19.379219
238	Amaryllidaceae	Crinum americanum	19.513813
239	Amaryllidaceae	Crinum americanum	20.975766
240	Amaryllidaceae	Crinum americanum	19.118976
241	Amaryllidaceae	Crinum americanum	18.739511
242	Amaryllidaceae	Crinum americanum	19.595566
243	Amaryllidaceae	Crinum americanum	15.1941667
244	Amaryllidaceae	Crinum americanum	18.49613
245	Amaryllidaceae	Crinum americanum	17.7275
246	Amaryllidaceae	Crinum americanum	19.0516667
248	Amaryllidaceae	Crinum americanum	17.7775
249	Amaryllidaceae	Crinum americanum	18.8108333
250	Amaryllidaceae	Crinum americanum	20.3355556
252	Amaryllidaceae	Habranthus arenicola	24.719666
257	Amaryllidaceae	Habranthus chichimeca	23.935884
258	Amaryllidaceae	Habranthus chichimeca	23.895209
259	Amaryllidaceae	Habranthus chichimeca	23.342617
260	Amaryllidaceae	Habranthus chichimeca	23.128354
275	Amaryllidaceae	Zephyranthes conzattii	17.269945
276	Amaryllidaceae	Habranthus longifolius	31.037401
277	Amaryllidaceae	Habranthus longifolius	28.495316
278	Amaryllidaceae	Habranthus longifolius	25.921617
279	Amaryllidaceae	Habranthus longifolius	22.2108
280	Amaryllidaceae	Habranthus longifolius	24.782273
281	Amaryllidaceae	Habranthus medinae	18.194843
282	Amaryllidaceae	Habranthus mexicanus	21.111593
283	Amaryllidaceae	Habranthus vittatus	17.858331
284	Amaryllidaceae	Nothoscordum bivalve	19.8441667
285	Amaryllidaceae	Nothoscordum bivalve	19.7861111
286	Amaryllidaceae	Nothoscordum bivalve	21.3108333
287	Amaryllidaceae	Nothoscordum bivalve	19.6441667
288	Amaryllidaceae	Nothoscordum bivalve	21.8608333
289	Amaryllidaceae	Nothoscordum bivalve	19.3441667
290	Amaryllidaceae	Nothoscordum bivalve	20.8608333
291	Amaryllidaceae	Nothoscordum bivalve	19.5083333

292	Amaryllidaceae	Nothoscordum bivalve	20.2275
293	Amaryllidaceae	Nothoscordum bivalve	19.596234
294	Amaryllidaceae	Nothoscordum bivalve	19.3061111
295	Amaryllidaceae	Nothoscordum bivalve	25.2836028
296	Amaryllidaceae	Nothoscordum bivalve	25.3496556
297	Amaryllidaceae	Nothoscordum bivalve	29.1
298	Amaryllidaceae	Nothoscordum bivalve	23.4556667
299	Amaryllidaceae	Nothoscordum bivalve	23.9857472
300	Amaryllidaceae	Nothoscordum bivalve	25.85
301	Amaryllidaceae	Nothoscordum bivalve	27.8302333
302	Amaryllidaceae	Nothoscordum bivalve	16.7479417
303	Amaryllidaceae	Nothoscordum bivalve	18.1755944
304	Amaryllidaceae	Nothoscordum bivalve	24.1042083
305	Amaryllidaceae	Nothoscordum bivalve	28.3794444
306	Amaryllidaceae	Nothoscordum bivalve	17.3463889
307	Amaryllidaceae	Nothoscordum bivalve	22.1347222
308	Amaryllidaceae	Nothoscordum bivalve	18.9616667
309	Amaryllidaceae	Nothoscordum bivalve	23.3836111
310	Amaryllidaceae	Nothoscordum gracile	20.0694444
311	Amaryllidaceae	Nothoscordum gracile	25.85
312	Amaryllidaceae	Nothoscordum gracile	16.7038889
313	Amaryllidaceae	Nothoscordum gracile	29.9833
314	Amaryllidaceae	Nothoscordum gracile	25
315	Amaryllidaceae	Nothoscordum gracile	24.0333337
316	Amaryllidaceae	Nothoscordum gracile	19.01666
317	Amaryllidaceae	Nothoscordum gracile	19.39972
318	Amaryllidaceae	Nothoscordum gracile	24.59
319	Amaryllidaceae	Nothoscordum gracile	18.8472222
320	Amaryllidaceae	Nothoscordum gracile	22.15
322	Amaryllidaceae	Hymenocallis acutifolia	19.3186833
323	Amaryllidaceae	Hymenocallis acutifolia	18.905
324	Amaryllidaceae	Hymenocallis acutifolia	21.865
325	Amaryllidaceae	Hymenocallis acutifolia	21.5166667
326	Amaryllidaceae	Hymenocallis acutifolia	18.13333
327	Amaryllidaceae	Hynemocallis astrotephana	17.591397
328	Amaryllidaceae	Hymenocallis azteciana	20.3530556
329	Amaryllidaceae	Hymenocallis azteciana	21.3608333
330	Amaryllidaceae	Hymenocallis cleo	16.69857
331	Amaryllidaceae	Hymenocallis clivorum	28.4669444
332	Amaryllidaceae	Hymenocallis concinna	18.48024
333	Amaryllidaceae	Hymenocallis glauca	18.5386111
334	Amaryllidaceae	Hymenocallis glauca	17.942784
335	Amaryllidaceae	Hymenocallis glauca	18.9666667
336	Amaryllidaceae	Hymenocallis graminifolia	18.9408333
337	Amaryllidaceae	Hymenocallis graminifolia	18.9066667

338	Amaryllidaceae	Hymenocallis guerreroensis	17.6275
339	Amaryllidaceae	Hymenocallis guerreroensis	17.6516667
340	Amaryllidaceae	Hymenocallis harrisiana	19.6819444
341	Amaryllidaceae	Hymenocallis harrisiana	17.9608333
342	Amaryllidaceae	Hymenocallis harrisiana	19.4855556
343	Amaryllidaceae	Hymenocallis harrisiana	18.9866667
344	Amaryllidaceae	Hymenocallis harrisiana	19.0166667
345	Amaryllidaceae	Hymenocallis harrisiana	18.2108333
346	Amaryllidaceae	Hymenocallis howardii	20.2275
347	Amaryllidaceae	Hymenocallis howardii	21.2113889
348	Amaryllidaceae	Hymenocallis howardii	20.9066667
349	Amaryllidaceae	Hymenocallis jaliscensis	19.5583333
350	Amaryllidaceae	Hymenocallis jaliscensis	20.28333
351	Amaryllidaceae	Hymenocallis leavenworthii	19.45
352	Amaryllidaceae	Hymenocallis leavenworthii	19.5466667
353	Amaryllidaceae	Hymenocallis littoralis	18.1416667
354	Amaryllidaceae	Hymenocallis littoralis	18.8775
355	Amaryllidaceae	Hymenocallis littoralis	19.2108333
356	Amaryllidaceae	Hymenocallis littoralis	16.9275
357	Amaryllidaceae	Hymenocallis littoralis	19.8516667
358	Amaryllidaceae	Hymenocallis littoralis	20.138614
359	Amaryllidaceae	Hymenocallis littoralis	18.0985444
360	Amaryllidaceae	Hymenocallis littoralis	21.179694
361	Amaryllidaceae	Hymenocallis littoralis	20.5375
362	Amaryllidaceae	Hymenocallis littoralis	20.6916667
363	Amaryllidaceae	Hymenocallis partita	16.12
364	Amaryllidaceae	Hymenocallis pimana	28.4275
365	Amaryllidaceae	Hymenocallis pimana	28.7111111
366	Amaryllidaceae	Hymenocallis pimana	28.4952778
367	Amaryllidaceae	Hymenocallis proterantha	18.6363889
368	Amaryllidaceae	Hymenocallis proterantha	19.7708333
369	Amaryllidaceae	Hymenocallis proterantha	21.5166667
370	Amaryllidaceae	Hymenocallis pumila	19.2516667
371	Amaryllidaceae	Hymenocallis pumila	19.6733333
372	Amaryllidaceae	Hymenocallis sonorensis	27.0083333
373	Amaryllidaceae	Hymenocallis sonorensis	28.5726361
374	Amaryllidaceae	Hymenocallis sonorensis	26.9125
375	Amaryllidaceae	Hymenocallis sonorensis	30.7027778
376	Amaryllidaceae	Hymenocallis vasconcelosii	17.404318
377	Amaryllidaceae	Hymenocallis woelfleana	21.1439889
392	Amaryllidaceae	Sprekelia formosissima	20.1652778
393	Amaryllidaceae	Sprekelia formosissima	19.5883333
394	Amaryllidaceae	Sprekelia formosissima	19.5275
395	Amaryllidaceae	Sprekelia formosissima	18.8483333
396	Amaryllidaceae	Sprekelia formosissima	20.20812

397	Amaryllidaceae	Sprekelia formosissima	21.3275
398	Amaryllidaceae	Sprekelia formosissima	17.9941667
399	Amaryllidaceae	Sprekelia formosissima	17.8275
400	Amaryllidaceae	Sprekelia formosissima	23.3466667
401	Amaryllidaceae	Sprekelia formosissima	19.7702778
402	Amaryllidaceae	Sprekelia formosissima	19.4197222
403	Amaryllidaceae	Sprekelia formosissima	20.0625
404	Amaryllidaceae	Sprekelia formosissima	19.2013889
405	Amaryllidaceae	Sprekelia formosissima	19.2875
406	Amaryllidaceae	Sprekelia formosissima	19.7211111
407	Amaryllidaceae	Sprekelia formosissima	18.0333333
408	Amaryllidaceae	Sprekelia formosissima	19.3884
409	Amaryllidaceae	Sprekelia formosissima	26.1181528
410	Amaryllidaceae	Sprekelia formosissima	25.0833333
411	Amaryllidaceae	Sprekelia formosissima	17.4716667
412	Amaryllidaceae	Sprekelia formosissima	19.7713083
413	Amaryllidaceae	Sprekelia formosissima	18.9366667
414	Amaryllidaceae	Sprekelia formosissima	19.855
415	Amaryllidaceae	Sprekelia formosissima	18.1680556
416	Amaryllidaceae	Sprekelia formosissima	21.7416667
417	Amaryllidaceae	Sprekelia formosissima	21.175
419	Amaryllidaceae	Zephyranthes clintiae	21.127779
420	Amaryllidaceae	Zephyranthes clintiae	19.556361
421	Amaryllidaceae	Zephyranthes clintiae	21.1108083
422	Amaryllidaceae	Zephyranthes clintiae	20.15186
423	Amaryllidaceae	Zephyranthes lindleyana	18.8497222
424	Amaryllidaceae	Zephyranthes lindleyana	22.4441667
425	Amaryllidaceae	Zephyranthes lindleyana	25.6711111
426	Amaryllidaceae	Zephyranthes lindleyana	20.4733333
427	Amaryllidaceae	Zephyranthes lindleyana	20.018973
428	Amaryllidaceae	Zephyranthes lindleyana	18.1108333
429	Amaryllidaceae	Zephyranthes lindleyana	20.58201
430	Amaryllidaceae	Zephyranthes miradorensis	19.513813
431	Amaryllidaceae	Zephyranthes nelsonii	16.67305
432	Amaryllidaceae	Zephyranthes bella	22.637178
433	Amaryllidaceae	Zephyranthes brevipes	20.1297222
434	Amaryllidaceae	Zephyranthes brevipes	19.8355556
435	Amaryllidaceae	Zephyranthes brevipes	19.788444
436	Amaryllidaceae	Zephyranthes brevipes	19.7702778
437	Amaryllidaceae	Zephyranthes brevipes	18.9852806
438	Amaryllidaceae	Zephyranthes brevipes	19.5294444
439	Amaryllidaceae	Zephyranthes carinata	19.355472
440	Amaryllidaceae	Zephyranthes carinata	19.9441667
441	Amaryllidaceae	Zephyranthes carinata	19.513813
442	Amaryllidaceae	Zephyranthes carinata	19.045846

443	Amaryllidaceae	Zephyranthes carinata	18.98
444	Amaryllidaceae	Zephyranthes carinata	19.6183333
445	Amaryllidaceae	Zephyranthes carinata	21.052134
446	Amaryllidaceae	Zephyranthes carinata	21.355421
447	Amaryllidaceae	Zephyranthes carinata	20.063359
448	Amaryllidaceae	Zephyranthes carinata	20.5
449	Amaryllidaceae	Zephyranthes carinata	17.7608333
450	Amaryllidaceae	Zephyranthes carinata	20.9669444
451	Amaryllidaceae	Zephyranthes carinata	16.2913889
452	Amaryllidaceae	Zephyranthes citrina	21.58397
453	Amaryllidaceae	Zephyranthes citrina	19.9313889
454	Amaryllidaceae	Zephyranthes latissimifolia	20.361776
455	Amaryllidaceae	Zephyranthes leucantha	20.850147
456	Amaryllidaceae	Zephyranthes moctezumae	21.254003
457	Amaryllidaceae	Zephyranthes morrisclintii	24.0994389
458	Amaryllidaceae	Zephyranthes morrisclintii	24.7827778
459	Amaryllidaceae	Zephyranthes nymphaea	22.534928
460	Amaryllidaceae	Zephyranthes primulina	21.670475
461	Amaryllidaceae	Zephyranthes primulina	23.23
462	Amaryllidaceae	Zephyranthes reginae	21.662942
463	Amaryllidaceae	Zephyranthes sessilis	18.0667
464	Amaryllidaceae	Zephyranthes subflava	22.372468
465	Amaryllidaceae	Zephyranthes subflava	22.541003
466	Asparagaceae	Chlorogalum parviflorum	32.5666667
467	Asparagaceae	Muilla maritima	32.1108333
468	Asparagaceae	Muilla maritima	26.746528
469	Asparagaceae	Muilla maritima	30.9833333
470	Asparagaceae	Bloomeria crocea	30.9666667
471	Asparagaceae	Bloomeria crocea	32.3166667
472	Asparagaceae	Dichelostemma capitatum	32.0833333
473	Asparagaceae	Dichelostemma capitatum	29.5666667
474	Asparagaceae	Dichelostemma capitatum	28.8655556
475	Asparagaceae	Triteleiopsis palmeri	29.378225
476	Asparagaceae	Triteleiopsis palmeri	27.362831
477	Asparagaceae	Triteleiopsis palmeri	28.9833333
478	Asparagaceae	Triteleiopsis palmeri	29.525
479	Asparagaceae	Triteleiopsis palmeri	32.3616667
480	Asparagaceae	Chlorogalum parviflorum	32.3666667
481	Asparagaceae	Triteleia guadalupensis	29.043966
482	Iridaceae	Ainea conzattii	17.2733
483	Iridaceae	Alophia drummondii	19.370098
484	Iridaceae	Alophia drummondii	16.4758139
485	Iridaceae	Alophia drummondii	16.5284833
486	Iridaceae	Alophia drummondii	23.359737
487	Iridaceae	Alophia drummondii	21.640101

488	Iridaceae	Alophia drummondii	24.3375
489	Iridaceae	Alophia drummondii	21.6344444
490	Iridaceae	Alophia veracruzana	22.1861111
491	Iridaceae	Alophia veracruzana	19.523122
492	Iridaceae	Alophia silvestris	19.2275
493	Iridaceae	Alophia silvestris	18.2180556
494	Iridaceae	Alophia silvestris	17.835
495	Iridaceae	Cardiostigma longispatha	19.1933333
496	Iridaceae	Cardiostigma longispatha	23.0116667
497	Iridaceae	Cardiostigma longispatha	23.4775
498	Iridaceae	Cardiostigma longispatha	18.9408333
499	Iridaceae	Cardiostigma longispatha	18.85
500	Iridaceae	Cardiostigma longispatha	19.798169
501	Iridaceae	Cardiostigma longispatha	22.190981
502	Iridaceae	Cardiostigma longispatha	18.614531
503	Iridaceae	Cardiostigma mexicana	18.9263889
504	Iridaceae	Cardiostigma hintonii	20.061653
505	Iridaceae	Cipura campanulata	18.905773
506	Iridaceae	Cipura campanulata	18.811409
507	Iridaceae	Cipura campanulata	20.7454833
508	Iridaceae	Cipura campanulata	19.9888889
509	Iridaceae	Cipura campanulata	17.5360806
510	Iridaceae	Cipura campanulata	17.9755611
511	Iridaceae	Cipura campanulata	18.7355611
512	Iridaceae	Cipura campanulata	18.5386111
513	Iridaceae	Cipura paludosa	19.8333333
514	Iridaceae	Cipura paludosa	18.2583333
515	Iridaceae	Cipura paludosa	20.8108333
516	Iridaceae	Cipura paludosa	20.2608333
517	Iridaceae	Cipura paludosa	18.4775
518	Iridaceae	Cipura paludosa	20.8275
519	Iridaceae	Cipura paludosa	17.8275
520	Iridaceae	Cipura paludosa	18.49613
521	Iridaceae	Cipura paludosa	16.5065333
522	Iridaceae	Cipura paludosa	21.0433333
523	Iridaceae	Cipura paludosa	21.2366667
524	Iridaceae	Cipura paludosa	21.5166667
525	Iridaceae	Cipura paludosa	18.9166667
526	Iridaceae	Cipura paludosa	19.7666667
527	Iridaceae	Cipura paludosa	16.1333333
528	Iridaceae	Cipura paludosa	19.0111111
529	Iridaceae	Cipura paludosa	20.8922222
530	Iridaceae	Cipura paludosa	21.0875
531	Iridaceae	Eleutherine latifolia	19.597526
532	Iridaceae	Eleutherine latifolia	18.5408333

533	Iridaceae	Eleutherine latifolia	19.400166
534	Iridaceae	Eleutherine latifolia	21.34
535	Iridaceae	Eleutherine latifolia	23.3177806
536	Iridaceae	Eleutherine latifolia	22.488075
537	Iridaceae	Eleutherine latifolia	18.085
538	Iridaceae	Eleutherine latifolia	21.2216667
539	Iridaceae	Eleutherine bulbosa	19.36262
540	Iridaceae	Eleutherine bulbosa	18.6941667
541	Iridaceae	Eleutherine bulbosa	18.0108333
542	Iridaceae	Eleutherine bulbosa	22.160501
543	Iridaceae	Eleutherine bulbosa	19.196725
544	Iridaceae	Fosteria oaxacana	17.3422222
545	Iridaceae	Fosteria oaxacana	16.9166667
546	Iridaceae	Fosteria oaxacana	17
547	Iridaceae	Iris missouriensis	27.7237778
548	Iridaceae	Iris missouriensis	25.2280611
549	Iridaceae	Nemastylis tenuis	21.2933333
550	Iridaceae	Nemastylis tenuis	23.3947222
551	Iridaceae	Nemastylis tenuis	18.8005556
552	Iridaceae	Nemastylis tenuis	18.9519444
553	Iridaceae	Nemastylis tenuis	20.1447222
554	Iridaceae	Nemastylis tenuis	19.1683306
555	Iridaceae	Nemastylis tenuis	23.8666667
556	Iridaceae	Nemastylis tenuis	20.1596611
557	Iridaceae	Nemastylis tenuis	26.1676
558	Iridaceae	Nemastylis tenuis	23.4556667
559	Iridaceae	Nemastylis tenuis	26.3719444
560	Iridaceae	Nemastylis tenuis spp. pringlei	30.1402361
561	Iridaceae	Nemastylis tenuis spp. pringlei	19.3297194
562	Iridaceae	Nemastylis tenuis spp. pringlei	20.7236194
563	Iridaceae	Nemastylis tenuis var. nana	19.6257111
564	Iridaceae	Nemastylis tenuis	23.4033333
565	Iridaceae	Nemastylis tenuis spp. coerulescens	18.9666667
566	Iridaceae	Nemastylis tenuis var. nana	20.1444444
567	Iridaceae	Nemastylis tenuis spp. pringlei	23.9833333
568	Iridaceae	Nemastylis tenuis var. tenuis	28.4275
569	Iridaceae	Nemastylis tenuis	27.9722222
570	Iridaceae	Colima convoluta	19.310813
571	Iridaceae	Colima convoluta	19.05
572	Iridaceae	Colima tuitensis	22.0397222
573	Iridaceae	Orthrosanthus exsertus	19.4608333
574	Iridaceae	Orthrosanthus exsertus	19.4325
575	Iridaceae	Orthrosanthus exsertus	19.675
576	Iridaceae	Orthrosanthus exsertus	19.2888889
577	Iridaceae	Orthrosanthus exsertus	23.0461111

578	Iridaceae	Orthrosanthus exsertus	20.7283333
579	Iridaceae	Orthrosanthus exsertus	21.2063889
580	Iridaceae	Orthrosanthus exsertus	17.0283333
581	Iridaceae	Orthrosanthus monadelphus	16.7377778
582	Iridaceae	Orthrosanthus monadelphus	16.1758333
583	Iridaceae	Orthrosanthus monadelphus	19.1608333
584	Iridaceae	Orthrosanthus monadelphus	17.5861111
585	Iridaceae	Orthrosanthus monadelphus	16.1016667
586	Iridaceae	Sessilanthera heliantha	18.24
587	Iridaceae	Sessilanthera heliantha	17.5513889
588	Iridaceae	Sessilanthera latifolia spp. lutea	18.2012278
589	Iridaceae	Sessilanthera citrina	17.701597
590	Iridaceae	Tigridia ehrenbergii	20.1333333
591	Iridaceae	Tigridia ehrenbergii	19.5055556
592	Iridaceae	Tigridia ehrenbergii spp. flaviglandifera	21.0277778
593	Iridaceae	Tigridia ehrenbergii	20.5292583
594	Iridaceae	Tigridia ehrenbergii	20.5661333
595	Iridaceae	Tigridia ehrenbergii spp. ehrenbergii	18.9166667
596	Iridaceae	Tigridia ehrenbergii	19.0833333
597	Iridaceae	Tigridia pavonia	19.8983333
598	Iridaceae	Tigridia pavonia	19.513813
599	Iridaceae	Tigridia pavonia	19.698263
600	Iridaceae	Tigridia pavonia	19.126716
601	Iridaceae	Tigridia pavonia	21.2775
602	Iridaceae	Tigridia pavonia	19.3441667
603	Iridaceae	Tigridia pavonia	17.3625
604	Iridaceae	Tigridia pavonia	18.6513139
605	Iridaceae	Tigridia pavonia	27.3717556
606	Iridaceae	Tigridia pavonia	28.4952778
607	Iridaceae	Tigridia pavonia	25.1833333
608	Iridaceae	Tigridia pavonia	23.3333
609	Iridaceae	Tigridia pavonia	20.215
610	Iridaceae	Tigridia pavonia	20.2116667
611	Iridaceae	Tigridia pavonia	18.8566667
612	Iridaceae	Tigridia pavonia	21.2366667
613	Iridaceae	Tigridia pavonia	20.2566667
614	Iridaceae	Tigridia pavonia	16.7166667
615	Iridaceae	Tigridia alpestris	19.6216667
616	Iridaceae	Tigridia alpestris	20.2275
617	Iridaceae	Tigridia alpestris	18.9066667
618	Iridaceae	Tigridia amatlanensis	17.595444
619	Iridaceae	Tigridia augusta	20.11
620	Iridaceae	Tigridia augusta	22.3036111
621	Iridaceae	Tigridia augusta	19.7816667
622	Iridaceae	Tigridia augusta	21.0166667

623	Iridaceae	Tigridia bicolor	17.7441667
624	Iridaceae	Tigridia bicolor	17.8775
625	Iridaceae	Tigridia catarinensis	22.5166667
626	Iridaceae	Tigridia chiapensis	16.73
627	Iridaceae	Tigridia chrysantha	20.45
628	Iridaceae	Tigridia dugesii	20.6775
629	Iridaceae	Tigridia durangensis	23.7783333
630	Iridaceae	Tigridia durangensis	23.4775
631	Iridaceae	Tigridia estelae	24.8033333
632	Iridaceae	Tigridia flammea	19.128087
633	Iridaceae	Tigridia galanthoides	18.8497222
634	Iridaceae	Tigridia galanthoides	16.2517389
635	Iridaceae	Tigridia galanthoides	17.6516667
636	Iridaceae	Tigridia gracielae	19.0298611
637	Iridaceae	Tigridia hallbergii	16.7608333
638	Iridaceae	Tigridia hallbergii	21.2875
639	Iridaceae	Tigridia hallbergii	17.265
640	Iridaceae	Tigridia hintonii	17.901582
641	Iridaceae	Tigridia huajuapanensis	17.2261111
642	Iridaceae	Tigridia huajuapanensis	17.9055556
643	Iridaceae	Tigridia huajuapanensis	17.0258333
644	Iridaceae	Tigridia huajuapanensis	17.82
645	Iridaceae	Tigridia illecebrosa	18.03333
646	Iridaceae	Tigridia inusitata	17.2667
647	Iridaceae	Tigridia inusitata	17.4114
648	Iridaceae	Tigridia immaculata	16.637306
649	Iridaceae	Tigridia immaculata	17.169718
650	Iridaceae	Tigridia mariaetrinitatis	17.0336111
651	Iridaceae	Tigridia martinezii	20.2138889
652	Iridaceae	Tigridia martinezii	20.135
653	Iridaceae	Tigridia matudae	19.1583333
654	Iridaceae	Tigridia matudae	19.0333333
655	Iridaceae	Tigridia meleagris	19.5380556
656	Iridaceae	Tigridia meleagris	19.6941667
657	Iridaceae	Tigridia meleagris	18.98
658	Iridaceae	Tigridia meleagris	19.31
659	Iridaceae	Tigridia meleagris	18.7033333
660	Iridaceae	Tigridia mexicana	19.1933333
661	Iridaceae	Tigridia mexicana	20.5608333
662	Iridaceae	Tigridia mexicana	24.3177778
663	Iridaceae	Tigridia mexicana spp. lilacina	19.4211111
664	Iridaceae	Tigridia mexicana spp. passiflora	21.3333333
665	Iridaceae	Tigridia mexicana spp. passiflora	20.7833333
666	Iridaceae	Tigridia molseediana	17.0977278
667	Iridaceae	Tigridia multiflora	19.0133333

668	Iridaceae	Tigridia multiflora	23.3961111
669	Iridaceae	Tigridia multiflora	19.9608333
670	Iridaceae	Tigridia multiflora	19.73
671	Iridaceae	Tigridia multiflora	20.1941667
672	Iridaceae	Tigridia multiflora	20.9441667
673	Iridaceae	Tigridia multiflora	19.7669444
674	Iridaceae	Tigridia multiflora	19.32
675	Iridaceae	Tigridia multiflora	23.0833333
676	Iridaceae	Tigridia multiflora	23.9602222
677	Iridaceae	Tigridia multiflora	21.7694444
678	Iridaceae	Tigridia multiflora	18.9366667
679	Iridaceae	Tigridia orthantha	17.4640611
680	Iridaceae	Tigridia potosina	22.0297222
681	Iridaceae	Tigridia pugana	20.3697
682	Iridaceae	Tigridia pugana	19.5852778
683	Iridaceae	Tigridia pulchella	19.666667
684	Iridaceae	Tigridia pulchella	19.485161
685	Iridaceae	Tigridia purpusii	18.522351
686	Iridaceae	Tigridia rzedowskiana	21.119294
687	Iridaceae	Tigridia seleriana	17.4430556
688	Iridaceae	Tigridia suarezii	20.05
689	Iridaceae	Tigridia suarezii	20
690	Iridaceae	Tigridia suarezii	20.3533333
691	Iridaceae	Tigridia tepoxtlana	19.0130556
692	Iridaceae	Tigridia tepoxtlana	18.9866667
693	Iridaceae	Tigridia vanhouttei	19.5275
694	Iridaceae	Tigridia vanhouttei	19.3275
695	Iridaceae	Tigridia vanhouttei	20.9275
696	Iridaceae	Tigridia vanhouttei	19.9108333
697	Iridaceae	Tigridia vanhouttei spp. vanhouttei	19.7811111
698	Iridaceae	Tigridia vanhouttei spp. vanhouttei	19.7797222
699	Iridaceae	Tigridia vanhouttei spp. roldanii	19.0733333
700	Iridaceae	Tigridia vanhouttei spp. vanhouttei	20.42
701	Iridaceae	Tigridia vanhouttei spp. vanhouttei	17.51
702	Iridaceae	Tigridia vanhouttei spp. vanhouttei	19.5608333
703	Iridaceae	Tigridia venusta	19.6333333
704	Iridaceae	Tigridia venusta	19.6016667
705	Iridaceae	Rigidella inusitata	17.489881
706	Iridaceae	Trimezia steyermarkii	19.205373
707	Iridaceae	Trimezia steyermarkii	18.235
708	Iridaceae	Trimezia steyermarkii	17.1275
709	Iridaceae	Trimezia steyermarkii	17.4583333
710	Iridaceae	Trimezia steyermarkii	16.9083333
713	Orchidaceae	Aulosepalum hemichrea	15.189
714	Orchidaceae	Aulosepalum hemichrea	16.21

715	Orchidaceae	Aulosepalum hemichrea	17.093
716	Orchidaceae	Aulosepalum pyramidale	18.6333333
717	Orchidaceae	Aulosepalum pyramidale	19.5158333
718	Orchidaceae	Brachystele chiangii	25.1333333
719	Orchidaceae	Brachystele chiangii	25.0984167
720	Orchidaceae	Brachystele chiangii	24.1349167
721	Orchidaceae	Brachystele polyantha	24.3186111
722	Orchidaceae	Brachystele polyantha	24.578125
723	Orchidaceae	Brachystele polyantha	25.6038972
724	Orchidaceae	Brachystele polyantha	26.5027778
725	Orchidaceae	Brachystele polyantha	19.3133333
726	Orchidaceae	Brachystele polyantha	19.422987
727	Orchidaceae	Brachystele polyantha	17.5108333
728	Orchidaceae	Brachystele polyantha	18.3108333
729	Orchidaceae	Corallorhiza bulbosa	23.8030556
730	Orchidaceae	Corallorhiza bulbosa	20.0633333
731	Orchidaceae	Corallorhiza bulbosa	19.68
732	Orchidaceae	Corallorhiza bulbosa	21.2875
733	Orchidaceae	Corallorhiza bulbosa	20.2266667
734	Orchidaceae	Corallorhiza ehrenbergii	17.2683333
735	Orchidaceae	Corallorhiza ehrenbergii	21.1833333
736	Orchidaceae	Corallorhiza ehrenbergii	19.4783333
737	Orchidaceae	Corallorhiza ehrenbergii	20.1833333
738	Orchidaceae	Corallorhiza macrantha	16.840715
739	Orchidaceae	Corallorhiza maculata var. mexicana	19.6422222
740	Orchidaceae	Corallorhiza maculata var. mexicana	15.4305556
741	Orchidaceae	Corallorhiza maculata var. mexicana	23.804075
742	Orchidaceae	Corallorhiza maculata var. mexicana	16.1
743	Orchidaceae	Corallorhiza odontorhiza var. pringlei	28.3990083
744	Orchidaceae	Corallorhiza odontorhiza var. pringlei	19.4697222
745	Orchidaceae	Corallorhiza odontorhiza var. pringlei	16.58
746	Orchidaceae	Corallorhiza odontorhiza var. pringlei	16.85
747	Orchidaceae	Corallorhiza striata var. Involuta	27.1333333
748	Orchidaceae	Corallorhiza striata var. Involuta	24.5944444
749	Orchidaceae	Corallorhiza striata var. Involuta	17.7608333
750	Orchidaceae	Corallorhiza striata var. vreelandii	23.835
751	Orchidaceae	Corallorhiza williamsii	18.986407
752	Orchidaceae	Corallorhiza wisteriana	16.4616667
753	Orchidaceae	Corallorhiza wisteriana	16.5080556
754	Orchidaceae	Corallorhiza wisteriana	25.6004389
755	Orchidaceae	Corallorhiza wisteriana	25.1866667
756	Orchidaceae	Cyclopogon pringlei	18.77
757	Orchidaceae	Cyclopogon saccatus	18.813244
758	Orchidaceae	Cyclopogon saccatus	19.494953
759	Orchidaceae	Cyclopogon saccatus	19.6183333

760	Orchidaceae	Cypripedium dickinsonianum	16.21648
761	Orchidaceae	Cypripedium irapeanum	19.2775
762	Orchidaceae	Cypripedium irapeanum	19.443298
763	Orchidaceae	Cypripedium irapeanum	19.3441667
764	Orchidaceae	Cypripedium irapeanum	18.8858333
765	Orchidaceae	Cypripedium irapeanum	17.8941667
766	Orchidaceae	Cypripedium irapeanum	17.6275
767	Orchidaceae	Cypripedium irapeanum	16.9168833
768	Orchidaceae	Cypripedium irapeanum	16.2
769	Orchidaceae	Cypripedium irapeanum	18.9091667
770	Orchidaceae	Cypripedium irapeanum	19.2777778
771	Orchidaceae	Cypripedium irapeanum	20.43
772	Orchidaceae	Cypripedium irapeanum	18.85
773	Orchidaceae	Cypripedium molle	18.0441667
774	Orchidaceae	Cypripedium molle	17.9108333
775	Orchidaceae	Cypripedium molle	17.3608333
776	Orchidaceae	Cypripedium molle	17.7275
777	Orchidaceae	Cypripedium molle	17.5441667
778	Orchidaceae	Cypripedium molle	18.8497222
779	Orchidaceae	Cypripedium molle	17.8275
780	Orchidaceae	Cypripedium molle	16.9844389
781	Orchidaceae	Cypripedium molle	17.585
782	Orchidaceae	Deiregyne alinae	19.675
783	Orchidaceae	Deiregyne alinae	19.8716667
784	Orchidaceae	Deiregyne cochleata	20.528847
785	Orchidaceae	Deiregyne densiflora	18.05
786	Orchidaceae	Deiregyne densiflora	17.11389
787	Orchidaceae	Deiregyne densiflora	18.983
788	Orchidaceae	Deiregyne densiflora	18.25
789	Orchidaceae	Deiregyne densiflora	19.5875
790	Orchidaceae	Deiregyne densiflora	20.125
791	Orchidaceae	Deiregyne densiflora	21.5
792	Orchidaceae	Deiregyne densiflora	25.33
793	Orchidaceae	Deiregyne nelsonii subsp. pulchra	21.2216667
794	Orchidaceae	Deiregyne nelsonii	17.35
795	Orchidaceae	Deiregyne ramentacea	23.0461111
796	Orchidaceae	Deiregyne ramirezii	20.112909
797	Orchidaceae	Deiregyne rhombilabia	18.6108333
798	Orchidaceae	Deiregyne rhombilabia	18.9719444
799	Orchidaceae	Deiregyne sheviakiana	16.726062
800	Orchidaceae	Deiregyne tenorioi	18.05
801	Orchidaceae	Deiregyne tenuiflora	18.875
802	Orchidaceae	Deiregyne tenuiflora	18.7833333
803	Orchidaceae	Dichromanthus aurantiacus	20.3441667
804	Orchidaceae	Dichromanthus aurantiacus	19.5875

805	Orchidaceae	Dichromanthus aurantiacus	16.6961111
806	Orchidaceae	Dichromanthus cinnabarinus	22.6608333
807	Orchidaceae	Dichromanthus cinnabarinus	19.5108333
808	Orchidaceae	Dichromanthus cinnabarinus	18.8463889
809	Orchidaceae	Dichromanthus cinnabarinus	19.342985
810	Orchidaceae	Dichromanthus cinnabarinus	25.4108333
811	Orchidaceae	Dichromanthus cinnabarinus	17.8000361
812	Orchidaceae	Dichromanthus cinnabarinus	17.5669167
813	Orchidaceae	Dichromanthus cinnabarinus	23.9291667
814	Orchidaceae	Dichromanthus cinnabarinus	25.3833333
815	Orchidaceae	Dichromanthus cinnabarinus	19.8166667
816	Orchidaceae	Dichromanthus cinnabarinus	19.3136111
817	Orchidaceae	Dichromanthus cinnabarinus	16.7566667
818	Orchidaceae	Dichromanthus cinnabarinus	19.0166667
819	Orchidaceae	Dichromanthus cinnabarinus	18.0608333
820	Orchidaceae	Dichromanthus cinnabarinus	25.0984167
821	Orchidaceae	Dichromanthus michuacanus	17.5847
822	Orchidaceae	Dichromanthus michuacanus	16.31556
823	Orchidaceae	Dichromanthus michuacanus	18.75
824	Orchidaceae	Dichromanthus michuacanus	18.967
825	Orchidaceae	Dichromanthus michuacanus	18.8966667
826	Orchidaceae	Dichromanthus michuacanus	18.9
827	Orchidaceae	Dichromanthus michuacanus	19.588
828	Orchidaceae	Dichromanthus michuacanus	19.02639
829	Orchidaceae	Dichromanthus michuacanus	19.492
830	Orchidaceae	Dichromanthus michuacanus	20.4933333
831	Orchidaceae	Dichromanthus michuacanus	20.705
832	Orchidaceae	Dichromanthus michuacanus	23.55
833	Orchidaceae	Dichromanthus michuacanus	23.45278
834	Orchidaceae	Dichromanthus michuacanus	27.972
835	Orchidaceae	Dichromanthus michuacanus	28.008
836	Orchidaceae	Dichromanthus michuacanus	28.40306
837	Orchidaceae	Funkiella laxispica	17.050276
838	Orchidaceae	Funkiella parasitica	16.5
839	Orchidaceae	Funkiella parasitica	23.966667
840	Orchidaceae	Funkiella parasitica	21.93
841	Orchidaceae	Funkiella rubrocallosa	27.4325
842	Orchidaceae	Funkiella rubrocallosa	27.0683333
843	Orchidaceae	Funkiella stolonifera	15.115
844	Orchidaceae	Funkiella valerioi	19.33
845	Orchidaceae	Galeoglossum cactorum	17.113069
846	Orchidaceae	Galeoglossum tubulosum	19.03
847	Orchidaceae	Galeoglossum tubulosum	19.1
848	Orchidaceae	Galeottiella sarcoglossa	20.8063889
849	Orchidaceae	Galeottiella sarcoglossa	16.14

850	Orchidaceae	Galeottiella sarcoglossa	19.13333
851	Orchidaceae	Galeottiella sarcoglossa	18.76666
852	Orchidaceae	Habenaria agapitae	16.7353
853	Orchidaceae	Habenaria clypeata	20.5275
854	Orchidaceae	Habenaria clypeata	19.3441667
855	Orchidaceae	Habenaria clypeata	19.8
856	Orchidaceae	Habenaria clypeata	19.543641
857	Orchidaceae	Habenaria clypeata	18.8497222
858	Orchidaceae	Habenaria clypeata	20.8108333
859	Orchidaceae	Habenaria clypeata	19.5383333
860	Orchidaceae	Habenaria clypeata	21.3608333
861	Orchidaceae	Habenaria clypeata	19.32
862	Orchidaceae	Habenaria clypeata	19.1638889
863	Orchidaceae	Habenaria clypeata	19.3425
864	Orchidaceae	Habenaria clypeata	28.4194444
865	Orchidaceae	Habenaria clypeata	17.2346889
866	Orchidaceae	Habenaria clypeata	28.4952778
867	Orchidaceae	Habenaria clypeata	23.8436111
868	Orchidaceae	Habenaria clypeata	19.850725
869	Orchidaceae	Habenaria clypeata	22.3557556
870	Orchidaceae	Habenaria clypeata	20.0398556
871	Orchidaceae	Habenaria clypeata	19.0166667
872	Orchidaceae	Habenaria clypeata	23.471525
873	Orchidaceae	Habenaria clypeata	25.8241667
874	Orchidaceae	Habenaria filifera	19.8775
875	Orchidaceae	Habenaria filifera	17.7713889
876	Orchidaceae	Habenaria filifera	20.11
877	Orchidaceae	Habenaria filifera	20.3805556
878	Orchidaceae	Habenaria ixtlanensis	17.7941667
879	Orchidaceae	Habenaria ixtlanensis	17.11
880	Orchidaceae	Habenaria novemfida	16.99
881	Orchidaceae	Habenaria novemfida	19.45
882	Orchidaceae	Habenaria novemfida	19.2
883	Orchidaceae	Habenaria novemfida	17.81666
884	Orchidaceae	Habenaria novemfida	19.7
885	Orchidaceae	Habenaria novemfida	19.6180556
886	Orchidaceae	Habenaria novemfida	19.5
887	Orchidaceae	Habenaria novemfida	18.91666
888	Orchidaceae	Habenaria novemfida	21.43333
889	Orchidaceae	Habenaria strictissima	16.73
890	Orchidaceae	Habenaria strictissima	19.3
891	Orchidaceae	Habenaria strictissima	21.39
892	Orchidaceae	Habenaria strictissima	19.6180556
893	Orchidaceae	Habenaria strictissima	18.73
894	Orchidaceae	Habenaria virens	16.68

895	Orchidaceae	Habenaria virens	15.91666
896	Orchidaceae	Hexalectris brevicaulis	17.8
897	Orchidaceae	Hexalectris brevicaulis	18.467
898	Orchidaceae	Hexalectris brevicaulis	19.603
899	Orchidaceae	Hexalectris brevicaulis	20.867
900	Orchidaceae	Hexalectris grandiflora	17.447
901	Orchidaceae	Hexalectris grandiflora	18.94
902	Orchidaceae	Hexalectris grandiflora	18.65
903	Orchidaceae	Hexalectris grandiflora	19.933
904	Orchidaceae	Hexalectris grandiflora	19.463
905	Orchidaceae	Hexalectris grandiflora	23.5
906	Orchidaceae	Hexalectris grandiflora	23.97
907	Orchidaceae	Hexalectris grandiflora	25.3803056
908	Orchidaceae	Hexalectris grandiflora	27.1
909	Orchidaceae	Hexalectris grandiflora	28.28333
910	Orchidaceae	Hexalectris nitida	24.683
911	Orchidaceae	Hexalectris parviflora	21.351
912	Orchidaceae	Hexalectris revoluta	24.695156
913	Orchidaceae	Hexalectris warnockii	29.50886
914	Orchidaceae	Liparis cordiformis	18.937
915	Orchidaceae	Liparis cordiformis	19.418
916	Orchidaceae	Liparis cordiformis	19.478
917	Orchidaceae	Liparis draculoides	17.3
918	Orchidaceae	Liparis draculoides	17.317
919	Orchidaceae	Liparis draculoides	19.411
920	Orchidaceae	Liparis draculoides	19.383
921	Orchidaceae	Liparis draculoides	20.215
922	Orchidaceae	Liparis greenwoodiana	18.95
923	Orchidaceae	Liparis greenwoodiana	18.863
924	Orchidaceae	Liparis greenwoodiana	18.983
925	Orchidaceae	Liparis madrensis	23.9166667
926	Orchidaceae	Liparis vexillifera	16.272
927	Orchidaceae	Liparis vexillifera	16.656
928	Orchidaceae	Liparis vexillifera	17.23
929	Orchidaceae	Liparis vexillifera	18.905
930	Orchidaceae	Liparis vexillifera	18.465
931	Orchidaceae	Liparis vexillifera	19.6
932	Orchidaceae	Liparis vexillifera	19.802
933	Orchidaceae	Liparis vexillifera	20.501
934	Orchidaceae	Liparis vexillifera	21.333
935	Orchidaceae	Liparis vexillifera	21.11
936	Orchidaceae	Liparis vexillifera	22.5
937	Orchidaceae	Liparis vexillifera	23.247
938	Orchidaceae	Liparis vexillifera	27.748
939	Orchidaceae	Malaxis carnosa	16.03333

940	Orchidaceae	Malaxis carnosa	16.308
941	Orchidaceae	Malaxis carnosa	18.15
942	Orchidaceae	Malaxis carnosa	18.453
943	Orchidaceae	Malaxis carnosa	19.7
944	Orchidaceae	Malaxis carnosa	19.3
945	Orchidaceae	Malaxis carnosa	24.167
946	Orchidaceae	Malaxis carnosa	24.51667
947	Orchidaceae	Malaxis greenwoodiana	16.75
948	Orchidaceae	Malaxis greenwoodiana	17.2
949	Orchidaceae	Malaxis hagsateri	17.525012
950	Orchidaceae	Malaxis hagsateri	17.070411
951	Orchidaceae	Malaxis javesiae	16.75
952	Orchidaceae	Malaxis javesiae	17.833
953	Orchidaceae	Malaxis myurus	16.1688889
954	Orchidaceae	Malaxis myurus	18.983
955	Orchidaceae	Malaxis myurus	19.34
956	Orchidaceae	Malaxis myurus	19.669
957	Orchidaceae	Malaxis myurus	20.367
958	Orchidaceae	Malaxis myurus	20.343
959	Orchidaceae	Malaxis myurus	22.525
960	Orchidaceae	Malaxis myurus	22.32
961	Orchidaceae	Malaxis myurus	23.526
962	Orchidaceae	Malaxis myurus	26.47
963	Orchidaceae	Malaxis myurus	28.413
964	Orchidaceae	Malaxis novogaliciana	21.072
965	Orchidaceae	Malaxis novogaliciana	22.135
966	Orchidaceae	Malaxis novogaliciana	23.45
967	Orchidaceae	Malaxis rosilloi	16.248
968	Orchidaceae	Malaxis rosilloi	18.967
969	Orchidaceae	Malaxis urbana	18.4527778
970	Orchidaceae	Mesadenus lucayanus	21.6344444
971	Orchidaceae	Mesadenus tenuissimus	17.4517
972	Orchidaceae	Funkiella minutiflora	15.427
973	Orchidaceae	Funkiella minutiflora	19.03
974	Orchidaceae	Funkiella minutiflora	19.08861
975	Orchidaceae	Ponthieva mexicana	16.75
976	Orchidaceae	Ponthieva mexicana	16.66
977	Orchidaceae	Ponthieva mexicana	17.18
978	Orchidaceae	Ponthieva mexicana	19.667
979	Orchidaceae	Ponthieva mexicana	19.69
980	Orchidaceae	Ponthieva mexicana	20.194
981	Orchidaceae	Ponthieva mexicana	21.292
982	Orchidaceae	Ponthieva mexicana	23.9786111
983	Orchidaceae	Pelexia obliqua	18.58333
984	Orchidaceae	Physogyne garayana	18.944351

985	Orchidaceae	Ponthieva brittoniae	18.81417
986	Orchidaceae	Ponthieva schaffneri	16.17
987	Orchidaceae	Ponthieva schaffneri	16.98
988	Orchidaceae	Ponthieva schaffneri	17.42
989	Orchidaceae	Ponthieva schaffneri	19.454414
990	Orchidaceae	Ponthieva schaffneri	19.62
991	Orchidaceae	Ponthieva schaffneri	19.218
992	Orchidaceae	Ponthieva schaffneri	19.48
993	Orchidaceae	Ponthieva schaffneri	19.468
994	Orchidaceae	Ponthieva schaffneri	19.73
995	Orchidaceae	Ponthieva schaffneri	21.155
996	Orchidaceae	Ponthieva schaffneri	21.285
997	Orchidaceae	Ponthieva schaffneri	23.9916667
998	Orchidaceae	Ponthieva schaffneri	23.979
999	Orchidaceae	Ponthieva schaffneri	27.748
1000	Orchidaceae	Pteroglossa roseoalba	15.97611
1001	Orchidaceae	Pteroglossa roseoalba	15.69
1002	Orchidaceae	Sacoila lanceolata	15.96139
1003	Orchidaceae	Sacoila lanceolata	16.414
1004	Orchidaceae	Sacoila lanceolata	17.29444
1005	Orchidaceae	Sacoila lanceolata	17.835
1006	Orchidaceae	Sacoila lanceolata	17.77
1007	Orchidaceae	Sacoila lanceolata	18.61
1008	Orchidaceae	Sacoila lanceolata	18.967
1009	Orchidaceae	Sacoila lanceolata	18.10333
1010	Orchidaceae	Sacoila lanceolata	19.917
1011	Orchidaceae	Sacoila lanceolata	19.938
1012	Orchidaceae	Sacoila lanceolata	20.85
1013	Orchidaceae	Sacoila lanceolata	21.7
1014	Orchidaceae	Sacoila lanceolata	21.05
1015	Orchidaceae	Sacoila lanceolata	21.222
1016	Orchidaceae	Sacoila lanceolata	23.5
1017	Orchidaceae	Sacoila lanceolata	26.812
1018	Orchidaceae	Sacoila lanceolata	28.383
1019	Orchidaceae	Sarcoglottis assurgens	16.344
1020	Orchidaceae	Sarcoglottis assurgens	18.82
1021	Orchidaceae	Sarcoglottis assurgens	18.51583
1022	Orchidaceae	Sarcoglottis assurgens	19.562
1023	Orchidaceae	Sarcoglottis assurgens	22.13333
1024	Orchidaceae	Sarcoglottis cerina	16.617
1025	Orchidaceae	Sarcoglottis cerina	19.653
1026	Orchidaceae	Sarcoglottis corymbosa	16.7
1027	Orchidaceae	Sarcoglottis corymbosa	16.07
1028	Orchidaceae	Sarcoglottis corymbosa	20.5
1029	Orchidaceae	Sarcoglottis corymbosa	28.39444

1030	Orchidaceae	Sarcoglottis schaffneri	16.717
1031	Orchidaceae	Sarcoglottis schaffneri	17.31667
1032	Orchidaceae	Sarcoglottis schaffneri	18.317
1033	Orchidaceae	Sarcoglottis schaffneri	18.97
1034	Orchidaceae	Sarcoglottis schaffneri	18.56667
1035	Orchidaceae	Sarcoglottis schaffneri	19.25
1036	Orchidaceae	Sarcoglottis schaffneri	19.31
1037	Orchidaceae	Sarcoglottis schaffneri	19.02639
1038	Orchidaceae	Sarcoglottis schaffneri	20.607
1039	Orchidaceae	Sarcoglottis schaffneri	21.242
1040	Orchidaceae	Sarcoglottis schaffneri	22.7
1041	Orchidaceae	Sarcoglottis schaffneri	23.15
1042	Orchidaceae	Sarcoglottis schaffneri	23.9786111
1043	Orchidaceae	Sarcoglottis schaffneri	28.1
1044	Orchidaceae	Sarcoglottis scintillans	16.2667
1045	Orchidaceae	Schiedeella affinis	17.62
1046	Orchidaceae	Schiedeella affinis	18.983
1047	Orchidaceae	Schiedeella affinis	19.2083
1048	Orchidaceae	Schiedeella affinis	20.371
1049	Orchidaceae	Schiedeella affinis	20.483
1050	Orchidaceae	Schiedeella affinis	27.58333
1051	Orchidaceae	Schiedeella albovaginata	18.983
1052	Orchidaceae	Schiedeella chartacea	19.7
1053	Orchidaceae	Schiedeella crenulata	20.1833333
1054	Orchidaceae	Schiedeella crenulata	21.151
1055	Orchidaceae	Schiedeella diaphana	16.93
1056	Orchidaceae	Schiedeella diaphana	18.13333
1057	Orchidaceae	Schiedeella eriophora	16.1688889
1058	Orchidaceae	Schiedeella eriophora	17.56
1059	Orchidaceae	Schiedeella eriophora	19.46
1060	Orchidaceae	Schiedeella eriophora	19.02
1061	Orchidaceae	Schiedeella eriophora	19.018
1062	Orchidaceae	Schiedeella eriophora	19.13333
1063	Orchidaceae	Schiedeella eriophora	23.533
1064	Orchidaceae	Schiedeella eriophora	23.9786111
1065	Orchidaceae	Schiedeella eriophora	28.36667
1066	Orchidaceae	Schiedeella eriophora	28.18333
1067	Orchidaceae	Schiedeella falcata	16.78469
1068	Orchidaceae	Schiedeella falcata	25.9
1069	Orchidaceae	Schiedeella falcata	26.2
1070	Orchidaceae	Schiedeella garayana	19.133
1071	Orchidaceae	Schiedeella garayana	23.046
1072	Orchidaceae	Schiedeella llaveana	19.067
1073	Orchidaceae	Schiedeella nonantzin	20.301679
1074	Orchidaceae	Schiedeella pandurata	25.9

1075	Orchidaceae	Schiedeella pseudopyramidalis	16.98333
1076	Orchidaceae	Schiedeella pseudopyramidalis	17.18
1077	Orchidaceae	Schiedella sparsiflora	18.92
1078	Orchidaceae	Schiedella sparsiflora	18.917
1079	Orchidaceae	Schiedella sparsiflora	18.6
1080	Orchidaceae	Schiedeella trilineata	16.15
1081	Orchidaceae	Schiedeella velata	29.922238
1082	Orchidaceae	Schiedeella violacea	15.86667
1083	Orchidaceae	Schiedeella violacea	15.946
1084	Orchidaceae	Sotoa confusa	17.56167
1085	Orchidaceae	Sotoa confusa	18.23333
1086	Orchidaceae	Sotoa confusa	19.832
1087	Orchidaceae	Sotoa confusa	19.35
1088	Orchidaceae	Sotoa confusa	22.72444
1089	Orchidaceae	Beloglottis costaricensis	15.98028
1090	Orchidaceae	Beloglottis costaricensis	16.5
1091	Orchidaceae	Beloglottis costaricensis	16.087
1092	Orchidaceae	Beloglottis costaricensis	18.42806
1093	Orchidaceae	Beloglottis costaricensis	20.1
1094	Orchidaceae	Beloglottis costaricensis	21.25
1095	Orchidaceae	Beloglottis costaricensis	22.5
1096	Orchidaceae	Stenorrhynchos sulphureum	18.962
1097	Orchidaceae	Wullschlaegelia aphylla	16.95
1098	Orchidaceae	Wullschlaegelia aphylla	17.233
1100	Amaryllidaceae	Zephyranthes verecunda	18.698
1101	Amaryllidaceae	Zephyranthes verecunda	19.618
1102	Amaryllidaceae	Zephyranthes verecunda	19.207
1103	Amaryllidaceae	Zephyranthes verecunda	19.277
1104	Amaryllidaceae	Zephyranthes verecunda	19.322
1105	Amaryllidaceae	Zephyranthes verecunda	20.017
1106	Liliaceae	Calochortus ambiguus	31.30528
1107	Liliaceae	Calochortus balsensis	17.23
1108	Liliaceae	Calochortus balsensis	17.28
1109	Liliaceae	Calochortus barbatus	16.468
1110	Liliaceae	Calochortus barbatus	19.95
1111	Liliaceae	Calochortus barbatus	18.204
1112	Liliaceae	Calochortus barbatus	18.905
1113	Liliaceae	Calochortus barbatus	18.7
1114	Liliaceae	Calochortus barbatus	19.992
1115	Liliaceae	Calochortus barbatus	19.38
1116	Liliaceae	Calochortus barbatus	19.92
1117	Liliaceae	Calochortus barbatus	19.561
1118	Liliaceae	Calochortus barbatus	20.508
1119	Liliaceae	Calochortus barbatus	20.805
1120	Liliaceae	Calochortus barbatus	20.783

1121	Liliaceae	Calochortus barbatus	21.35
1122	Liliaceae	Calochortus barbatus	21.658
1123	Liliaceae	Calochortus barbatus	23.422
1124	Liliaceae	Calochortus barbatus	23.437
1125	Liliaceae	Calochortus barbatus	27.19
1126	Liliaceae	Calochortus barbatus	29.687
1127	Liliaceae	Calochortus cernuus	18.982
1128	Liliaceae	Calochortus cernuus	19.853
1129	Liliaceae	Calochortus cernuus	20.882
1130	Liliaceae	Calochortus concolor	32
1131	Liliaceae	Calochortus exilis	18.95
1132	Liliaceae	Calochortus exilis	20.297
1133	Liliaceae	Calochortus exilis	22.913
1134	Liliaceae	Calochortus exilis	23
1135	Liliaceae	Calochortus exilis	28.6635
1136	Liliaceae	Calochortus foliosus	19.51
1137	Liliaceae	Calochortus fuscus	18.85
1138	Liliaceae	Calochortus fuscus	18.987
1139	Liliaceae	Calochortus fuscus	19.818
1140	Liliaceae	Calochortus fuscus	20.55
1141	Liliaceae	Calochortus fuscus	23.7
1142	Liliaceae	Calochortus fuscus	25.35
1143	Liliaceae	Calochortus fuscus	28.4448333
1144	Liliaceae	Calochortus ghiesbreghtii	16.567
1145	Liliaceae	Calochortus ghiesbreghtii	21.03
1146	Liliaceae	Calochortus ghiesbreghtii	23
1147	Liliaceae	Calochortus hartwegii	20.72
1148	Liliaceae	Calochortus hartwegii	21.333
1149	Liliaceae	Calochortus hartwegii	21.35
1150	Liliaceae	Calochortus hartwegii	21.962
1151	Liliaceae	Calochortus marcellae	23.9786111
1152	Liliaceae	Calochortus marcellae	24.8311111
1153	Liliaceae	Calochortus marcellae	24
1154	Liliaceae	Calochortus marcellae	25.429
1155	Liliaceae	Calochortus mendozae	22.1916667
1156	Liliaceae	Calochortus hintonii	18.972
1157	Liliaceae	Calochortus hintonii	20.55
1158	Liliaceae	Calochortus nigrescens	23
1159	Liliaceae	Calochortus pringlei	18.987
1160	Liliaceae	Calochortus pringlei	18.648
1161	Liliaceae	Calochortus pringlei	19.05
1162	Liliaceae	Calochortus purpureus	18.937
1163	Liliaceae	Calochortus purpureus	19.678
1164	Liliaceae	Calochortus purpureus	19.588
1165	Liliaceae	Calochortus purpureus	19.858

1166	Liliaceae	Calochortus purpureus	20.283
1167	Liliaceae	Calochortus purpureus	23.145
1168	Liliaceae	Calochortus purpureus	23.416
1169	Liliaceae	Calochortus spatulatus	17.6402778
1170	Liliaceae	Calochortus spatulatus	18.68
1171	Liliaceae	Calochortus spatulatus	19.913
1172	Liliaceae	Calochortus spatulatus	19.04
1173	Liliaceae	Calochortus spatulatus	21.58
1174	Liliaceae	Calochortus spatulatus	21.05
1175	Liliaceae	Calochortus spatulatus	27.3
1176	Liliaceae	Calochortus spatulatus	28.20833
1177	Liliaceae	Calochortus splendens	30.97
1178	Liliaceae	Calochortus splendens	31.5
1179	Liliaceae	Calochortus venustulus	19.03
1180	Liliaceae	Calochortus venustulus	20.525
1181	Liliaceae	Calochortus venustulus	21.383
1182	Liliaceae	Calochortus venustulus	23.881694
1183	Liliaceae	Calochortus venustulus	26.57
1184	Liliaceae	Calochortus venustulus	27.323
1185	Liliaceae	Calochortus weedii	30
1186	Liliaceae	Calochortus weedii	31.083
1187	Liliaceae	Calochortus weedii	32.433
1193	Liliaceae	Lilium parryi	30.95
1194	Marantaceae	Maranta gibba	17.9333333
1195	Marantaceae	Maranta gibba	17.0341666
1196	Marantaceae	Maranta gibba	18.2166667
1197	Marantaceae	Maranta gibba	21.2
1198	Marantaceae	Maranta gibba	17.53
1199	Marantaceae	Maranta gibba	18.45
1200	Marantaceae	Maranta gibba	20.6458333
1203	Araceae	Arisaema macrospathum	19.45
1204	Araceae	Arisaema macrospathum	20.96
1205	Araceae	Arisaema macrospathum	19.92
1206	Araceae	Arisaema macrospathum	19.05
1207	Araceae	Arisaema macrospathum	19.65
1208	Araceae	Arisaema macrospathum	18.91
1209	Araceae	Arisaema macrospathum	20.97
1210	Araceae	Arisaema macrospathum	25.36
1211	Araceae	Arisaema macrospathum	21.24
1212	Araceae	Arisaema macrospathum	23.54
1213	Araceae	Arisaema macrospathum	23.04
1214	Araceae	Arisaema macrospathum	18.83
1216	Asparagaceae	Bessera elegans	19.05
1217	Asparagaceae	Bessera elegans	20.6166667
1218	Asparagaceae	Bessera elegans	19.4333333

1219	Asparagaceae	Bessera elegans	18.3
1220	Asparagaceae	Bessera elegans	20.5466667
1221	Asparagaceae	Bessera elegans	19.65
1222	Asparagaceae	Bessera elegans	20.3241667
1223	Asparagaceae	Bessera elegans	20.7166667
1224	Asparagaceae	Bessera elegans	20.3166667
1225	Asparagaceae	Bessera elegans	20.8
1226	Asparagaceae	Bessera elegans	19.2616667
1227	Asparagaceae	Bessera elegans	19.4555556
1228	Asparagaceae	Bessera elegans	18.9666667
1229	Asparagaceae	Bessera elegans	21.3219444
1230	Asparagaceae	Bessera elegans	21.2366667
1231	Asparagaceae	Bessera elegans	16.9763889
1232	Asparagaceae	Bessera tenuiflora	24.25
1233	Asparagaceae	Bessera tenuiflora	23.4327778
1235	Asparagaceae	Dandya balsensis	18.465
1236	Asparagaceae	Dandya balsensis	18.625
1237	Asparagaceae	Dandya balsensis	18.5266667
1238	Asparagaceae	Jaimehintonia gypsophila	24.1033333
1239	Asparagaceae	Milla biflora	18.983
1240	Asparagaceae	Milla biflora	18.983334
1241	Asparagaceae	Milla biflora	31.23889
1242	Asparagaceae	Milla biflora	21.763
1243	Asparagaceae	Milla biflora	21.763334
1244	Asparagaceae	Milla biflora	20.154
1245	Asparagaceae	Milla biflora	20.154167
1246	Asparagaceae	Milla biflora	20.86861
1247	Asparagaceae	Milla biflora	20.869
1248	Asparagaceae	Milla biflora	18.85
1249	Asparagaceae	Milla biflora	18.856667
1250	Asparagaceae	Milla biflora	18.857
1251	Asparagaceae	Milla biflora	20.25
1252	Asparagaceae	Milla biflora	20.25
1253	Asparagaceae	Milla biflora	19.046667
1254	Asparagaceae	Milla biflora	19.047
1255	Asparagaceae	Milla biflora	21.497
1256	Asparagaceae	Milla biflora	21.433
1257	Asparagaceae	Milla biflora	21.433332
1258	Asparagaceae	Milla biflora	19.213055
1259	Asparagaceae	Milla biflora	19.552778
1260	Asparagaceae	Milla biflora	19.798
1261	Asparagaceae	Milla biflora	19.798334
1262	Asparagaceae	Milla biflora	19.833
1263	Asparagaceae	Milla biflora	30.695
1264	Asparagaceae	Milla biflora	21.072

1265	Asparagaceae	Milla biflora	21.0725
1266	Asparagaceae	Milla biflora	18.15
1267	Asparagaceae	Milla biflora	19.745
1268	Asparagaceae	Milla biflora	19.745
1269	Asparagaceae	Milla biflora	19.571945
1270	Asparagaceae	Milla biflora	19.61861
1271	Asparagaceae	Milla biflora	19.635279
1272	Asparagaceae	Milla biflora	18.68
1273	Asparagaceae	Milla biflora	18.77
1274	Asparagaceae	Milla biflora	18.678
1275	Asparagaceae	Milla biflora	18.678333
1276	Asparagaceae	Milla biflora	19.821667
1277	Asparagaceae	Milla biflora	19.822
1278	Asparagaceae	Milla biflora	20.901
1279	Asparagaceae	Milla biflora	20.902
1280	Asparagaceae	Milla biflora	20.718
1281	Asparagaceae	Milla biflora	20.718334
1282	Asparagaceae	Milla biflora	19.81
1283	Asparagaceae	Milla biflora	23.422
1284	Asparagaceae	Milla biflora	23.561
1285	Asparagaceae	Milla biflora	19.33
1286	Asparagaceae	Milla biflora	19.367
1287	Asparagaceae	Milla biflora	19.367
1288	Asparagaceae	Milla biflora	19.367222
1289	Asparagaceae	Milla biflora	19.304167
1290	Asparagaceae	Milla biflora	19.30611
1291	Asparagaceae	Milla biflora	22.434
1292	Asparagaceae	Milla biflora	18.936666
1293	Asparagaceae	Milla biflora	18.937
1294	Asparagaceae	Milla biflora	18.967
1295	Asparagaceae	Milla biflora	18.967
1296	Asparagaceae	Milla biflora	18.967
1297	Asparagaceae	Milla biflora	18.975
1298	Asparagaceae	Milla biflora	28.24
1299	Asparagaceae	Milla biflora	28.135
1300	Asparagaceae	Milla biflora	28.135
1301	Asparagaceae	Milla biflora	21.111666
1302	Asparagaceae	Milla biflora	21.112
1303	Asparagaceae	Milla biflora	23.417
1304	Asparagaceae	Milla biflora	23.45
1305	Asparagaceae	Milla biflora	23.902
1306	Asparagaceae	Milla biflora	23.907
1307	Asparagaceae	Milla biflora	23.91
1308	Asparagaceae	Milla biflora	24.03
1309	Asparagaceae	Milla biflora	24.703

1310	Asparagaceae	Milla biflora	24.703333
1311	Asparagaceae	Milla biflora	19.574722
1312	Asparagaceae	Milla biflora	17.856667
1313	Asparagaceae	Milla biflora	17.857
1314	Asparagaceae	Milla biflora	19.45
1315	Asparagaceae	Milla biflora	19.523
1316	Asparagaceae	Milla biflora	19.523333
1317	Asparagaceae	Milla biflora	19.671667
1318	Asparagaceae	Milla biflora	19.672
1319	Asparagaceae	Milla biflora	20.74
1320	Asparagaceae	Milla biflora	23.184
1321	Asparagaceae	Milla biflora	22.57
1322	Asparagaceae	Milla biflora	22.584
1323	Asparagaceae	Milla biflora	23.973
1324	Asparagaceae	Milla biflora	23.973333
1325	Asparagaceae	Milla biflora	19.818
1326	Asparagaceae	Milla biflora	19.818333
1327	Asparagaceae	Milla biflora	21.086
1328	Asparagaceae	Milla biflora	21.14
1329	Asparagaceae	Milla biflora	19.524445
1330	Asparagaceae	Milla biflora	19.532223
1331	Asparagaceae	Milla biflora	19.855
1332	Asparagaceae	Milla biflora	19.895
1333	Asparagaceae	Milla biflora	19.895
1334	Asparagaceae	Milla biflora	19.895
1335	Asparagaceae	Milla biflora	19.831667
1336	Asparagaceae	Milla biflora	19.832
1337	Asparagaceae	Milla biflora	19.88861
1338	Asparagaceae	Milla biflora	22.25
1339	Asparagaceae	Milla biflora	22.25
1340	Asparagaceae	Milla biflora	19.561388
1341	Asparagaceae	Milla biflora	19.328056
1342	Asparagaceae	Milla biflora	21.405
1343	Asparagaceae	Milla biflora	21.405
1344	Asparagaceae	Milla biflora	18.716667
1345	Asparagaceae	Milla biflora	18.717
1346	Asparagaceae	Milla biflora	22.707
1347	Asparagaceae	Milla biflora	22.713
1348	Asparagaceae	Milla biflora	22.728
1349	Asparagaceae	Milla biflora	19.991667
1350	Asparagaceae	Milla biflora	19.992
1351	Asparagaceae	Milla biflora	20.0
1352	Asparagaceae	Milla biflora	18.88
1353	Asparagaceae	Milla biflora	18.88
1354	Asparagaceae	Milla biflora	20.283

1355	Asparagaceae	Milla biflora	20.317
1356	Asparagaceae	Milla biflora	20.338
1357	Asparagaceae	Milla biflora	20.366667
1358	Asparagaceae	Milla biflora	20.367
1359	Asparagaceae	Milla biflora	20.367
1360	Asparagaceae	Milla biflora	21.335
1361	Asparagaceae	Milla biflora	21.343
1362	Asparagaceae	Milla biflora	21.347
1363	Asparagaceae	Milla biflora	21.347
1364	Asparagaceae	Milla biflora	21.349
1365	Asparagaceae	Milla biflora	21.35
1366	Asparagaceae	Milla biflora	21.354
1367	Asparagaceae	Milla biflora	21.358
1368	Asparagaceae	Milla biflora	17.27
1369	Asparagaceae	Milla biflora	21.268
1370	Asparagaceae	Milla biflora	21.361666
1371	Asparagaceae	Milla biflora	21.362
1372	Asparagaceae	Milla biflora	21.118334
1373	Asparagaceae	Milla biflora	29.237
1374	Asparagaceae	Milla biflora	19.255556
1375	Asparagaceae	Milla biflora	18.817
1376	Asparagaceae	Milla biflora	20.501
1377	Asparagaceae	Milla biflora	19.913334
1378	Asparagaceae	Milla biflora	22.96
1379	Asparagaceae	Milla biflora	22.96
1380	Asparagaceae	Milla biflora	22.983
1381	Asparagaceae	Milla biflora	16.3
1382	Asparagaceae	Milla biflora	16.3
1383	Asparagaceae	Milla biflora	20.129723
1384	Asparagaceae	Milla biflora	20.131
1385	Asparagaceae	Milla biflora	18.663334
1386	Asparagaceae	Milla biflora	19.6
1387	Asparagaceae	Milla biflora	19.63
1388	Asparagaceae	Milla biflora	19.73
1389	Asparagaceae	Milla biflora	28.442
1390	Asparagaceae	Milla biflora	21.163
1391	Asparagaceae	Milla biflora	21.172
1392	Asparagaceae	Milla biflora	19.476389
1393	Asparagaceae	Milla biflora	22.18
1394	Asparagaceae	Milla biflora	19.778889
1395	Asparagaceae	Milla biflora	17.59
1396	Asparagaceae	Milla biflora	23.885
1397	Asparagaceae	Milla biflora	19.35
1398	Asparagaceae	Milla biflora	16.79
1399	Asparagaceae	Milla biflora	19.707222

1400	Asparagaceae	Milla biflora	20.125
1401	Asparagaceae	Milla biflora	20.134
1402	Asparagaceae	Milla biflora	18.316668
1403	Asparagaceae	Milla biflora	18.317
1404	Asparagaceae	Milla biflora	19.54
1405	Asparagaceae	Milla biflora	19.565
1406	Asparagaceae	Milla biflora	21.053
1407	Asparagaceae	Milla biflora	21.053333
1408	Asparagaceae	Milla biflora	18.75
1409	Asparagaceae	Milla biflora	21.886
1410	Asparagaceae	Milla biflora	23.668333
1411	Asparagaceae	Milla biflora	23.883
1412	Asparagaceae	Milla biflora	20.591667
1413	Asparagaceae	Milla biflora	20.592
1414	Asparagaceae	Milla biflora	29.33278
1415	Asparagaceae	Milla biflora	23.609
1416	Asparagaceae	Milla biflora	20.283
1417	Asparagaceae	Milla biflora	21.552
1418	Asparagaceae	Milla biflora	21.326944
1419	Asparagaceae	Milla biflora	21.327
1420	Asparagaceae	Milla biflora	21.548
1421	Asparagaceae	Milla biflora	21.548334
1422	Asparagaceae	Milla biflora	21.708
1423	Asparagaceae	Milla biflora	21.708334
1424	Asparagaceae	Milla biflora	19.78
1425	Asparagaceae	Milla biflora	24.242
1426	Asparagaceae	Milla biflora	22.134722
1427	Asparagaceae	Milla biflora	20.998
1428	Asparagaceae	Milla biflora	20.998333
1429	Asparagaceae	Milla biflora	21.072
1430	Asparagaceae	Milla biflora	17.677
1431	Asparagaceae	Milla biflora	17.815
1432	Asparagaceae	Milla biflora	20.3
1433	Asparagaceae	Milla biflora	21.19
1434	Asparagaceae	Milla biflora	21.2225
1435	Asparagaceae	Milla biflora	21.501667
1436	Asparagaceae	Milla biflora	21.502
1437	Asparagaceae	Milla biflora	22.121
1438	Asparagaceae	Milla biflora	22.1575
1439	Asparagaceae	Milla biflora	22.158
1440	Asparagaceae	Milla biflora	21.933
1441	Asparagaceae	Milla biflora	16.735556
1442	Asparagaceae	Milla biflora	19.083
1443	Asparagaceae	Milla biflora	19.132
1444	Asparagaceae	Milla biflora	31.307

1445	Asparagaceae	Milla biflora	31.26564
1446	Asparagaceae	Milla biflora	31.32056
1447	Asparagaceae	Milla biflora	16.726667
1448	Asparagaceae	Milla biflora	16.727
1449	Asparagaceae	Milla biflora	17.867
1450	Asparagaceae	Milla biflora	24.983
1451	Asparagaceae	Milla biflora	24.99
1452	Asparagaceae	Milla biflora	24.99
1453	Asparagaceae	Milla biflora	21.081667
1454	Asparagaceae	Milla biflora	21.082
1455	Asparagaceae	Milla biflora	23.756
1456	Asparagaceae	Milla biflora	23.0
1457	Asparagaceae	Milla biflora	23.331
1458	Asparagaceae	Milla biflora	23.409
1459	Asparagaceae	Milla biflora	23.417
1460	Asparagaceae	Milla biflora	23.45
1461	Asparagaceae	Milla biflora	23.45
1462	Asparagaceae	Milla biflora	23.62
1463	Asparagaceae	Milla biflora	23.620277
1464	Asparagaceae	Milla biflora	23.0
1465	Asparagaceae	Milla biflora	19.245
1466	Asparagaceae	Milla biflora	19.356
1467	Asparagaceae	Milla biflora	19.933
1468	Asparagaceae	Milla biflora	19.945
1469	Asparagaceae	Milla biflora	19.471666
1470	Asparagaceae	Milla biflora	19.472
1471	Asparagaceae	Milla biflora	18.84
1472	Asparagaceae	Milla biflora	19.03
1473	Asparagaceae	Milla biflora	18.985
1474	Asparagaceae	Milla biflora	18.683
1475	Asparagaceae	Milla biflora	19.783
1476	Asparagaceae	Milla biflora	19.838055
1477	Asparagaceae	Milla biflora	19.8425
1478	Asparagaceae	Milla biflora	20.763056
1479	Asparagaceae	Milla biflora	21.155
1480	Asparagaceae	Milla biflora	19.695555
1481	Asparagaceae	Milla biflora	19.731945
1482	Asparagaceae	Milla biflora	19.7575
1483	Asparagaceae	Milla biflora	19.7575
1484	Asparagaceae	Milla biflora	18.983
1485	Asparagaceae	Milla biflora	19.446667
1486	Asparagaceae	Milla biflora	19.449444
1487	Asparagaceae	Milla biflora	19.496666
1488	Asparagaceae	Milla biflora	19.567778
1489	Asparagaceae	Milla biflora	19.858334

1490	Asparagaceae	Milla biflora	19.3
1491	Asparagaceae	Milla biflora	19.208334
1492	Asparagaceae	Milla biflora	26.578
1493	Asparagaceae	Milla biflora	19.58
1494	Asparagaceae	Milla biflora	19.29
1495	Asparagaceae	Milla biflora	19.29
1496	Asparagaceae	Milla biflora	19.311945
1497	Asparagaceae	Milla biflora	19.3425
1498	Asparagaceae	Milla biflora	18.375
1499	Asparagaceae	Milla biflora	18.414
1500	Asparagaceae	Milla biflora	19.995
1501	Asparagaceae	Milla biflora	19.95861
1502	Asparagaceae	Milla biflora	20.908611
1503	Asparagaceae	Milla biflora	20.909
1504	Asparagaceae	Milla biflora	19.411667
1505	Asparagaceae	Milla biflora	19.412
1506	Asparagaceae	Milla biflora	19.645
1507	Asparagaceae	Milla biflora	19.600277
1508	Asparagaceae	Milla biflora	27.221
1509	Asparagaceae	Milla biflora	19.427
1510	Asparagaceae	Milla biflora	23.733
1511	Asparagaceae	Milla biflora	23.733334
1512	Asparagaceae	Milla biflora	21.2
1513	Asparagaceae	Milla biflora	21.293
1514	Asparagaceae	Milla biflora	21.293333
1515	Asparagaceae	Milla biflora	17.198
1516	Asparagaceae	Milla biflora	22.273
1517	Asparagaceae	Milla biflora	19.256666
1518	Asparagaceae	Milla biflora	28.333
1519	Asparagaceae	Milla biflora	28.333
1520	Asparagaceae	Milla biflora	28.374
1521	Asparagaceae	Milla biflora	28.392
1522	Asparagaceae	Milla biflora	20.8
1523	Asparagaceae	Milla biflora	22.029
1524	Asparagaceae	Milla biflora	22.064
1525	Asparagaceae	Milla biflora	19.708
1526	Asparagaceae	Milla biflora	19.708334
1527	Asparagaceae	Milla biflora	19.88
1528	Asparagaceae	Milla biflora	19.880278
1529	Asparagaceae	Milla biflora	19.924444
1530	Asparagaceae	Milla biflora	16.74
1531	Asparagaceae	Milla biflora	19.858
1532	Asparagaceae	Milla biflora	19.858334
1533	Asparagaceae	Milla biflora	19.47
1534	Asparagaceae	Milla biflora	17.89

1535	Asparagaceae	Milla biflora	18.18
1536	Asparagaceae	Milla biflora	18.71
1537	Asparagaceae	Milla biflora	19.04
1538	Asparagaceae	Milla biflora	19.69
1539	Asparagaceae	Milla biflora	19.73
1540	Asparagaceae	Milla biflora	19.92
1541	Asparagaceae	Milla biflora	20.99
1542	Asparagaceae	Milla biflora	22.05
1543	Asparagaceae	Milla biflora	23.91
1544	Asparagaceae	Milla biflora	23.91
1545	Asparagaceae	Milla biflora	23.92
1546	Asparagaceae	Milla biflora	24.05
1547	Asparagaceae	Milla biflora	27.11667
1548	Asparagaceae	Milla biflora	27.117
1549	Asparagaceae	Milla biflora	27.117
1550	Asparagaceae	Milla biflora	28.23889
1551	Asparagaceae	Milla biflora	28.239
1552	Asparagaceae	Milla biflora	28.239
1553	Asparagaceae	Milla biflora	28.36667
1554	Asparagaceae	Milla biflora	28.371666
1555	Asparagaceae	Milla biflora	28.86
1556	Asparagaceae	Milla biflora	29.379
1557	Asparagaceae	Milla biflora	29.379
1558	Asparagaceae	Milla biflora	29.98
1559	Asparagaceae	Milla bryanii	29.5113889
1560	Asparagaceae	Milla bryanii	27.1347222
1561	Asparagaceae	Milla magnifica	18.3952778
1562	Asparagaceae	Milla magnifica	18.6
1563	Asparagaceae	Milla oaxacana	17.55
1564	Asparagaceae	Milla oaxacana	17.11
1565	Asparagaceae	Milla oaxacana	17.6666667
1566	Asparagaceae	Milla rosea	25.0666667
1567	Asparagaceae	Brodiaea jolonensis	32
1568	Asparagaceae	Dandya hannibalii	18.69167
1569	Asparagaceae	Dandya purpusii	26.2
1570	Asparagaceae	Milla filifolia	18.714888
1571	Asparagaceae	Milla potosina	16.665012
1572	Asparagaceae	Milla delicata	17.073813
1573	Asparagaceae	Milla mexicana	18.152615
1574	Orchidaceae	Govenia alba	15.86667
1575	Orchidaceae	Govenia alba	16.96
1576	Orchidaceae	Govenia alba	19.573
1577	Orchidaceae	Govenia alba	21.218
1578	Orchidaceae	Govenia alba	21.95
1579	Orchidaceae	Govenia alba	23.08333

1580	Orchidaceae	Govenia bella	15.4272223
1581	Orchidaceae	Govenia bella	17.1
1582	Orchidaceae	Govenia dressleriana	17.1011111
1583	Orchidaceae	Govenia dressleriana	16.72
1584	Orchidaceae	Govenia dressleriana	17.726
1585	Orchidaceae	Govenia dressleriana	18.76639
1586	Orchidaceae	Govenia elliptica	25.6667
1587	Orchidaceae	Govenia lagenophora	25.91666
1588	Orchidaceae	Govenia lagenophora	19.0333333
1589	Orchidaceae	Govenia lagenophora	19.39972
1590	Orchidaceae	Govenia lagenophora	19.367
1591	Orchidaceae	Govenia liliacea	16.72
1592	Orchidaceae	Govenia liliacea	18.9
1593	Orchidaceae	Govenia liliacea	18.52
1594	Orchidaceae	Govenia liliacea	23.966667
1595	Orchidaceae	Govenia liliacea	22.05
1596	Orchidaceae	Govenia liliacea	23.53
1597	Orchidaceae	Govenia liliacea	16.67889
1598	Orchidaceae	Govenia liliacea	18.317
1599	Orchidaceae	Govenia liliacea	20.202
1600	Orchidaceae	Govenia liliacea	20.152
1601	Orchidaceae	Govenia liliacea	20.915
1602	Orchidaceae	Govenia liliacea	20.756
1603	Orchidaceae	Govenia liliacea	23.133
1604	Orchidaceae	Govenia liliacea	28.17222
1605	Orchidaceae	Govenia mutica	16.9972222
1606	Orchidaceae	Govenia mutica	21.95
1607	Orchidaceae	Govenia mutica	15.86667
1608	Orchidaceae	Govenia mutica	19.573
1609	Orchidaceae	Govenia mutica	21.218
1610	Orchidaceae	Govenia mutica	23.08333
1611	Orchidaceae	Govenia praecox	21.222
1612	Orchidaceae	Govenia praecox	18.834801
1613	Orchidaceae	Govenia purpusii	17.45
1614	Orchidaceae	Govenia purpusii	17.767
1615	Orchidaceae	Govenia purpusii	19.647
1616	Orchidaceae	Govenia purpusii	19.965
1617	Orchidaceae	Govenia purpusii	20.78
1618	Orchidaceae	Govenia purpusii	20.07
1619	Orchidaceae	Govenia purpusii	20.172
1620	Orchidaceae	Govenia rubellilabia	15.272657
1621	Orchidaceae	Govenia superba	16.77
1622	Orchidaceae	Govenia superba	18.16666
1623	Orchidaceae	Govenia superba	19.5833333
1624	Orchidaceae	Govenia superba	18.866667

1625	Orchidaceae	Govenia superba	19.45
1626	Orchidaceae	Govenia superba	18.99
1627	Orchidaceae	Govenia superba	16.7966666
1628	Orchidaceae	Govenia superba	18.52
1629	Orchidaceae	Govenia superba	18.26667
1630	Orchidaceae	Govenia superba	19.03472
1631	Orchidaceae	Govenia superba	20.915
1632	Orchidaceae	Govenia superba	23.417
1633	Orchidaceae	Govenia tequilana	20.78
1634	Orchidaceae	Bletia adenocarpa	16.28
1635	Orchidaceae	Bletia adenocarpa	17.62556
1636	Orchidaceae	Bletia adenocarpa	17.84111
1637	Orchidaceae	Bletia adenocarpa	17.24389
1638	Orchidaceae	Bletia adenocarpa	18.926
1639	Orchidaceae	Bletia adenocarpa	18.62
1640	Orchidaceae	Bletia adenocarpa	19.357
1641	Orchidaceae	Bletia adenocarpa	19.155
1642	Orchidaceae	Bletia adenocarpa	19.04
1643	Orchidaceae	Bletia adenocarpa	20.78
1644	Orchidaceae	Bletia adenocarpa	20.67
1645	Orchidaceae	Bletia adenocarpa	21.043
1646	Orchidaceae	Bletia adenocarpa	23.938
1647	Orchidaceae	Bletia adenocarpa	23.496
1648	Orchidaceae	Bletia adenocarpa	24.167
1649	Orchidaceae	Bletia adenocarpa	24.632
1650	Orchidaceae	Bletia adenocarpa	20.87279
1651	Orchidaceae	Bletia adenocarpa	18.892939
1652	Orchidaceae	Bletia adenocarpa	16.27847
1653	Orchidaceae	Bletia adenocarpa	17.317965
1654	Orchidaceae	Bletia adenocarpa	20.701655
1655	Orchidaceae	Bletia adenocarpa	20.860144
1656	Orchidaceae	Bletia adenocarpa	18.979191
1657	Orchidaceae	Bletia adenocarpa	17.316728
1658	Orchidaceae	Bletia adenocarpa	20.777349
1659	Orchidaceae	Bletia adenocarpa	19.308154
1660	Orchidaceae	Bletia campanulata	-16.35
1661	Orchidaceae	Bletia campanulata	-16.7261
1662	Orchidaceae	Bletia campanulata	-15.22
1663	Orchidaceae	Bletia campanulata	8.79
1664	Orchidaceae	Bletia campanulata	9.76611
1665	Orchidaceae	Bletia campanulata	9.8283
1666	Orchidaceae	Bletia campanulata	12.97
1667	Orchidaceae	Bletia campanulata	12.93
1668	Orchidaceae	Bletia campanulata	13.73
1669	Orchidaceae	Bletia campanulata	13.05

1670	Orchidaceae	Bletia campanulata	13.75
1671	Orchidaceae	Bletia campanulata	14.14
1672	Orchidaceae	Bletia campanulata	14.3
1673	Orchidaceae	Bletia campanulata	15.506
1674	Orchidaceae	Bletia campanulata	16.24944
1675	Orchidaceae	Bletia campanulata	16.30167
1676	Orchidaceae	Bletia campanulata	16.71667
1677	Orchidaceae	Bletia campanulata	16.683
1678	Orchidaceae	Bletia campanulata	16.709
1679	Orchidaceae	Bletia campanulata	16.41278
1680	Orchidaceae	Bletia campanulata	16.734
1681	Orchidaceae	Bletia campanulata	18.897
1682	Orchidaceae	Bletia campanulata	18.45
1683	Orchidaceae	Bletia campanulata	18.967
1684	Orchidaceae	Bletia campanulata	19.662
1685	Orchidaceae	Bletia campanulata	19.155
1686	Orchidaceae	Bletia campanulata	19.48
1687	Orchidaceae	Bletia campanulata	19.383
1688	Orchidaceae	Bletia campanulata	19.752
1689	Orchidaceae	Bletia campanulata	19.405
1690	Orchidaceae	Bletia campanulata	19.63
1691	Orchidaceae	Bletia campanulata	19.902
1692	Orchidaceae	Bletia campanulata	19.329
1693	Orchidaceae	Bletia campanulata	19.314
1694	Orchidaceae	Bletia campanulata	19.1
1695	Orchidaceae	Bletia campanulata	19.317
1696	Orchidaceae	Bletia campanulata	20.3333
1697	Orchidaceae	Bletia campanulata	21.241
1698	Orchidaceae	Bletia campanulata	21.357
1699	Orchidaceae	Bletia campanulata	21.033
1700	Orchidaceae	Bletia campanulata	23.779
1701	Orchidaceae	Bletia campanulata	19.051636
1702	Orchidaceae	Bletia campanulata	19.406748
1703	Orchidaceae	Bletia campanulata	17.528711
1704	Orchidaceae	Bletia campanulata	16.206489
1705	Orchidaceae	Bletia campanulata	18.95938
1706	Orchidaceae	Bletia campanulata	18.957515
1707	Orchidaceae	Bletia campanulata	13.746429
1708	Orchidaceae	Bletia campanulata	23.844629
1709	Orchidaceae	Bletia campanulata	15.517461
1710	Orchidaceae	Bletia campanulata	21.374591
1711	Orchidaceae	Bletia campanulata	19.246107
1712	Orchidaceae	Bletia campanulata	12.565684
1713	Orchidaceae	Bletia campanulata	21.521063
1714	Orchidaceae	Bletia campanulata	10.679378

1715	Orchidaceae	Bletia campanulata	13.709004
1716	Orchidaceae	Bletia campanulata	16.773519
1717	Orchidaceae	Bletia campanulata	19.576666
1718	Orchidaceae	Bletia campanulata	19.532162
1719	Orchidaceae	Bletia campanulata	20.66155
1720	Orchidaceae	Bletia campanulata	15.427842
1721	Orchidaceae	Bletia campanulata	17.774539
1722	Orchidaceae	Bletia campanulata	14.023551
1723	Orchidaceae	Bletia campanulata	22.1833
1724	Orchidaceae	Bletia campanulata	18.898451
1725	Orchidaceae	Bletia campanulata	19.488471
1726	Orchidaceae	Bletia campanulata	22.000531
1727	Orchidaceae	Bletia campanulata	19.378071
1728	Orchidaceae	Bletia campanulata	19.649543
1729	Orchidaceae	Bletia campanulata	16.529149
1730	Orchidaceae	Bletia campanulata	18.952224
1731	Orchidaceae	Bletia campanulata	16.304645
1732	Orchidaceae	Bletia campanulata	-15.548454
1733	Orchidaceae	Bletia campanulata	17.118537
1734	Orchidaceae	Bletia campanulata	18.914576
1735	Orchidaceae	Bletia campanulata	15.50058
1736	Orchidaceae	Bletia campanulata	18.858232
1737	Orchidaceae	Bletia campanulata	19.314184
1738	Orchidaceae	Bletia campanulata	23.031022
1739	Orchidaceae	Bletia campanulata	2.438696
1740	Orchidaceae	Bletia campanulata	18.795708
1741	Orchidaceae	Bletia campanulata	20.726385
1742	Orchidaceae	Bletia campanulata	13.865239
1743	Orchidaceae	Bletia campanulata	13.798524
1744	Orchidaceae	Bletia campanulata	19.359088
1745	Orchidaceae	Bletia campanulata	19.063418
1746	Orchidaceae	Bletia campanulata	-1.433316
1747	Orchidaceae	Bletia campanulata	13.418257
1748	Orchidaceae	Bletia campanulata	20.448555
1749	Orchidaceae	Bletia campanulata	19.479589
1750	Orchidaceae	Bletia coccinea	20.215725
1751	Orchidaceae	Bletia coccinea	20.652575
1752	Orchidaceae	Bletia coccinea	18.910126
1753	Orchidaceae	Bletia coccinea	18.221623
1754	Orchidaceae	Bletia coccinea	18.892228
1755	Orchidaceae	Bletia coccinea	19.306497
1756	Orchidaceae	Bletia coccinea	18.775423
1757	Orchidaceae	Bletia coccinea	18.975139
1758	Orchidaceae	Bletia coccinea	16.68444
1759	Orchidaceae	Bletia coccinea	17.633

1760	Orchidaceae	Bletia coccinea	17.83694
1761	Orchidaceae	Bletia coccinea	17.583
1762	Orchidaceae	Bletia coccinea	17.646
1763	Orchidaceae	Bletia coccinea	18.66944
1764	Orchidaceae	Bletia coccinea	18.87
1765	Orchidaceae	Bletia coccinea	18.95
1766	Orchidaceae	Bletia coccinea	18.967
1767	Orchidaceae	Bletia coccinea	19.155
1768	Orchidaceae	Bletia coccinea	19.04
1769	Orchidaceae	Bletia coccinea	19.1
1770	Orchidaceae	Bletia coccinea	20.8
1771	Orchidaceae	Bletia coccinea	20.67
1772	Orchidaceae	Bletia coccinea	20.935
1773	Orchidaceae	Bletia coccinea	30.696
1774	Orchidaceae	Bletia greenwoodiana	22.798426
1775	Orchidaceae	Bletia ensifolia	20.216293
1776	Orchidaceae	Bletia ensifolia	20.674805
1777	Orchidaceae	Bletia ensifolia	26.343029
1778	Orchidaceae	Bletia ensifolia	23.52897
1779	Orchidaceae	Bletia ensifolia	23.949593
1780	Orchidaceae	Bletia ensifolia	20.501599
1781	Orchidaceae	Bletia ensifolia	21.919734
1782	Orchidaceae	Bletia ensifolia	20.724693
1783	Orchidaceae	Bletia ensifolia	20.733844
1784	Orchidaceae	Bletia ensifolia	18.8
1785	Orchidaceae	Bletia ensifolia	20.533
1786	Orchidaceae	Bletia ensifolia	21.50472
1787	Orchidaceae	Bletia ensifolia	21.832
1788	Orchidaceae	Bletia ensifolia	22.96
1789	Orchidaceae	Bletia ensifolia	23.236
1790	Orchidaceae	Bletia ensifolia	23.33056
1791	Orchidaceae	Bletia ensifolia	23
1792	Orchidaceae	Bletia gracilis	19.691684
1793	Orchidaceae	Bletia gracilis	21.237864
1794	Orchidaceae	Bletia gracilis	21.614883
1795	Orchidaceae	Bletia gracilis	18.620004
1796	Orchidaceae	Bletia gracilis	18.934985
1797	Orchidaceae	Bletia gracilis	19.013384
1798	Orchidaceae	Bletia gracilis	18.852858
1799	Orchidaceae	Bletia gracilis	18.433387
1800	Orchidaceae	Bletia gracilis	19.248784
1801	Orchidaceae	Bletia gracilis	19.407068
1802	Orchidaceae	Bletia gracilis	18.828922
1803	Orchidaceae	Bletia gracilis	17.292582
1804	Orchidaceae	Bletia gracilis	18.969682

1805	Orchidaceae	Bletia gracilis	19.637431
1806	Orchidaceae	Bletia gracilis	21.374246
1807	Orchidaceae	Bletia gracilis	18.620137
1808	Orchidaceae	Bletia gracilis	16.2
1809	Orchidaceae	Bletia gracilis	17.23
1810	Orchidaceae	Bletia gracilis	18.189
1811	Orchidaceae	Bletia gracilis	18.951
1812	Orchidaceae	Bletia gracilis	18.468
1813	Orchidaceae	Bletia gracilis	18.917
1814	Orchidaceae	Bletia gracilis	18.51111
1815	Orchidaceae	Bletia gracilis	18.95
1816	Orchidaceae	Bletia gracilis	19.418
1817	Orchidaceae	Bletia gracilis	19.6
1818	Orchidaceae	Bletia gracilis	19
1819	Orchidaceae	Bletia gracilis	21.241
1820	Orchidaceae	Bletia gracilis	27.06
1821	Orchidaceae	Bletia gracilis	27.32667
1822	Orchidaceae	Bletia gracilis	27.117
1823	Orchidaceae	Bletia gracilis	27.6
1824	Orchidaceae	Bletia gracilis	27.35
1825	Orchidaceae	Bletia gracilis	28.38333
1826	Orchidaceae	Bletia gracilis	28.5
1827	Orchidaceae	Bletia gracilis	28.41306
1828	Orchidaceae	Bletia lilacina	17.836943
1829	Orchidaceae	Bletia lilacina	17.11892
1830	Orchidaceae	Bletia lilacina	17.126324
1831	Orchidaceae	Bletia lilacina	15.95917
1832	Orchidaceae	Bletia lilacina	17.1
1833	Orchidaceae	Bletia lilacina	17.111
1834	Orchidaceae	Bletia lilacina	18.85
1835	Orchidaceae	Bletia lilacina	18.863
1836	Orchidaceae	Bletia lilacina	18.55
1837	Orchidaceae	Bletia lilacina	18.937
1838	Orchidaceae	Bletia lilacina	18.87
1839	Orchidaceae	Bletia lilacina	20.347
1840	Orchidaceae	Bletia macristhmochila	20.240081
1841	Orchidaceae	Bletia macristhmochila	19.747936
1842	Orchidaceae	Bletia macristhmochila	20.809442
1843	Orchidaceae	Bletia macristhmochila	25.621746
1844	Orchidaceae	Bletia macristhmochila	17.575877
1845	Orchidaceae	Bletia macristhmochila	19.212957
1846	Orchidaceae	Bletia macristhmochila	19.869467
1847	Orchidaceae	Bletia macristhmochila	18.888185
1848	Orchidaceae	Bletia macristhmochila	21.143802
1849	Orchidaceae	Bletia macristhmochila	20.659958

1850	Orchidaceae	Bletia macristhmochila	17.333
1851	Orchidaceae	Bletia macristhmochila	18.87
1852	Orchidaceae	Bletia macristhmochila	18.967
1853	Orchidaceae	Bletia macristhmochila	18.467
1854	Orchidaceae	Bletia macristhmochila	18.87
1855	Orchidaceae	Bletia macristhmochila	18.883
1856	Orchidaceae	Bletia macristhmochila	19.155
1857	Orchidaceae	Bletia macristhmochila	19.818
1858	Orchidaceae	Bletia macristhmochila	19.502
1859	Orchidaceae	Bletia macristhmochila	19.457
1860	Orchidaceae	Bletia macristhmochila	20.633
1861	Orchidaceae	Bletia macristhmochila	21.817
1862	Orchidaceae	Bletia macristhmochila	21.333
1863	Orchidaceae	Bletia macristhmochila	22.783
1864	Orchidaceae	Bletia macristhmochila	23.585
1865	Orchidaceae	Bletia macristhmochila	23.779
1866	Orchidaceae	Bletia macristhmochila	24.167
1867	Orchidaceae	Bletia macristhmochila	24.23333
1868	Orchidaceae	Bletia macristhmochila	24
1869	Orchidaceae	Bletia neglecta	19.664
1870	Orchidaceae	Bletia neglecta	19.043
1871	Orchidaceae	Bletia neglecta	19
1872	Orchidaceae	Bletia neglecta	19.067
1873	Orchidaceae	Bletia neglecta	19.92944
1874	Orchidaceae	Bletia neglecta	20.493
1875	Orchidaceae	Bletia neglecta	20.483
1876	Orchidaceae	Bletia neglecta	20.915
1877	Orchidaceae	Bletia neglecta	20.633
1878	Orchidaceae	Bletia neglecta	21.396
1879	Orchidaceae	Bletia neglecta	21.067
1880	Orchidaceae	Bletia neglecta	19.167565
1881	Orchidaceae	Bletia neglecta	19.154287
1882	Orchidaceae	Bletia neglecta	19.024015
1883	Orchidaceae	Bletia neglecta	19.040961
1884	Orchidaceae	Bletia neglecta	19.842007
1885	Orchidaceae	Bletia neglecta	19.741796
1886	Orchidaceae	Bletia neglecta	19.44972
1887	Orchidaceae	Bletia neglecta	20.995376
1888	Orchidaceae	Bletia neglecta	20.168743
1889	Orchidaceae	Bletia neglecta	19.71005
1890	Orchidaceae	Bletia neglecta	19.283235
1891	Orchidaceae	Bletia neglecta	19.23122
1892	Orchidaceae	Bletia neglecta	20.3833
1893	Orchidaceae	Bletia neglecta	19.800042
1894	Orchidaceae	Bletia neglecta	19.021649

1895	Orchidaceae	Bletia neglecta	21.83714
1896	Orchidaceae	Bletia neglecta	19.142344
1897	Orchidaceae	Bletia neglecta	20.349963
1898	Orchidaceae	Bletia neglecta	18.964838
1899	Orchidaceae	Bletia neglecta	18.85213
1900	Orchidaceae	Bletia neglecta	19.0164
1901	Orchidaceae	Bletia parkinsonii	17.212301
1902	Orchidaceae	Bletia parkinsonii	18.408489
1903	Orchidaceae	Bletia parkinsonii	19.430049
1904	Orchidaceae	Bletia parkinsonii	18.969602
1905	Orchidaceae	Bletia parkinsonii	18.609786
1906	Orchidaceae	Bletia parkinsonii	17.496234
1907	Orchidaceae	Bletia parkinsonii	18.456399
1908	Orchidaceae	Bletia parkinsonii	19.95
1909	Orchidaceae	Bletia parkinsonii	16.063
1910	Orchidaceae	Bletia parkinsonii	17.487
1911	Orchidaceae	Bletia parkinsonii	17.75
1912	Orchidaceae	Bletia parkinsonii	17.483
1913	Orchidaceae	Bletia parkinsonii	17.583
1914	Orchidaceae	Bletia parkinsonii	17.95
1915	Orchidaceae	Bletia parkinsonii	17.268
1916	Orchidaceae	Bletia parkinsonii	17.59306
1917	Orchidaceae	Bletia parkinsonii	18.802
1918	Orchidaceae	Bletia parkinsonii	18.85
1919	Orchidaceae	Bletia parkinsonii	19.053
1920	Orchidaceae	Bletia parkinsonii	19.147
1921	Orchidaceae	Bletia parkinsonii	19.468
1922	Orchidaceae	Bletia parkinsonii	19.336
1923	Orchidaceae	Bletia parkinsonii	19.433
1924	Orchidaceae	Bletia parkinsonii	20.983
1925	Orchidaceae	Bletia parkinsonii	23.965
1926	Orchidaceae	Bletia punctata	19.452465
1927	Orchidaceae	Bletia punctata	17.120783
1928	Orchidaceae	Bletia punctata	19.03833
1929	Orchidaceae	Bletia punctata	20.809495
1930	Orchidaceae	Bletia punctata	20.648
1931	Orchidaceae	Bletia punctata	19.669143
1932	Orchidaceae	Bletia punctata	20.588884
1933	Orchidaceae	Bletia punctata	19.412366
1934	Orchidaceae	Bletia punctata	19.647194
1935	Orchidaceae	Bletia punctata	18.970542
1936	Orchidaceae	Bletia punctata	17.11892
1937	Orchidaceae	Bletia punctata	16.416696
1938	Orchidaceae	Bletia punctata	20.87453
1939	Orchidaceae	Bletia punctata	16.01667

1940	Orchidaceae	Bletia punctata	17.65083
1941	Orchidaceae	Bletia punctata	17.41667
1942	Orchidaceae	Bletia punctata	17.86861
1943	Orchidaceae	Bletia punctata	18.926
1944	Orchidaceae	Bletia punctata	18.467
1945	Orchidaceae	Bletia punctata	19.779
1946	Orchidaceae	Bletia punctata	19.428
1947	Orchidaceae	Bletia punctata	19.818
1948	Orchidaceae	Bletia punctata	19.04
1949	Orchidaceae	Bletia punctata	19.019
1950	Orchidaceae	Bletia punctata	21.33306
1951	Orchidaceae	Bletia purpurata	19.113091
1952	Orchidaceae	Bletia purpurata	15.567369
1953	Orchidaceae	Bletia purpurata	19.399948
1954	Orchidaceae	Bletia purpurata	19.489835
1955	Orchidaceae	Bletia purpurata	18.987387
1956	Orchidaceae	Bletia purpurata	25.436746
1957	Orchidaceae	Bletia purpurata	19.101482
1958	Orchidaceae	Bletia purpurata	8.24
1959	Orchidaceae	Bletia purpurata	10.02
1960	Orchidaceae	Bletia purpurata	12.93
1961	Orchidaceae	Bletia purpurata	13.73
1962	Orchidaceae	Bletia purpurata	15.86667
1963	Orchidaceae	Bletia purpurata	17.18
1964	Orchidaceae	Bletia purpurata	17.267
1965	Orchidaceae	Bletia purpurata	18.79
1966	Orchidaceae	Bletia purpurata	18.9
1967	Orchidaceae	Bletia purpurata	18.967
1968	Orchidaceae	Bletia purpurata	18.483
1969	Orchidaceae	Bletia purpurata	18.459
1970	Orchidaceae	Bletia purpurata	18.967
1971	Orchidaceae	Bletia purpurata	18.783
1972	Orchidaceae	Bletia purpurata	18.08583
1973	Orchidaceae	Bletia purpurata	19.7
1974	Orchidaceae	Bletia purpurata	19.367
1975	Orchidaceae	Bletia purpurata	19.707
1976	Orchidaceae	Bletia purpurata	19.64056
1977	Orchidaceae	Bletia purpurata	19.530315
1978	Orchidaceae	Bletia purpurata	19.191804
1979	Orchidaceae	Bletia purpurata	19.043267
1980	Orchidaceae	Bletia purpurata	19.683
1981	Orchidaceae	Bletia purpurata	19.29
1982	Orchidaceae	Bletia purpurata	20.285
1983	Orchidaceae	Bletia purpurata	21.388
1984	Orchidaceae	Bletia purpurata	21.182

1985	Orchidaceae	Bletia purpurata	22.32
1986	Orchidaceae	Bletia purpurata	23.233
1987	Orchidaceae	Bletia purpurata	23.517
1988	Orchidaceae	Bletia purpurata	23.454
1989	Orchidaceae	Bletia purpurata	26.392
1990	Orchidaceae	Bletia purpurata	27.608
1991	Orchidaceae	Bletia purpurea	15.778722
1992	Orchidaceae	Bletia purpurea	15.480115
1993	Orchidaceae	Bletia purpurea	16.805407
1994	Orchidaceae	Bletia purpurea	17.411655
1995	Orchidaceae	Bletia purpurea	9.993812
1996	Orchidaceae	Bletia purpurea	9.835318
1997	Orchidaceae	Bletia purpurea	8.639282
1998	Orchidaceae	Bletia purpurea	15.479288
1999	Orchidaceae	Bletia purpurea	16.772912
2000	Orchidaceae	Bletia purpurea	9.25025
2001	Orchidaceae	Bletia purpurea	14.605799
2002	Orchidaceae	Bletia purpurea	6.34802
2003	Orchidaceae	Bletia purpurea	14.015935
2004	Orchidaceae	Bletia purpurea	13.62559
2005	Orchidaceae	Bletia purpurea	14.605095
2006	Orchidaceae	Bletia purpurea	15.761649
2007	Orchidaceae	Bletia purpurea	-2.58849
2008	Orchidaceae	Bletia purpurea	15.355609
2009	Orchidaceae	Bletia purpurea	15.504367
2010	Orchidaceae	Bletia purpurea	15.479694
2011	Orchidaceae	Bletia purpurea	14.608479
2012	Orchidaceae	Bletia purpurea	14.911309
2013	Orchidaceae	Bletia purpurea	18.442503
2014	Orchidaceae	Bletia purpurea	-0.583694
2015	Orchidaceae	Bletia purpurea	14.618876
2016	Orchidaceae	Bletia purpurea	17.186313
2017	Orchidaceae	Bletia purpurea	16.082335
2018	Orchidaceae	Bletia purpurea	18.854724
2019	Orchidaceae	Bletia purpurea	18.608047
2020	Orchidaceae	Bletia purpurea	18.918993
2021	Orchidaceae	Bletia purpurea	17.443971
2022	Orchidaceae	Bletia purpurea	21.186368
2023	Orchidaceae	Bletia purpurea	16.962713
2024	Orchidaceae	Bletia purpurea	20.244224
2025	Orchidaceae	Bletia purpurea	21.859572
2026	Orchidaceae	Bletia purpurea	22.359565
2027	Orchidaceae	Bletia purpurea	18.575718
2028	Orchidaceae	Bletia purpurea	21.683757
2029	Orchidaceae	Bletia purpurea	19.388598

2030	Orchidaceae	Bletia purpurea	16.323087
2031	Orchidaceae	Bletia purpurea	17.356314
2032	Orchidaceae	Bletia purpurea	18.183447
2033	Orchidaceae	Bletia purpurea	18.782527
2034	Orchidaceae	Bletia purpurea	18.970594
2035	Orchidaceae	Bletia purpurea	18.571441
2036	Orchidaceae	Bletia purpurea	20.859469
2037	Orchidaceae	Bletia purpurea	21.211906
2038	Orchidaceae	Bletia purpurea	20.554835
2039	Orchidaceae	Bletia purpurea	20.671733
2040	Orchidaceae	Bletia purpurea	18.649176
2041	Orchidaceae	Bletia purpurea	18.96845
2042	Orchidaceae	Bletia purpurea	18.607979
2043	Orchidaceae	Bletia purpurea	19.223121
2044	Orchidaceae	Bletia purpurea	18.6166667
2045	Orchidaceae	Bletia purpurea	16.653527
2046	Orchidaceae	Bletia purpurea	17.419427
2047	Orchidaceae	Bletia purpurea	15.990218
2048	Orchidaceae	Bletia purpurea	17.229684
2049	Orchidaceae	Bletia purpurea	28.739868
2050	Orchidaceae	Bletia purpurea	16.962713
2051	Orchidaceae	Bletia purpurea	8.790927
2052	Orchidaceae	Bletia purpurea	11.83333
2053	Orchidaceae	Bletia purpurea	21.249354
2054	Orchidaceae	Bletia purpurea	18.921573
2055	Orchidaceae	Bletia purpurea	20.998367
2056	Orchidaceae	Bletia purpurea	19.391552
2057	Orchidaceae	Bletia purpurea	17.958939
2058	Orchidaceae	Bletia purpurea	17.970722
2059	Orchidaceae	Bletia purpurea	22.335097
2060	Orchidaceae	Bletia purpurea	18.347288
2061	Orchidaceae	Bletia purpurea	24.683
2062	Orchidaceae	Bletia purpurea	15.47
2063	Orchidaceae	Bletia purpurea	19.890549
2064	Orchidaceae	Bletia purpurea	18.489659
2065	Orchidaceae	Bletia purpurea	19.3852778
2066	Orchidaceae	Bletia purpurea	11.971169
2067	Orchidaceae	Bletia purpurea	11.988038
2068	Orchidaceae	Bletia purpurea	8.788802
2069	Orchidaceae	Bletia purpurea	15.355609
2070	Orchidaceae	Bletia purpurea	11.83333
2071	Orchidaceae	Bletia purpurea	11.48
2072	Orchidaceae	Bletia purpurea	18.84
2073	Orchidaceae	Bletia purpurea	8.263732
2074	Orchidaceae	Bletia purpurea	7.920319

2075	Orchidaceae	Bletia purpurea	14.6
2076	Orchidaceae	Bletia purpurea	13.998551
2077	Orchidaceae	Bletia purpurea	14.0916667
2078	Orchidaceae	Bletia purpurea	8.95
2079	Orchidaceae	Bletia purpurea	8.75417
2080	Orchidaceae	Bletia purpurea	8.49722
2081	Orchidaceae	Bletia purpurea	8.68
2082	Orchidaceae	Bletia purpurea	8.43
2083	Orchidaceae	Bletia purpurea	8.95
2084	Orchidaceae	Bletia purpurea	9.91
2085	Orchidaceae	Bletia purpurea	9.65278
2086	Orchidaceae	Bletia purpurea	9.11791
2087	Orchidaceae	Bletia purpurea	9.17
2088	Orchidaceae	Bletia purpurea	10.92917
2089	Orchidaceae	Bletia purpurea	10.11528
2090	Orchidaceae	Bletia purpurea	10.28
2091	Orchidaceae	Bletia purpurea	10.09
2092	Orchidaceae	Bletia purpurea	11.98
2093	Orchidaceae	Bletia purpurea	11.54
2094	Orchidaceae	Bletia purpurea	12.97
2095	Orchidaceae	Bletia purpurea	13.13
2096	Orchidaceae	Bletia purpurea	13.77
2097	Orchidaceae	Bletia purpurea	14.6
2098	Orchidaceae	Bletia purpurea	14.97
2099	Orchidaceae	Bletia purpurea	15.857
2100	Orchidaceae	Bletia purpurea	15.701
2101	Orchidaceae	Bletia purpurea	15.446
2102	Orchidaceae	Bletia purpurea	15.684
2103	Orchidaceae	Bletia purpurea	15.927
2104	Orchidaceae	Bletia purpurea	15.52
2105	Orchidaceae	Bletia purpurea	16.967
2106	Orchidaceae	Bletia purpurea	16.68444
2107	Orchidaceae	Bletia purpurea	16.7508333
2108	Orchidaceae	Bletia purpurea	16.312
2109	Orchidaceae	Bletia purpurea	16.8086111
2110	Orchidaceae	Bletia purpurea	16.83
2111	Orchidaceae	Bletia purpurea	16.738
2112	Orchidaceae	Bletia purpurea	16.03333
2113	Orchidaceae	Bletia purpurea	16.7722222
2114	Orchidaceae	Bletia purpurea	16.87
2115	Orchidaceae	Bletia purpurea	16.94
2116	Orchidaceae	Bletia purpurea	17.177
2117	Orchidaceae	Bletia purpurea	17.1805556
2118	Orchidaceae	Bletia purpurea	17.8244444
2119	Orchidaceae	Bletia purpurea	17.267

2120	Orchidaceae	Bletia purpurea	17.1877778
2121	Orchidaceae	Bletia purpurea	17.3513889
2122	Orchidaceae	Bletia purpurea	17.229
2123	Orchidaceae	Bletia purpurea	17.21
2124	Orchidaceae	Bletia purpurea	17.983
2125	Orchidaceae	Bletia purpurea	17.767
2126	Orchidaceae	Bletia purpurea	17.384
2127	Orchidaceae	Bletia purpurea	17.99778
2128	Orchidaceae	Bletia purpurea	17.9305556
2129	Orchidaceae	Bletia purpurea	17.83
2130	Orchidaceae	Bletia purpurea	18.63
2131	Orchidaceae	Bletia purpurea	18.334
2132	Orchidaceae	Bletia purpurea	18.84
2133	Orchidaceae	Bletia purpurea	18.6166667
2134	Orchidaceae	Bletia purpurea	18.55
2135	Orchidaceae	Bletia purpurea	18.783
2136	Orchidaceae	Bletia purpurea	18.905
2137	Orchidaceae	Bletia purpurea	18.24056
2138	Orchidaceae	Bletia purpurea	18.45
2139	Orchidaceae	Bletia purpurea	18.59
2140	Orchidaceae	Bletia purpurea	18.585
2141	Orchidaceae	Bletia purpurea	18.272
2142	Orchidaceae	Bletia purpurea	18.10333
2143	Orchidaceae	Bletia purpurea	18.1836111
2144	Orchidaceae	Bletia purpurea	18
2145	Orchidaceae	Bletia purpurea	18.54
2146	Orchidaceae	Bletia purpurea	18.09667
2147	Orchidaceae	Bletia purpurea	18.352
2148	Orchidaceae	Bletia purpurea	18.172
2149	Orchidaceae	Bletia purpurea	18.6125
2150	Orchidaceae	Bletia purpurea	18.2466667
2151	Orchidaceae	Bletia purpurea	18.8105556
2152	Orchidaceae	Bletia purpurea	18.97
2153	Orchidaceae	Bletia purpurea	19.602
2154	Orchidaceae	Bletia purpurea	19.5083333
2155	Orchidaceae	Bletia purpurea	19.808
2156	Orchidaceae	Bletia purpurea	19.1183333
2157	Orchidaceae	Bletia purpurea	19.93
2158	Orchidaceae	Bletia purpurea	19.383
2159	Orchidaceae	Bletia purpurea	19.0472222
2160	Orchidaceae	Bletia purpurea	19.203
2161	Orchidaceae	Bletia purpurea	19.328
2162	Orchidaceae	Bletia purpurea	19.4805556
2163	Orchidaceae	Bletia purpurea	19.21
2164	Orchidaceae	Bletia purpurea	19.52

2165	Orchidaceae	Bletia purpurea	20.392
2166	Orchidaceae	Bletia purpurea	20.512
2167	Orchidaceae	Bletia purpurea	20.767
2168	Orchidaceae	Bletia purpurea	20.3002778
2169	Orchidaceae	Bletia purpurea	20.39
2170	Orchidaceae	Bletia purpurea	20.520303
2171	Orchidaceae	Bletia purpurea	21.212
2172	Orchidaceae	Bletia purpurea	21.8605556
2173	Orchidaceae	Bletia purpurea	21.1875
2174	Orchidaceae	Bletia purpurea	21.398
2175	Orchidaceae	Bletia purpurea	21.15
2176	Orchidaceae	Bletia purpurea	21.717
2177	Orchidaceae	Bletia purpurea	22.25
2178	Orchidaceae	Bletia purpurea	22.58
2179	Orchidaceae	Bletia purpurea	22.05
2180	Orchidaceae	Bletia purpurea	22.227
2181	Orchidaceae	Bletia purpurea	23.389
2182	Orchidaceae	Bletia purpurea	26.98333
2183	Orchidaceae	Bletia riparia	19.282
2184	Orchidaceae	Bletia riparia	19.21
2185	Orchidaceae	Bletia roezlii	18.587004
2186	Orchidaceae	Bletia roezlii	18.996262
2187	Orchidaceae	Bletia roezlii	17.120783
2188	Orchidaceae	Bletia roezlii	20.438385
2189	Orchidaceae	Bletia roezlii	19.248843
2190	Orchidaceae	Bletia roezlii	17.558369
2191	Orchidaceae	Bletia roezlii	18.593631
2192	Orchidaceae	Bletia roezlii	17.289807
2193	Orchidaceae	Bletia roezlii	19.877795
2194	Orchidaceae	Bletia roezlii	20.753742
2195	Orchidaceae	Bletia roezlii	18.793935
2196	Orchidaceae	Bletia roezlii	18.921152
2197	Orchidaceae	Bletia roezlii	19.861021
2198	Orchidaceae	Bletia roezlii	19.31
2199	Orchidaceae	Bletia roezlii	18.590713
2200	Orchidaceae	Bletia roezlii	21.355274
2201	Orchidaceae	Bletia roezlii	12.98
2202	Orchidaceae	Bletia roezlii	13.95
2203	Orchidaceae	Bletia roezlii	13.37
2204	Orchidaceae	Bletia roezlii	14.33
2205	Orchidaceae	Bletia roezlii	14.13
2206	Orchidaceae	Bletia roezlii	14.56
2207	Orchidaceae	Bletia roezlii	16.03361
2208	Orchidaceae	Bletia roezlii	16.567
2209	Orchidaceae	Bletia roezlii	17.266

2210	Orchidaceae	Bletia roezlii	18.963
2211	Orchidaceae	Bletia roezlii	18.88
2212	Orchidaceae	Bletia roezlii	18.918
2213	Orchidaceae	Bletia roezlii	18.467
2214	Orchidaceae	Bletia roezlii	18.62
2215	Orchidaceae	Bletia roezlii	18.857
2216	Orchidaceae	Bletia roezlii	18.98
2217	Orchidaceae	Bletia roezlii	18.828
2218	Orchidaceae	Bletia roezlii	19.529
2219	Orchidaceae	Bletia roezlii	19.567
2220	Orchidaceae	Bletia roezlii	19.107
2221	Orchidaceae	Bletia roezlii	20.195
2222	Orchidaceae	Bletia roezlii	20.648
2223	Orchidaceae	Bletia roezlii	20.388
2224	Orchidaceae	Bletia roezlii	21.35
2225	Orchidaceae	Bletia roezlii	21.11
2226	Orchidaceae	Bletia roezlii	22.251
2227	Orchidaceae	Bletia roezlii	23.938
2228	Orchidaceae	Bletia roezlii	24.167
2229	Orchidaceae	Bletia roezlii	25.824
2230	Orchidaceae	Bletia roezlii	27.3
2231	Orchidaceae	Bletia roezlii	28.408
2232	Orchidaceae	Bletia urbana	19.31
2233	Orchidaceae	Bletia urbana	16.254132
2234	Orchidaceae	Bletia urbana	19.329
2235	Orchidaceae	Bletia urbana	19.31
2236	Orchidaceae	Bletia urbana	19.367
2237	Orchidaceae	Bletia urbana	23
2238	Orchidaceae	Bletia tenuifolia	16.906
2239	Orchidaceae	Bletia tenuifolia	16.679
2240	Orchidaceae	Bletia tenuifolia	16.1
2241	Orchidaceae	Bletia tenuifolia	16.69
2242	Orchidaceae	Bletia tenuifolia	16.659
2243	Orchidaceae	Bletia tenuifolia	17.277
2244	Orchidaceae	Bletia tenuifolia	17.5538889
2245	Orchidaceae	Bletia nelsonii	16.917
2246	Orchidaceae	Bletia nelsonii	17.4
2247	Orchidaceae	Bletia concolor	19.457751
2248	Orchidaceae	Bletia concolor	18.4144
2249	Commelinaceae	Weldenia candida	16.133
2250	Commelinaceae	Weldenia candida	17.121
2251	Commelinaceae	Weldenia candida	19.782
2252	Commelinaceae	Weldenia candida	19.65
2253	Commelinaceae	Weldenia candida	19.283
2254	Commelinaceae	Weldenia candida	19.31666

2255	Commelinaceae	Weldenia candida	15.4272223
2256	Commelinaceae	Weldenia candida	19.01666
2257	Commelinaceae	Weldenia candida	15.53611
2258	Commelinaceae	Weldenia candida	14.75972
2259	Dioscoreaceae	Dioscorea palmeri	18.77
2260	Dioscoreaceae	Dioscorea palmeri	19.53
2261	Dioscoreaceae	Dioscorea palmeri	19.495
2262	Dioscoreaceae	Dioscorea palmeri	19.607
2263	Asparagaceae	Prochnyanthes mexicana	20.7333333
2264	Asparagaceae	Prochnyanthes mexicana	20.7774389
2265	Asparagaceae	Prochnyanthes mexicana	21.45527
2266	Asparagaceae	Prochnyanthes mexicana	22.41666
2267	Asparagaceae	Echeandia albiflora	18.12
2268	Asparagaceae	Echeandia atoyacana	17.2
2269	Asparagaceae	Echeandia breedlovei	16.9019444
2270	Asparagaceae	Echeandia breedlovei	16.68444
2271	Asparagaceae	Echeandia campechiana	19.07
2272	Asparagaceae	Echeandia coalcomanensis	18.793479
2273	Asparagaceae	Echeandia conzattii	17.325441
2274	Asparagaceae	Echeandia drepanoides	17.131176
2275	Asparagaceae	Echeandia drepanoides	17.018143
2276	Asparagaceae	Echeandia durangensis	25.03
2277	Asparagaceae	Echeandia durangensis	18.9633333
2278	Asparagaceae	Echeandia durangensis	25.1
2279	Asparagaceae	Echeandia echeandioides	19.2233334
2280	Asparagaceae	Echeandia echeandioides	17
2281	Asparagaceae	Echeandia echeandioides	19.667
2282	Asparagaceae	Echeandia echeandioides	19.361
2283	Asparagaceae	Echeandia echeandioides	19.183
2284	Asparagaceae	Echeandia echeandioides	18.683
2285	Asparagaceae	Echeandia echeandioides	21.15
2286	Asparagaceae	Echeandia echeandioides	17.97
2287	Asparagaceae	Echeandia echeandioides	18.84
2288	Asparagaceae	Echeandia elegans	17.713826
2289	Asparagaceae	Echeandia flavescens	21.809
2290	Asparagaceae	Echeandia flavescens	25.445
2291	Asparagaceae	Echeandia flavescens	19.903
2292	Asparagaceae	Echeandia flavescens	18.983
2293	Asparagaceae	Echeandia flavescens	20.642
2294	Asparagaceae	Echeandia flavescens	21.76
2295	Asparagaceae	Echeandia flavescens	25.01
2296	Asparagaceae	Echeandia flavescens	23.82
2297	Asparagaceae	Echeandia flavescens	25.06666
2298	Asparagaceae	Echeandia flavescens	19.5133333
2299	Asparagaceae	Echeandia flavescens	19.4802778

2300	Asparagaceae	Echeandia flavescens	19.1164444
2301	Asparagaceae	Echeandia flexuosa	20.99
2302	Asparagaceae	Echeandia flexuosa	21
2303	Asparagaceae	Echeandia flexuosa	22.57
2304	Asparagaceae	Echeandia flexuosa	23.767
2305	Asparagaceae	Echeandia flexuosa	20.72
2306	Asparagaceae	Echeandia flexuosa	18.937
2307	Asparagaceae	Echeandia flexuosa	20.202
2308	Asparagaceae	Echeandia flexuosa	19.017
2309	Asparagaceae	Echeandia flexuosa	16.933
2310	Asparagaceae	Echeandia flexuosa	17.599
2311	Asparagaceae	Echeandia formosa	16.5333333
2312	Asparagaceae	Echeandia formosa	16.2
2313	Asparagaceae	Echeandia gentryi	19.967
2314	Asparagaceae	Echeandia gracilis	19.34
2315	Asparagaceae	Echeandia gracilis	19.132
2316	Asparagaceae	Echeandia gracilis	19.308
2317	Asparagaceae	Echeandia grandiflora	16.448413
2318	Asparagaceae	Echeandia hallbergii	17.3
2319	Asparagaceae	Echeandia hallbergii	17.151053
2320	Asparagaceae	Echeandia hintonii	17.597317
2321	Asparagaceae	Echeandia hirticaulis	19.043
2322	Asparagaceae	Echeandia imbricata	18.499
2323	Asparagaceae	Echeandia imbricata	20.381
2324	Asparagaceae	Echeandia llanicola	17.443
2325	Asparagaceae	Echeandia longifolia	16.93
2326	Asparagaceae	Echeandia longipedicellata	19.733
2327	Asparagaceae	Echeandia longipedicellata	19.667
2328	Asparagaceae	Echeandia longipedicellata	22.933
2329	Asparagaceae	Echeandia longipedicellata	22.95
2330	Asparagaceae	Echeandia longipedicellata	19.375
2331	Asparagaceae	Echeandia luteola	19.6147222
2332	Asparagaceae	Echeandia luteola	20.08
2333	Asparagaceae	Echeandia luteola	20.55
2334	Asparagaceae	Echeandia luteola	21.44
2335	Asparagaceae	Echeandia luteola	20.64
2336	Asparagaceae	Echeandia luteola	20.701
2337	Asparagaceae	Echeandia luteola	20.459
2338	Asparagaceae	Echeandia magnifica	17.564
2339	Asparagaceae	Echeandia magnifica	17.564
2340	Asparagaceae	Echeandia mexiae	19.886952
2341	Asparagaceae	Echeandia mexiae	18.87642
2342	Asparagaceae	Echeandia mexicana	19.3216667
2343	Asparagaceae	Echeandia mexicana	19.1616667
2344	Asparagaceae	Echeandia mexicana	19.3166667

2345	Asparagaceae	Echeandia mexicana	19.87
2346	Asparagaceae	Echeandia mexicana	20.75
2347	Asparagaceae	Echeandia mexicana	19.1791667
2348	Asparagaceae	Echeandia mexicana	19.0430555
2349	Asparagaceae	Echeandia mexicana	18.69
2350	Asparagaceae	Echeandia mexicana	19.4977777
2351	Asparagaceae	Echeandia mexicana	19.1329445
2352	Asparagaceae	Echeandia mexicana	19.6102778
2353	Asparagaceae	Echeandia mexicana	19.49
2354	Asparagaceae	Echeandia mexicana	22.41666
2355	Asparagaceae	Echeandia mexicana	24.61
2356	Asparagaceae	Echeandia mexicana	24.55
2357	Asparagaceae	Echeandia mexicana	23.28
2358	Asparagaceae	Echeandia mexicana	28.407
2359	Asparagaceae	Echeandia mirandae	17.059094
2360	Asparagaceae	Echeandia montealbanensis	17.04555
2361	Asparagaceae	Echeandia nana	19.3236111
2362	Asparagaceae	Echeandia nana	19.3388889
2363	Asparagaceae	Echeandia nana	19.2730556
2364	Asparagaceae	Echeandia nana	19.72
2365	Asparagaceae	Echeandia nana	19.56
2366	Asparagaceae	Echeandia nana	19.6311111
2367	Asparagaceae	Echeandia nana	18.84
2368	Asparagaceae	Echeandia nana	22.05
2369	Asparagaceae	Echeandia nana	19.61666
2370	Asparagaceae	Echeandia oaxacana	16.960923
2371	Asparagaceae	Echeandia occidentalis	19.45
2372	Asparagaceae	Echeandia occidentalis	19.56
2373	Asparagaceae	Echeandia occidentalis	21.33
2374	Asparagaceae	Echeandia occidentalis	21.43222
2375	Asparagaceae	Echeandia occidentalis	19.745
2376	Asparagaceae	Echeandia occidentalis	20.723
2377	Asparagaceae	Echeandia occidentalis	21.865
2378	Asparagaceae	Echeandia occidentalis	20.95
2379	Asparagaceae	Echeandia occidentalis	22.25
2380	Asparagaceae	Echeandia occidentalis	21.47
2381	Asparagaceae	Echeandia occidentalis	22.078
2382	Asparagaceae	Echeandia palmeri	28.4
2383	Asparagaceae	Echeandia paniculata	19.2838889
2384	Asparagaceae	Echeandia paniculata	18.91
2385	Asparagaceae	Echeandia paniculata	23.966667
2386	Asparagaceae	Echeandia paniculata	17.501
2387	Asparagaceae	Echeandia paniculata	21.322
2388	Asparagaceae	Echeandia paniculata	19.013
2389	Asparagaceae	Echeandia paniculata	19.815

2390	Asparagaceae	Echeandia paniculata	20.088
2391	Asparagaceae	Echeandia paniculata	19.325
2392	Asparagaceae	Echeandia paniculata	18.987
2393	Asparagaceae	Echeandia parva	17.75
2394	Asparagaceae	Echeandia parva	17.839
2395	Asparagaceae	Echeandia parvicapsulata	19.32
2396	Asparagaceae	Echeandia parvicapsulata	19.86
2397	Asparagaceae	Echeandia parvicapsulata	19.237
2398	Asparagaceae	Echeandia parviflora	16.7972222
2399	Asparagaceae	Echeandia parviflora	16.8002778
2400	Asparagaceae	Echeandia parviflora	17.48
2401	Asparagaceae	Echeandia parviflora	16.8880555
2402	Asparagaceae	Echeandia parviflora	17.3
2403	Asparagaceae	Echeandia parviflora	18.983
2404	Asparagaceae	Echeandia parviflora	19.033
2405	Asparagaceae	Echeandia parviflora	19.556
2406	Asparagaceae	Echeandia parviflora	19.032
2407	Asparagaceae	Echeandia parviflora	18.345
2408	Asparagaceae	Echeandia parviflora	18.185
2409	Asparagaceae	Echeandia parviflora	18.469
2410	Asparagaceae	Echeandia parviflora	19.556
2411	Asparagaceae	Echeandia parviflora	18.272
2412	Asparagaceae	Echeandia parviflora	17.734
2413	Asparagaceae	Echeandia pseudoreflexa	16.16
2414	Asparagaceae	Echeandia ramosissima	22.33333
2415	Asparagaceae	Echeandia ramosissima	22.3
2416	Asparagaceae	Echeandia ramosissima	28.3033333
2417	Asparagaceae	Echeandia ramosissima	22.251
2418	Asparagaceae	Echeandia ramosissima	23.967
2419	Asparagaceae	Echeandia ramosissima	25.709
2420	Asparagaceae	Echeandia ramosissima	24.258
2421	Asparagaceae	Echeandia ramosissima	27.3
2422	Asparagaceae	Echeandia ramosissima	22.33
2423	Asparagaceae	Echeandia ramosissima	28.467
2424	Asparagaceae	Echeandia ramosissima	29.306
2425	Asparagaceae	Echeandia ramosissima	28.483
2426	Asparagaceae	Echeandia reflexa	18.9816667
2427	Asparagaceae	Echeandia reflexa	19.6
2428	Asparagaceae	Echeandia reflexa	19.3
2429	Asparagaceae	Echeandia reflexa	23.966667
2430	Asparagaceae	Echeandia reflexa	16.2
2431	Asparagaceae	Echeandia scabrella	21.04
2432	Asparagaceae	Echeandia scabrella	23.848
2433	Asparagaceae	Echeandia scabrella	21.323
2434	Asparagaceae	Echeandia scabrella	21.268

2435	Asparagaceae	Echeandia scabrella	20.67
2436	Asparagaceae	Echeandia scabrella	21.248
2437	Asparagaceae	Echeandia sinaloensis	19.517
2438	Asparagaceae	Echeandia skinneri	16.13
2439	Asparagaceae	Echeandia skinneri	16.85
2440	Asparagaceae	Echeandia skinneri	24.4
2441	Asparagaceae	Echeandia skinneri	15.993
2442	Asparagaceae	Echeandia skinneri	18.212
2443	Asparagaceae	Echeandia skinneri	18.263
2444	Asparagaceae	Echeandia skinneri	18.306
2445	Asparagaceae	Echeandia skinneri	19.589
2446	Asparagaceae	Echeandia skinneri	19.535
2447	Asparagaceae	Echeandia skinneri	20.335
2448	Asparagaceae	Echeandia skinneri	16.73
2449	Asparagaceae	Echeandia skinneri	23.91
2450	Asparagaceae	Echeandia skinneri	22.21
2451	Asparagaceae	Echeandia skinneri	19.13
2452	Asparagaceae	Echeandia skinneri	17.38
2453	Asparagaceae	Echeandia skinneri	19.09
2454	Asparagaceae	Echeandia skinneri	20.14
2455	Asparagaceae	Echeandia skinneri	19.02
2456	Asparagaceae	Echeandia smithi	17.259843
2457	Asparagaceae	Echeandia taxacana	17.226
2458	Asparagaceae	Echeandia tenuifolia	17.755
2459	Asparagaceae	Echeandia tenuis	17.584
2460	Asparagaceae	Echeandia tenuis	18.9
2461	Asparagaceae	Echeandia tenuis	19
2462	Asparagaceae	Echeandia udipratensis	19.956
2463	Asparagaceae	Echeandia udipratensis	19.913
2464	Asparagaceae	Echeandia vaginata	16.339443
2465	Asparagaceae	Echeandia vestita	19.5616667
2466	Asparagaceae	Echeandia vestita	17.58472
2467	Asparagaceae	Echeandia vestita	16.82
2468	Asparagaceae	Echeandia vestita	16.843
2469	Asparagaceae	Echeandia vestita	19.895
2470	Asparagaceae	Echeandia vestita	17.105
2471	Asparagaceae	Echeandia vestita	16.933
2472	Asparagaceae	Echeandia vestita	16.923
2473	Asparagaceae	Echeandia vestita	16.468
2474	Asparagaceae	Echeandia vestita	17.346
2475	Asparagaceae	Echeandia vestita	19.677
2476	Asparagaceae	Echeandia vestita	19.55
2477	Asparagaceae	Echeandia vestita	19.73
2478	Asparagaceae	Echeandia vestita	19.628
2479	Asparagaceae	Echeandia vestita	18.817

2480	Asparagaceae	Echeandia vestita	18.717
2481	Asparagaceae	Echeandia vestita	19.333
2482	Asparagaceae	Polianthes bicolor	17.6872
2483	Asparagaceae	Polianthes bicolor	17.670957
2484	Asparagaceae	Polianthes densiflora	27.739
2485	Asparagaceae	Polianthes densiflora	27.725
2486	Asparagaceae	Polianthes densiflora	27.274
2487	Asparagaceae	Polianthes durangensis	23.5
2488	Asparagaceae	Polianthes durangensis	23.416
2489	Asparagaceae	Polianthes durangensis	23.393
2490	Asparagaceae	Polianthes geminiflora	19.5916666
2491	Asparagaceae	Polianthes geminiflora	20.03
2492	Asparagaceae	Polianthes geminiflora	20.8
2493	Asparagaceae	Polianthes geminiflora	19.025
2494	Asparagaceae	Polianthes geminiflora	18.86
2495	Asparagaceae	Polianthes geminiflora	19.3133333
2496	Asparagaceae	Polianthes geminiflora	19.1135
2497	Asparagaceae	Polianthes geminiflora	18.79
2498	Asparagaceae	Polianthes geminiflora	19.5616667
2499	Asparagaceae	Polianthes geminiflora	17.93
2500	Asparagaceae	Polianthes geminiflora	22.5333333
2501	Asparagaceae	Polianthes tuberosa	19.354
2502	Asparagaceae	Polianthes tuberosa	18.736
2503	Asparagaceae	Polianthes tuberosa	18.624
2504	Asparagaceae	Polianthes tuberosa	18.6
2505	Asparagaceae	Polianthes tuberosa	19.5
2506	Asparagaceae	Polianthes tuberosa	18.673
2507	Asparagaceae	Polianthes tuberosa	18.453
2508	Asparagaceae	Polianthes tuberosa	17.05
2509	Asparagaceae	Polianthes tuberosa	19.056967
2510	Asparagaceae	Polianthes longiflora	19.516
2511	Asparagaceae	Polianthes longiflora	20.324
2512	Asparagaceae	Polianthes longiflora	19.652
2513	Asparagaceae	Polianthes longiflora	20.502
2514	Asparagaceae	Polianthes longiflora	20.438
2515	Asparagaceae	Polianthes longiflora	20.324
2516	Asparagaceae	Polianthes longiflora	20.502
2517	Asparagaceae	Polianthes longiflora	20.202
2518	Asparagaceae	Polianthes longiflora	20.9
2519	Asparagaceae	Polianthes longiflora	19.503
2520	Asparagaceae	Polianthes longiflora	20.698
2521	Asparagaceae	Polianthes longiflora	19.672
2522	Asparagaceae	Polianthes longiflora	19.4
2523	Asparagaceae	Polianthes longiflora	20.48
2524	Asparagaceae	Polianthes montana	21.347

2525	Asparagaceae	Polianthes montana	20.05
2526	Asparagaceae	Polianthes montana	19.983
2527	Asparagaceae	Polianthes montana	20.05
2528	Asparagaceae	Polianthes montana	19.367
2529	Asparagaceae	Polianthes montana	21.005
2530	Asparagaceae	Polianthes montana	21.455
2531	Asparagaceae	Polianthes montana	21.362
2532	Asparagaceae	Polianthes montana	20.043
2533	Asparagaceae	Polianthes montana	21.931
2534	Asparagaceae	Polianthes montana	22.491
2535	Asparagaceae	Polianthes montana	20.718
2536	Asparagaceae	Polianthes multicolor	21.462
2537	Asparagaceae	Polianthes nelsoni	23.91
2538	Asparagaceae	Polianthes nelsoni	23.86666
2539	Asparagaceae	Polianthes nelsoni	24.05
2540	Asparagaceae	Polianthes nelsoni	23
2541	Asparagaceae	Polianthes oaxacana	16.765016
2542	Asparagaceae	Polianthes oaxacana	16.734
2543	Asparagaceae	Polianthes palustris	22.224
2544	Asparagaceae	Polianthes palustris	20.806
2545	Asparagaceae	Polianthes palustris	20.912
2546	Asparagaceae	Polianthes palustris	22.311
2547	Asparagaceae	Polianthes palustris	20.942
2548	Asparagaceae	Polianthes palustris	22.345
2549	Asparagaceae	Polianthes palustris	23.917
2550	Asparagaceae	Polianthes palustris	22.365
2551	Asparagaceae	Polianthes palustris	20.973
2552	Asparagaceae	Polianthes palustris	21.917
2553	Asparagaceae	Polianthes palustris	22.267
2554	Asparagaceae	Polianthes palustris	21.835
2555	Asparagaceae	Polianthes palustris	21.25
2556	Asparagaceae	Polianthes palustris	23.5
2557	Asparagaceae	Polianthes palustris	18.926
2558	Asparagaceae	Polianthes palustris	24.041
2559	Asparagaceae	Polianthes palustris	23.967
2560	Asparagaceae	Polianthes platyphylla	22.31666
2561	Asparagaceae	Polianthes platyphylla	22.3042778
2562	Asparagaceae	Polianthes platyphylla	22.456
2563	Asparagaceae	Polianthes platyphylla	20.948
2564	Asparagaceae	Polianthes platyphylla	22.338
2565	Asparagaceae	Polianthes platyphylla	20.95
2566	Asparagaceae	Polianthes platyphylla	22.464
2567	Asparagaceae	Polianthes platyphylla	23.68
2568	Asparagaceae	Polianthes platyphylla	20.912
2569	Asparagaceae	Polianthes platyphylla	20.811

2570	Asparagaceae	Polianthes platyphylla	22.685
2571	Asparagaceae	Polianthes platyphylla	22.317
2572	Asparagaceae	Polianthes platyphylla	20.973
2573	Asparagaceae	Polianthes platyphylla	21.772
2574	Asparagaceae	Polianthes platyphylla	22.365
2575	Asparagaceae	Polianthes pringlei	21.33333
2576	Asparagaceae	Polianthes pringlei	22.5
2577	Asparagaceae	Polianthes pringlei	22.21666
2578	Asparagaceae	Polianthes pringlei	20.935
2579	Asparagaceae	Polianthes pringlei	23.911
2580	Asparagaceae	Polianthes sessiliflora	19.51
2581	Asparagaceae	Polianthes sessiliflora	20.907
2582	Asparagaceae	Polianthes sessiliflora	22.5
2583	Asparagaceae	Polianthes sessiliflora	22.913
2584	Asparagaceae	Polianthes sessiliflora	21.845
2585	Asparagaceae	Polianthes sessiliflora	21.517
2586	Asparagaceae	Polianthes sessiliflora	23
2587	Asparagaceae	Polianthes zapopanensis	20.8177778
2588	Asparagaceae	Polianthes michoacana	20
2589	Iridaceae	Nemastylis tenuis	28.39
2590	Iridaceae	Nemastylis tenuis	29.9833
2591	Iridaceae	Nemastylis tenuis	26.57
2592	Iridaceae	Nemastylis tenuis	28.66666
2593	Iridaceae	Nemastylis tenuis	24.05
2594	Iridaceae	Nemastylis tenuis	24.0333337
2595	Iridaceae	Nemastylis tenuis	23.96
2596	Iridaceae	Nemastylis tenuis	25.08333
2597	Iridaceae	Nemastylis tenuis	20.81
2598	Iridaceae	Nemastylis tenuis	19.5786111
2599	Iridaceae	Nemastylis tenuis	18.81666
2600	Iridaceae	Nemastylis tenuis	19.5108333
2601	Iridaceae	Nemastylis tenuis	16.93
2602	Iridaceae	Nemastylis tenuis	22.21666
2603	Iridaceae	Nemastylis tenuis	23.74
2604	Iridaceae	Nemastylis tenuis	28.269
2605	Iridaceae	Nemastylis tenuis	19.346
2606	Iridaceae	Nemastylis tenuis	17.121
2607	Iridaceae	Nemastylis tenuis	30.951
2608	Iridaceae	Nemastylis tenuis	28.24
2609	Iridaceae	Nemastylis tenuis	23.756
2610	Iridaceae	Nemastylis tenuis	17.121
2611	Iridaceae	Nemastylis tenuis	20.967
2612	Iridaceae	Nemastylis tenuis	28.413
2613	Iridaceae	Nemastylis tenuis	18.952
2614	Iridaceae	Nemastylis tenuis	27.972

2615	Iridaceae	Nemastylis tenuis	23.938
2616	Iridaceae	Nemastylis tenuis	21.2
2617	Iridaceae	Nemastylis tenuis	28.008
2618	Iridaceae	Nemastylis tenuis	23.983
2619	Iridaceae	Nemastylis tenuis	23.883
2620	Iridaceae	Nemastylis tenuis	18.967
2621	Iridaceae	Nemastylis tenuis	21.133
2622	Iridaceae	Nemastylis tenuis	22.45
2623	Iridaceae	Nemastylis tenuis	24.517
2624	Iridaceae	Nemastylis tenuis	28.239
2625	Iridaceae	Nemastylis tenuis	23.483
2626	Iridaceae	Nemastylis tenuis	23.283
2627	Iridaceae	Nemastylis tenuis	19.452
2628	Iridaceae	Nemastylis tenuis	23.392
2629	Iridaceae	Nemastylis tenuis	16.93
2630	Iridaceae	Nemastylis tenuis	20.083
2631	Iridaceae	Nemastylis tenuis	23.87
2632	Iridaceae	Nemastylis tenuis	23.401
2633	Iridaceae	Nemastylis tenuis	19.827
2634	Iridaceae	Nemastylis tenuis	23.43
2635	Iridaceae	Nemastylis tenuis	27.867
2636	Iridaceae	Nemastylis tenuis	29.98
2637	Iridaceae	Nemastylis tenuis	18.91
2638	Iridaceae	Nemastylis tenuis	23
2639	Orchidaceae	Triphora debilis	16.43
2640	Orchidaceae	Triphora debilis	16.12
2641	Orchidaceae	Triphora debilis	16.89
2642	Orchidaceae	Triphora gentianoides	18.227
2643	Orchidaceae	Triphora gentianoides	16.698
2644	Orchidaceae	Triphora gentianoides	17.35
2645	Orchidaceae	Triphora gentianoides	17.267
2646	Orchidaceae	Triphora gentianoides	20.66

long.
-106.7466667
-97.20583333
-93.14083333
-97.1625
-98.77861111
-98.74083333
-97.30972222
-98.74138889
-97.265
-98.91222222
-97.17861111
-107.775
-107.3166667
-107.6416667
-98.25805556
-101.76
-103.105
-102.5366667
-103.5433333
-99.246059
-101.3433333
-104.0377778
-100.66
-103.893291
-102.6225
-100.3580417
-98.48944444
-96.939926
-96.97443
-96.89638889
-96.954334
-96.726102
-96.99333333
-96.990824
-102.2725
-98.75583333
-96.9725
-97.18805556
-104.6447222
-98.50583333
-98.500125
-98.57833056
-96.865586

-98.86664167
-96.65193889
-98.52671944
-102.3333333
-98.52611111
-91.64278056
-98.23483333
-96.3725
-97
-104
-96.45583333
-98.43333333
-99.84801667
-99.85833333
-99.92583333
-98.7825
-103.6833333
-100.5
-99.1
-100.45
-101.733
-104.083
-99.342
-97.285199
-92.655677
-92.613888
-92.336072
-97.500138
-97.520247
-101.3082028
-106.19915
-101.3666667
-102.4452778
-99.754144
-104.47036
-107.735859
-115.410256
-115.604797
-115.459844
-115.432658
-113.35
-114.85
-112.8558333
-101.056123
-115.8333333

-115.9
-100.865
-100.4744444
-103.8358333
-100.81
-102.5705556
-91.39027778
-97.992037
-101.3430556
-100.4558333
-97.9725
-95.323753
-97.50307
-96.870347
-96.72361111
-97.08916667
-98.72
-97.10583333
-100.9725
-102.5058333
-98.75583333
-97.48916667
-97.00583333
-99.68916667
-97.33916667
-99.80583333
-99.3725
-100.9725
-91.8725
-97.28055556
-98.74083333
-98.82277778
-97.34805556
-97.2025
-97.30166667
-97.26027778
-97.00666667
-98.72638889
-98.74555556
-97.06944444
-98.91472222
-97.45055556
-97.19722222
-97.28055556
-97.1275

-98.69166667
-97.31916667
-98.88333333
-98.77861111
-98.87083333
-98.80638889
-96.28208333
-97.99861111
-104
-107.6833333
-97.39777778
-99.011
-102.4391306
-99.69166944
-98.52612778
-98.42830833
-99.68333333
-103.4216667
-101.9400611
-98.16278056
-98.88528056
-97.13028056
-101.97
-100.3616667
-96.52083333
-102.8283333
-97.26333333
-97.09
-104.1516667
-103.2233333
-97.07861111
-101.2833333
-102.9816667
-102.6083333
-97.115
-97.615
-97.36
-103.4827778
-98.11833333
-91.91944444
-103.345
-96.32
-102.3583333
-107.9166667
-97.26361111

-100.33
-97.04666667
-101.5858333
-100.0566667
-99.03166667
-98.45
-100.1933333
-99.94444444
-100.745
-98.24333333
-107.0736111
-97.56666667
-109.838607
-96.35583333
-96.28916667
-96.45583333
-96.8725
-96.7225
-115.3666667
-107.8366667
-113.39
-113.88
-113.39
-113.88
-112.9225
-115.542808
-113.9316667
-115.507649
-115.4
-115.432658
-98.074
-99.85833333
-99.7325
-98.25805556
-103.34
-107.77545
-104.644855
-99.271719
-100.297466
-115.44
-114.8069444
-108.0416667
-108.0416667
-100.97
-115.3891667

-115.3166667
-97.155054
-101.955
-97.27965833
-106.4777778
-102.0766667
-99.78916667
-97.26027778
-97.83222222
-96.47693889
-98.712789
-96.938667
-97.417453
-90.96817
-99.429681
-96.389837
-91.83916667
-88.307335
-90.88916667
-103.685
-94.98916667
-88.62888889
-87.51277778
-111.850457
-99.829525
-100.933205
-99.724251
-102.819641
-96.490769
-115.921199
-105.814662
-100.899933
-101.0442
-101.833411
-97.463967
-99.189045
-97.783673
-99.60583333
-96.7575
-99.48916667
-100.4058333
-100.1558333
-97.73916667
-98.28166667
-97.19

-97.25583333
-96.949655
-98.80638889
-99.98053333
-99.05618333
-101.3833333
-100.77
-98.52612778
-111.4
-106.4077167
-91.3686
-96.53636667
-103.437875
-107.0730556
-96.11888889
-101.5858333
-97.285
-102.9833333
-88.51944444
-111.4
-92.625
-108.3333
-103
-104.6666667
-100.03333
-100.36666
-99.99
-97.685
-100.98
-101.9075
-99.84833333
-100.4066667
-103.105
-97.43333
-99.544673
-102.5127778
-103.1225
-92.60614
-98.99111111
-99.043487
-98.99111111
-96.15918
-99
-97.02972222
-97.03166667

-98.05583333
-98.16
-100.8055556
-95.80583333
-97.18166667
-98.9
-98.86666667
-96.83916667
-99.10583333
-103.0283333
-102.02
-100.6166667
-103.85
-100.3833333
-101.775
-93.57
-96.8225
-96.312988
-91.88916667
-104.62
-87.471755
-101.3791583
-88.142516
-89.70972222
-89.78888889
-89.07333333
-107.3891667
-106.1083333
-107.5194389
-102.5958306
-103.6305556
-103.105
-102.6216667
-102.745
-107.2166667
-108.4455306
-107.0819444
-109.1944444
-97.011577
-103.0635472
-102.3761111
-101.525
-97.10583333
-96.895
-96.769433

-98.13916667
-92.73916667
-96.20583333
-103.7494444
-98.73
-98.73972222
-97.40666667
-97.06944444
-98.75277778
-97.17861111
-96.45
-99.57780278
-105.2619611
-104.4666667
-98.63
-103.7162
-98.77
-100.545
-96.52083333
-101.275
-98.02
-98.984197
-96.891394
-98.99825833
-98.082885
-96.89638889
-101.7225
-99.69166944
-99.65166667
-97.516936
-96.30583333
-98.572501
-96.938667
-95.01388
-101.907671
-97.3025
-97.42777778
-98.561874
-98.73
-98.90028056
-100.39
-96.651417
-96.938667
-96.938667
-97.164429

-98.58333333
-100.8666667
-99.810537
-100.618387
-97.473201
-97.43333333
-96.1725
-88.37833333
-91.70722222
-88.176983
-89.61
-102.497843
-99.19973
-98.764798
-98.18278056
-99.79055556
-99.300381
-98.982744
-98.16
-98.980719
-97.5333
-99.250228
-99.189115
-116.7333333
-115.1058333
-111.920772
-115.7166667
-116
-115.3333333
-115.1333333
-111.85
-111.9708333
-111.5994972
-114.288159
-112.25
-111.5791667
-113.5013889
-115.4833333
-118.270847
-96.9917
-96.64488
-93.66305833
-93.46949722
-99.133316
-98.884492

-97.91990833
-98.78277778
-97.98972222
-96.387163
-96.6833
-93.15972222
-92.60666667
-99.87
-103.695
-103.6725
-99.84416667
-98.13138889
-100.661397
-105.138067
-99.635336
-99.9
-103.930423
-97.090339
-96.64379
-87.63089444
-89.88055556
-90.02756111
-100.3994389
-101.2125
-98.99111111
-89.83333333
-93.11083333
-87.58916667
-88.35583333
-95.028104
-102.4891667
-90.43916667
-88.307335
-93.51189722
-102.57
-103.1
-103.105
-98.98333333
-88.08333333
-90.11666667
-89.68638889
-87.825
-88.04444444
-96.393122
-94.95138889

-96.834471
-99.97166667
-98.97583056
-98.525525
-95.86666667
-98.52666667
-96.753573
-95.30583333
-91.05583333
-100.942609
-96.856106
-96.83027778
-95.71666667
-95.79333333
-104.7582444
-99.95222778
-99.97166667
-103.9680556
-96.72138889
-98.74444444
-97.30805556
-97.13806111
-103.2333333
-99.83874167
-99.86834722
-100.77
-100.5716667
-107.6725
-97.65333056
-102.684275
-100.422675
-102.9783333
-98.73166667
-97.30805556
-103.25
-106.9891667
-108.8916667
-103.893794
-103.8166667
-104.9433333
-96.80583333
-96.87138889
-99.88916667
-98.74861111
-98.84583056

-97.37166667
-98.81194444
-95.92
-91.57527778
-90.26361111
-96.75583333
-95.48472222
-90.28833333
-97.015
-98.49916667
-98.46237222
-99.66128
-99.71666667
-96.56666667
-98.23888889
-100.0913889
-101.5706611
-98.98333333
-96.31666667
-99.31333333
-96.938667
-96.835495
-96.981799
-98.85583333
-98.78916667
-95.725
-98.35436944
-107.3028361
-107.5194389
-99.88333333
-98.5167
-97.26833333
-103.3566667
-98.13166667
-103.1
-99.08
-91.33333333
-100.6225
-97.25583333
-97.03166667
-97.20942
-98.36666667
-102.4397222
-99.35833333
-101.3333333

-96.28916667
-96.4225
-99.5
-92.63
-104.7166667
-102.2725
-104.64
-103.6725
-105.8583333
-100.654491
-96.89638889
-95.46437778
-98.16
-100.2183333
-91.28916667
-98.86222222
-95.44
-101.140461
-96.28208333
-96.14647778
-96.07083333
-96.21333333
-97.55
-98.3333
-100.0764
-92.545114
-96.685456
-96.41972222
-97.26916667
-97.32833333
-98.15
-98.7
-100.3991667
-103.6558333
-97.00166667
-97.11833333
-99.8
-99.87
-97.50583333
-104.0297194
-102.6401917
-103.4066667
-102.5
-95.26921667
-98.90472222

-103.7105556
-102.2225
-100.4725
-97.28916667
-99.80583333
-98.70777778
-98.74555556
-98.75
-99.98445278
-101.4
-98.77
-95.49129167
-99.39166667
-103.4067333
-103.7263889
-104.333333
-101.610395
-97.396917
-99.652466
-95.495
-103.5
-103.7833333
-103.4408333
-98.91666667
-98.9
-97.10583333
-97.00583333
-98.05583333
-99.33916667
-97.20083333
-97.41083333
-96.33666667
-102.4083333
-90.01666667
-97.30691667
-99.93333333
-100.965
-100.020188
-96.855831
-93.12444444
-91.70583333
-91.21833333
-93.31666667
-92.473
-93.6

-93.211
-96.961478
-100.95
-102.775
-99.46075
-98.14630556
-98.21221944
-98.941711
-99.24011111
-99.81583056
-98.81833333
-96.663414
-96.5225
-96.4225
-98.10043611
-97.41333333
-99.095
-98.86222222
-99.31
-96.32
-98.42833333
-100.3983333
-97.2625
-96.663566
-96.86278056
-91.76145833
-98.10113333
-95.77778056
-107.3299694
-100.3927861
-92.51
-97.233333
-101.4833333
-98.00611111
-95.0225
-98.25833333
-99.296193
-91.71555556
-91.62156944
-99.67748056
-98.08
-103.16
-97.129535
-96.949122
-100.8666667

-92.19131
-101.9058333
-96.975729
-96.848826
-96.898547
-96.15583333
-98.23916667
-91.54462778
-95.5
-98.35656389
-101.9303056
-100.2766667
-98.125
-96.6225
-96.10583333
-96.80583333
-96.3725
-95.0225
-96.89638889
-96.20583333
-95.86583056
-96.36833333
-99.14666667
-99.65166667
-103.167795
-97.5333
-96.47778
-99.3
-97.21667
-104.26389
-98.652
-100.433
-105.72
-98.52666667
-99.51666
-98.84583333
-104.343729
-95.68916667
-98.86444444
-92.678268
-97.3333
-100.3466667
-98.33333333
-102.5058333
-103.7361111

-97
-99.55583333
-97.08916667
-97.94416667
-96.738697
-101.7558333
-98.32778333
-95.0675
-98.48083056
-99.83333333
-99.16166667
-98.81861111
-91.05833333
-98.86666667
-96.2725
-99.46075
-97.1981
-96.31222
-110.95
-98.983
-101.15
-100.15
-104.264
-98.11389
-98.888
-101.7333333
-100.267
-109.967
-104.26944
-109.108
-109.008
-108.9264
-96.462119
-92.5
-99.733333
-99.99
-102.4452778
-101.5244444
-92.116
-99.32
-97.247225
-99.98
-99.25
-99.88166667
-92.7779

-99.18333
-103.15
-97.2264
-97.48916667
-96.848826
-96.72722222
-96.876146
-96.89638889
-102.1391667
-100.3558333
-102.7725
-98.74555556
-97.14583333
-98.77861111
-108.7472222
-98.47523056
-107.5194444
-104.6793944
-103.7163778
-103.1026111
-97.47318056
-98.91638889
-109.794785
-106.3666667
-99.15583333
-93.35444444
-98.36666667
-103.1777778
-95.6725
-93.66333333
-92.84
-103.7
-99.03333
-99.81666
-103.36666
-99.1825
-101.53333
-99.15
-104.93333
-92.77
-99.15
-101.97
-99.1825
-102.78
-94.03

-96.43333
-99.9
-99.333
-100.985
-103.507
-97.544
-100.154
-97.31667
-103.75
-101.8
-109.983
-99.73
-100.5214722
-108.75
-108.66667
-99.083
-103.245
-100.271732
-114.783867
-99.23
-102.058
-101.602
-96.4
-96.383
-102.258
-101.528
-98.732
-99.267
-99.893
-99.083
-104.9944444
-97.163
-92.543
-98.67
-100.152
-99.321
-101.279
-96.818
-104.752
-103.2
-99.504
-104.78
-105.962
-107.635
-96.48333

-92.183
-102.25
-99.318
-103.37
-99.15
-106.379
-105.81667
-94.19167
-93.01
-99.899329
-99.775875
-92.97
-97.133
-96.31083333
-99.083
-99.2
-97.167
-103.437
-102.03
-104.495
-104.33
-105.837
-105.6
-108.723
-101.194
-102.414
-104.3
-97.151
-99.267
-98.68166667
-99.2172222
-97.9517
-92.339
-99.32
-99.14833
-92.76
-92.57
-98.05
-102.072
-97.13
-101.457
-99.117
-99.74194444
-95.06667
-103.580249

-89.23444
-96.5
-92.82
-99.53
-103.636546
-101.8
-99.098
-98.87
-97.277
-97.430693
-100.933
-99.238
-99.46666667
-99.742
-107.635
-96.10139
-92.09
-96.09306
-94.031
-99.5
-93.393
-91.83
-103.15
-98.983
-94.34833
-104.313
-97.287
-88.93
-105.05
-99.067
-99.473
-110.05
-108.207
-108.767
-95.332
-98.95
-89.52833
-101.415
-104.73333
-94.117
-96.872
-94.133
-93
-105.3
-108.8

-93.717
-96.45
-103.283
-99.27
-96.13333
-100.05
-99.14
-98.11389
-101.112
-99.632
-100.38333
-99.217
-99.74194444
-108.28333
-95.5833
-93.5
-99.3
-98.1125
-104.597
-100.7
-107.5
-99.275
-102.5666667
-98
-99.191
-96.03
-97.5667
-96.31083333
-100.27
-102.264
-99.08
-99.267
-97.21667
-110.033
-99.74194444
-109.025
-108.20833
-96.284583
-106
-105.77
-97.067
-99.154
-99.3
-104.63379
-106

-96.06667
-96.07
-99.23
-99.217
-99.37
-91.9
-108.258498
-96.4
-93.404
-96.94583
-97.41667
-99.203
-99.08
-100.39167
-96.10167
-94.5
-93.612
-89.41028
-97.51667
-99.053
-100.5
-98.715
-94.683
-94.517
-99.48
-101.133
-99.258
-99.388
-98.52
-104.05
-108.88722
-98.67
-97.87
-97.003
-97.2
-101.447
-100.152
-97.367
-102.718
-99.24
-98.74
-98.693
-103.172
-100.798
-100.033

-103.222
-102.228
-105.802
-104.133
-103.78
-108.274
-99.239
-100.453
-99.237
-116.667
-100.084
-99.308
-104.6
-102
-107.5688333
-100.81
-100.17
-99.1
-103.465
-98.517
-99.75
-105.75
-107.3543333
-92.5
-99.19
-102
-103.392
-104.593
-103.222
-102.343
-99.74194444
-100
-99.08
-100.718
-99.65055556
-100.13
-98.517
-102
-99.1
-99.645
-99.88
-99.23
-104.325
-102.475
-100.827

-103.417
-104.265
-104.268
-97.97722222
-103.34
-103.018
-100.04
-104.95
-99.55
-108.713
-108.70278
-116.13
-116.47
-100.05
-104.786
-104.133
-105.32037
-105.3
-108.65
-115.25
-115.883
-116.75
-109.95
-89.4894444
-93.4844444
-96.35
-87.185
-92.96
-95.35
-87.6313889
-103.69
-98.63
-103.34
-99.87
-100.94
-99.23
-104.46
-100.16
-99.5
-105.86
-99.15
-97.14
-103.7833333
-103.6666667
-103.9333333

-101.25
-104.0483333
-104.4341667
-103.2702778
-104.2833333
-103.4666667
-103.4666667
-100.45
-101.975
-99.16666667
-104.92
-104.9
-97.90888889
-110.2833333
-110.2166667
-100.58
-100.8983333
-99.065
-99.925
-97.787
-97.78667
-109.65028
-102.657
-102.65667
-99.025
-99.025276
-100.70417
-100.704
-99.867
-99.86833
-99.868
-100.05
-100.05
-98.096664
-98.097
-103.085
-99.533
-99.53333
-99.48861
-99.243614
-99.875
-99.875
-99.883
-109.988
-99.682

-99.68166
-97.62
-101.043
-101.043335
-98.8725
-99.07278
-99.068054
-103.34
-103.16
-99.462
-99.46167
-101.67667
-101.677
-100.169
-100.186
-100.762
-100.761665
-103.48
-105.802
-105.836
-99.18
-99.155
-99.155
-99.155
-99.19583
-99.19361
-102.394
-99.23
-99.23
-99.267
-99.233
-99.267
-99.25
-106.835
-106.49834
-106.498
-100.35167
-100.352
-104.85
-104.833
-105.03
-104.994
-105.01
-104.67
-104.982

-104.98167
-99.10528
-99.65361
-99.654
-101.683
-101.707
-101.706665
-101.736664
-101.737
-99.94
-102.885
-102.391
-102.348
-99.772
-99.77167
-103.465
-103.465
-101.442
-101.18833
-99.12222
-99.129166
-101.455
-101.428
-101.42834
-101.42834
-99.20333
-99.203
-99.21528
-103.2
-103.2
-99.41139
-98.888054
-99.248
-99.24778
-98.775
-98.775
-103.139
-103.124
-103.101
-102.71833
-102.718
-102.718
-99.17333
-99.173
-103.417

-103.467
-103.376
-103.43667
-103.437
-103.437
-103.23
-103.232
-103.242
-103.237
-103.233
-103.222
-103.251
-103.212
-98.28
-101.817
-101.935
-101.935
-101.68
-108.274
-99.248886
-97.267
-104.002
-103.01833
-104.632
-104.63167
-104.617
-96.3
-96.29
-98.6975
-98.697
-101.10667
-101.279
-101.255
-101.515
-108.542
-103.168
-103.281
-99.23805
-104.7
-99.31416
-97.2
-104.317
-99.25
-93.39
-98.73333

-98.788
-98.708
-103.28333
-103.283
-101.6
-101.52167
-99.813
-99.81333
-99.633
-101.476
-105.31333
-105.033
-100.39167
-100.392
-109.12972
-103.236
-100.867
-100.751
-101.48972
-101.49
-101.297
-101.29667
-101.023
-101.02333
-103.71
-106.433
-102.41417
-100.385
-100.385
-100.388
-96.965
-97.003
-99.983
-100.508
-100.49389
-100.44028
-100.44
-101.046
-100.978615
-100.979
-103.933
-97.061386
-97.683
-97.645
-110.614

-110.38279
-110.36667
-97.02
-97.02
-97.1
-105.883
-105.89167
-105.892
-101.471664
-101.472
-103.767
-104.1
-104.197
-104.179
-104.125
-104.3
-104.3
-103.923
-103.92306
-104.1
-102.25667
-102.346
-103.75
-103.758
-103.30666
-103.307
-99.67
-100.07
-99.574
-98.768
-98.55
-98.55417
-98.549446
-98.58139
-98.83583
-99.30389
-99.24084
-99.25472
-99.25472
-99.042
-98.86833
-98.918335
-98.8175
-98.833336
-98.833336

-98.967
-98.99333
-103.561
-99.14
-99.18
-99.2
-99.14833
-99.22139
-99.056
-99.054
-102.013336
-98.95944
-99.92889
-99.929
-103.55
-103.55
-99.17
-99.20194
-107.935
-102.094
-103.983
-103.98333
-100.2
-100.262
-100.261665
-96.801
-102.843
-99.135
-109.033
-109.033
-108.933
-108.908
-103.483
-100.603
-100.614
-97.6
-97.6
-98.652
-98.65889
-98.74555
-97.06
-100.827
-100.82694
-100.47
-97.84

-97.45
-100.51
-97.7
-98.64
-99.23
-98.74
-104.07
-101.17
-104.99
-104.99
-104.9
-105.0
-108.71667
-108.717
-108.717
-108.68889
-108.689
-108.689
-109.025
-108.911835
-106.47
-107.684
-107.684
-108.33
-105.4366667
-102.4441667
-99.55416667
-99.54
-97.51666667
-94.33666667
-97.6
-99.76666667
-116.6
-102.04444
-101.38
-99.616113
-98.435925
-98.119575
-98.467919
-96.4
-93.47
-96.784
-99.095
-99.47
-99.2

-92.3419444
-96.59
-93.1741667
-92.87
-99.491
-102.85222
-100
-105.95
-100.05
-100.36666
-99.155
-92.4436111
-99.61
-99.09
-99.733333
-100.61
-105.83
-96.94333
-103.283
-104.203
-100.588
-99.564
-98.706
-99
-108.2222
-92.8344444
-99.46666
-96.4
-96.784
-99.095
-99.2
-99.473
-97.062495
-100.15
-97.1
-102.045
-97.838
-103.84
-99
-98.687
-92.231641
-92.56
-99.84972
-103.5666667
-100.466667

-101.86666
-99.28
-97.6111111
-96.79
-97.01667
-99.98889
-99.564
-104.133
-103.84
-95.878
-99.76611
-99.7525
-98.67028
-99.238
-99.32
-102.067
-102.052
-100.04
-103.39
-103.34
-103.43
-106.427
-105.84
-106.379
-99.982
-103.35355
-100.368789
-95.850751
-99.467627
-103.577801
-103.445626
-99.105887
-99.475562
-103.396959
-99.680637
-67.62
-64.435
-68.66
-82.63
-84.224722
-84.092222
-86.37
-86.33
-89.29
-86.35

-86.03
-89.68
-88.18
-92.092
-96.53778
-96.30028
-94.13333
-94.183
-92.848
-92.04861
-92.374
-101.163
-99.35
-99.267
-102.023
-102.052
-102.43
-101.267
-101.68
-101.307
-101.255
-101.655
-99.185
-99.181
-99.78
-96.75
-103.6667
-104.896
-103.223
-103.417
-105.355
-100.05187
-101.298283
-99.921286
-96.51755
-99.055565
-98.841565
-86.500588
-105.287941
-90.246852
-104.712047
-101.46262
-86.17851
-104.968227
-74.205982

-89.289971
-93.057891
-98.591184
-101.601651
-103.461862
-91.774219
-100.689855
-87.079062
-101.0167
-97.01317
-101.607413
-99.534082
-102.073261
-96.877216
-92.459411
-99.176734
-91.995548
-67.588536
-96.696739
-98.721691
-92.107001
-97.146132
-99.189866
-99.171137
-76.569696
-103.226654
-103.618735
-87.34565
-87.55248
-102.359006
-100.051994
-78.411542
-88.450397
-98.356617
-100.359893
-104.123049
-103.470023
-99.255351
-101.092731
-98.746267
-99.197099
-99.747756
-99.100067
-95.01556
-99.717

-99.7525
-99.35
-99.726
-102.08056
-100.47
-99.25
-99.1
-102.052
-100.04
-99.78
-103.467
-103.34
-103.433
-109.887
-104.311162
-104.122202
-103.569948
-106.229522
-106.495573
-104.881372
-103.585396
-104.830641
-103.530054
-102.545909
-97.26666
-104.697
-105.06444
-103.78
-104.632
-105.843
-104.19722
-102
-100.713804
-104.917074
-104.668757
-99.618623
-100.196222
-100.081085
-100.343447
-99.723454
-101.457573
-102.009718
-97.288224
-96.959414
-99.112429

-103.326722
-104.89816
-99.331387
-96.95
-98.67
-101.191
-100.132
-99.345
-99.233
-99.53972
-98.967
-102.058
-101.19
-100.077
-104.896
-112.238
-108.6767
-108.717
-108.75
-108.66667
-109.3167
-108.5
-108.7233
-99.995558
-96.734016
-96.722173
-96.08944
-96.7
-96.856
-99.875
-99.893
-99.6
-99.23
-99.9
-105.3
-103.59645
-103.461662
-103.334569
-98.594791
-99.668094
-100.182937
-103.020328
-99.941205
-104.488159
-103.468054

-96.507
-100.27
-99.283
-99.333
-99.88
-98.7
-102.052
-101.788
-101.852
-99.178
-103.433
-103.767
-103.223
-104.65
-105.355
-105.83
-106.379
-105.88333
-99.5
-102.071
-100.04
-99.093
-99.117
-97.57639
-101.743
-101.733
-99.564
-98.65
-99.156
-99.567
-98.765204
-98.767899
-99.188136
-100.059162
-100.757252
-99.287048
-98.797447
-101.254513
-100.162134
-99.967013
-99.270328
-99.154753
-98.4833
-97.247221
-99.137153

-104.075577
-100.104491
-104.876002
-99.088733
-99.96639
-99.2994
-97.733965
-98.33358
-96.690089
-99.295836
-99.345248
-99.565406
-97.356722
-102.25
-92.04
-99.453
-99.567
-99.6
-98.167
-97.88333
-97.68
-96.94778
-99.67
-99.117
-103.788
-100.322
-96.667
-96.747
-96.698
-99.517
-99.879
-101.975542
-96.735001
-100.046857
-103.858096
-103.438
-103.470911
-103.18676
-102.167532
-101.157112
-99.282477
-96.734016
-96.438664
-103.335045
-96.53333

-99.76444
-97.85
-96.70833
-99.238
-99.333
-103.083
-102.005
-101.788
-100.04
-99.143
-103.22278
-100.295747
-92.3238
-103.142732
-101.62299
-99.32171
-107.572896
-99.969329
-80.97
-83.65
-86.33
-89.29
-96.4
-98.05
-94.6
-101.653
-100.15
-99.3
-99.6
-99.309
-99
-97.087
-92.41056
-103.37
-102.05
-102.217
-101.26194
-101.59797
-100.115913
-100.046202
-96.983
-88.09
-103.432
-104.956
-99.32

-104.33
-110.085
-109.933
-105.824
-111.458
-113.103
-86.698769
-90.297544
-91.471798
-88.710645
-83.011631
-83.618791
-83.155096
-90.344534
-90.120779
-79.556691
-87.846817
-75.69303
-89.549727
-89.190372
-87.850111
-90.111912
-79.396219
-90.675732
-91.850928
-90.331406
-87.80512
-88.046014
-77.308865
-78.464286
-87.802078
-93.078487
-91.701553
-99.674945
-99.332736
-99.141416
-94.833736
-98.641754
-97.956876
-97.844523
-100.034425
-99.260629
-95.35876
-97.712482
-96.967043

-93.719851
-92.013486
-92.957523
-98.84784
-100.128028
-99.615372
-98.707798
-98.623208
-105.250031
-103.672485
-103.381395
-99.307909
-99.344582
-102.300418
-98.68333333
-95.034248
-96.259873
-96.491562
-97.752339
-106.372354
-97.956876
-79.561436
-85.93333
-99.792682
-88.395509
-86.872461
-88.049065
-88.872254
-88.879684
-99.304681
-92.82277
-99.083
-90.37
-97.115074
-95.065177
-96.963359
-86.184926
-86.199639
-79.563031
-90.675732
-85.93333
-85.52
-99.67
-79.115366
-81.0176

-87.83
-89.579703
-86.03333333
-83.333334
-83.342222
-83.481944
-82.62
-80.86
-79.53
-84.25
-84.186111
-82.96797
-79.56
-85.502778
-85.611389
-84.83
-84.44
-86.31
-85.59
-85.9
-86.45
-84.72
-87.83
-86.2
-96.467
-92.792
-92.581
-92.192
-91.989
-90.25
-95.067
-96.01556
-94.11666667
-93.888
-92.3
-92.52
-92.898
-91.66667
-91.03333333
-89.03
-89.89
-99.683
-97.83333333
-96.5
-94.6

-93.05
-93.58333333
-92.116
-92.97
-92.45
-91.133
-91.8
-89.40472
-88.88333333
-88.5
-103.4
-101.605
-99.67
-99.31666667
-99.6
-97.087
-97.011
-96.00028
-95.083
-95.377
-95.07
-94.885
-94.34833
-93.23333333
-92.433
-92.58333333
-92.38444
-92.091
-91.052
-90.78333333
-89.45
-89.28333333
-88.02
-104.437
-98.88333333
-97.166
-97.7
-97.42
-96.8
-96.25
-96.592
-96.764
-96.38333333
-96.823
-88.03

-105.25
-105.315
-98.267
-98.21666667
-98.22
-86.89205
-103.172
-100.0166667
-99.26666667
-99.329
-99.55
-97.75
-104.67
-99.4
-97.8
-97.861
-99.495
-108.6667
-96.442
-96.592
-99.55823
-99.120221
-96.735001
-104.738307
-101.455883
-99.444658
-99.557286
-96.9798
-103.757144
-103.565308
-99.936259
-100.327269
-103.021292
-99.19
-99.558854
-104.566347
-86.35
-90.82
-86.6
-90.02
-89.68
-87.1
-96.06611
-94.183
-97.293

-101.808
-100.017
-100.151
-99.333
-99.32
-99.965
-99.32
-97.32
-101.6
-100.462
-100.062
-103.289
-103.438
-103.537
-103.222
-99.504
-104.517
-106.427
-106.379
-107.633
-108.7
-108.717
-99.19
-96.302787
-99.185
-99.19
-99.155
-102
-92.056
-91.069
-91.45
-91.08
-90.816
-92.315
-92.16666667
-94.683
-95.967
-102.059338
-101.3144
-96.3
-96.003
-100.658
-100.272
-98.817
-98.88333

-92.3419444
-100.03333
-91.49333
-91.44944
-100.42
-105.014
-105.034
-104.922
-103.6333333
-103.3418027
-104.98027
-104.75
-101.03
-100.43
-93.1247223
-95.01555
-90.72
-103.228773
-96.941834
-96.628601
-96.866632
-100.92
-99.4247223
-100.46
-99.1388889
-98
-97.167
-96.794
-96.717
-97.25
-98.9
-101.6
-99.67
-99.753778
-102.833
-100.847
-99.342
-97.787
-100.995
-102.65
-105.16
-104.76
-105.78333
-98.8294445
-98.8763889

-100.1168334
-104.07
-104.1
-102.391
-103.767
-103.392
-99.23
-104.203
-99.884
-96.317
-99.177
-92.4666667
-96.61
-100.723
-98.68
-99.502
-99.152
-95.865246
-97.85
-96.601009
-99.078502
-100.042
-98.962
-104.822
-96.505
-94.63
-103.767
-97
-104.6
-104.65
-100.148
-90.2166667
-89.73
-89.9
-87.71
-88.48
-88.618
-88.921
-99.639
-99.639
-103.075639
-99.315166
-99.2516667
-99.9905555
-99.0186111

-103.01
-102.58
-98.7772223
-99.6552778
-100.36
-98.8180556
-100.1180556
-101.3791667
-100.62
-104.33333
-99.88
-99.91
-97.82
-108.594
-96.522668
-96.76472
-99.1513889
-99.1980555
-99.1833333
-99.9
-98.83
-101.3313889
-97.68
-100.61
-97.05
-96.2487
-103.6833333
-104.3
-104.91
-104.95722
-103.768
-103.293
-101.593
-104.018
-103.2
-104.98
-101.143
-108.9166667
-98.9088889
-99.23
-99.733333
-97.458
-99.947
-99.095
-97.805

-99.117
-98.76
-99.1
-97.75
-97.793
-103.76
-104.082
-104.563
-93.0897222
-93.0911111
-99.45
-96.6861111
-97.85
-99.65
-100.033
-96.95
-99.965
-99.537
-101.461
-98.984
-96.95
-94.885
-97.674
-92.08
-104.46666
-104.46666
-109.2727778
-104.517
-104.833
-107.563
-106.526
-108.712
-104.43
-109.34
-109.903
-109.367
-99.9602778
-101.19
-102.35
-99.733333
-96.616667
-104.23
-104.245
-101.713
-101.817

-100.748
-102.332
-104.933
-91.97
-93.45
-100.15
-93.463
-101.46
-101.283
-101.605
-97.061
-96.925
-98.227
-92.63
-104.99
-105.26
-99.17
-96.51
-99.98
-98.67
-100.03
-96.407179
-97.719
-97.563
-99.517
-100.15
-99.94
-103.008
-103.018
-97.08998
-101.3583333
-97.19833
-96.36
-97.576
-101.428
-96.712
-96.284
-96.363
-97
-97.881
-101.148
-101.395
-101.515
-101.579
-97.267

-97.35
-96.667
-97.4028
-97.31562
-107.638
-107.615
-107.128
-104.139
-104.238
-104.143
-99.1202778
-98.48
-103.35
-100.03305
-100.45
-99.3483333
-100.1140556
-103.22
-101.3583333
-97.87027
-105.45
-99.156
-99.238
-99.002
-99.383
-90.167
-88.395
-95.166
-96.733
-103.775221
-101.223
-103.346
-101.152
-104.353
-104.408
-103.346
-104.353
-104.407
-103.358
-101.218
-104.877
-101.737
-101.537
-103.502
-101.894

-102.043
-102.083
-102.001
-99.028
-102.728
-102.024
-101.935
-102.001
-102.576
-104.776
-103.5
-100.955
-104.99
-104.76666
-105
-99.5
-97.111378
-97.064
-104.638
-103.617
-104.976
-103.897
-104.101
-103.936
-103.823
-103.626
-104.007
-102.573
-104.633
-102.847
-104.633
-102.079
-99.233
-104.903
-104.833
-104.33333
-103.5246945
-104.31
-104.089
-104.362
-104.093
-103.809
-103.852
-104.976
-103.524

-103.723
-104.333
-103.183
-103.918
-103.626
-104.85
-104.78333
-104.61666
-104.001
-105.029
-101.22
-103.98
-104.783
-104.6
-102.718
-104.895
-99.5
-103.3391666
-102
-106.99
-108.3333
-105.3
-106.75
-105
-104.6666667
-104.78
-105.53333
-100.8
-99.1436111
-100.28305
-98.8480555
-96.32
-100.25
-103.79
-108.888
-101.9
-96.003
-110.781
-106.835
-103.767
-96.003
-102.633
-108.723
-99.256
-109.108

-104.87
-100.2
-108.008
-104.75
-104.975
-99.268
-100.067
-104.9
-98.95
-108.689
-104.15
-104.333
-101.73
-104.24
-96.32
-98.717
-104.77
-104.24
-98.902
-103.046
-108
-108.33
-99.23
-102
-92.4
-91.83
-91.75
-89.453
-95.04
-94.383
-94.6
-88.57
