# Supplementary material for: Influence of current climate, historical climate stability and topography on species richness and endemism in Mesoamerican geophyte plants
Source: PeerJ. 2017 Oct 20;5:e3932. doi: 10.7717/peerj.3932 (PMC5652257; doi:10.7717/peerj.3932)
Supplement: Table S2 [file peerj-05-3932-s003.pdf]

Table S2. Occurrence data and list of species used for species distribution modeling (SDM).

Species	spatially unique occurrence points
Allium eurotophilum	5
Allium glandulosum	87
Allium haematochiton	8
Alophia drummondii	7
Arisaema macrospathum	12
Beloglottis costaricensis	7
Bessera elegans	15
Bletia adenocarpa	21
Bletia campanulata	55
Bletia coccinea	21
Bletia ensifolia	17
Bletia gracilis	29
Bletia lilacina	8
Bletia macristhmochila	29
Bletia neglecta	26
Bletia parkinsonii	22
Bletia punctata	20
Bletia purpurata	34
Bletia purpurea	108
Bletia roezlii	38
Bletia tenuifolia	6
Brachystele polyantha	8
Calochortus barbatus	18
Calochortus exilis	5
Calochortus fuscus	7
Calochortus purpureus	7
Calochortus spatulatus	8
Calochortus venustulus	6
Cardiostigma longispatha	8
Cipura campanulata	7
Cipura paludosa	15
Corallorhiza bulbosa	5
Crinum americanum	7
Cypripedium irapeanum	11
Cypripedium molle	9
Deiregyne densiflora	8

Dichromanthus cinnabarinus	15
Dichromanthus michuacanus	14
Echeandia echeandioides	9
Echeandia flavescens	11
Echeandia flexuosa	9
Echeandia longipedicellata	5
Echeandia luteola	6
Echeandia mexicana	16
Echeandia nana	7
Echeandia occidentalis	10
Echeandia paniculata	9
Echeandia parviflora	12
Echeandia ramosissima	9
Echeandia reflexa	5
Echeandia scabrella	6
Echeandia skinneri	18
Echeandia vestita	17
Eleutherine bulbosa	5
Eleutherine latifolia	7
Govenia alba	6
Govenia liliacea	14
Govenia mutica	6
Govenia purpusii	7
Govenia superba	12
Habenaria clypeata	20
Habenaria novemfida	9
Habenaria strictissima	5
Habranthus longifolius	5
Hexalectris grandiflora	10
Hymenocallis acutifolia	5
Hymenocallis harrisiana	5
Hymenocallis littoralis	10
Hypoxis colliculata	5
Hypoxis potosina	6
Hypoxys decumbens	26
Hypoxys mexicana	22
Liparis vexillifera	13

Malaxis carnosa	8
Malaxis myurus	11
Maranta gibba	7
Milla biflora	175
Nemastylis tenuis	53
Nothoscordum bivalve	26
Nothoscordum gracile	11
Orthrosanthus exsertus	7
Polianthes geminiflora	11
Polianthes longiflora	10
Polianthes montana	8
Polianthes palustris	13
Polianthes platyphylla	12
Polianthes pringlei	5
Polianthes sessiliflora	7
Polianthes tuberosa	9
Ponthieva mexicana	8
Ponthieva schaffneri	14
Sacoila lanceolata	17
Sarcoglottis assurgens	5
Sarcoglottis schaffneri	14
Schiedeella affinis	6
Schiedeella eriophora	10
Sotoa confusa	5
Sprekelia formosissima	26
Tigridia ehrenbergii	5
Tigridia meleagris	5
Tigridia multiflora	12
Tigridia pavonia	18
Trimezia steyermarkii	5
Triphora gentianoides	5
Weldenia candida	7
Zephyranthes brevipes	6
Zephyranthes carinata	13
Zephyranthes lindleyana	7
Zephyranthes verecunda	6
