# Supplementary material for: Influence of current climate, historical climate stability and topography on species richness and endemism in Mesoamerican geophyte plants
Source: PeerJ. 2017 Oct 20;5:e3932. doi: 10.7717/peerj.3932 (PMC5652257; doi:10.7717/peerj.3932)
Supplement: Table S3 — Values are averages of ten cross-validated iterations. [file peerj-05-3932-s004.pdf]

Table S3. Model performance estimated with the area under the curve (AUC) and true skill statistic (TSS) for all the species that had at least five spatially unique points. Bold values represent species with low model performance and were not included to estimate species richness (SR) and weighted endemism (WE) using species distribution models (SDM).

Species	Training samples	Test samples	Test AUC	TSS
Allium eurotophilum	4	1	0.919	0.73887827
Allium glandulosum	70	17	0.8688	0.63015503
Allium haematochiton	7	1	0.9358	0.86929573
Alophia drummondii	**6**	**1**	**0.8501**	**0.48553219**
Arisaema macrospathum	10	2	0.8778	0.7235341
Beloglottis costaricensis	**6**	**1**	**0.7717**	**0.49104525**
Bessera elegans	12	3	0.9508	0.65844487
Bletia adenocarpa	17	4	0.9561	0.69394519
Bletia campanulata	44	11	0.9114	0.68861753
Bletia coccinea	17	4	0.9202	0.65763225
Bletia ensifolia	14	3	0.8918	0.6027884
Bletia gracilis	24	5	0.9286	0.63003824
Bletia lilacina	7	1	0.9296	0.79901211
Bletia macristhmochila	24	5	0.9074	0.5960325
Bletia neglecta	21	5	0.9552	0.68882728
Bletia parkinsonii	18	4	0.9671	0.83107075
Bletia punctata	16	4	0.9522	0.79819949
Bletia purpurata	**28**	**6**	**0.8744**	**0.47684831**
Bletia purpurea	87	21	0.8979	0.60337567
Bletia roezlii	31	7	0.9361	0.6859146
Bletia tenuifolia	5	1	0.9881	0.68376354
Brachystele polyantha	7	1	0.8808	0.72031549
Calochortus barbatus	**15**	**3**	**0.7895**	**0.46042065**
Calochortus exilis	**4**	**1**	**0.8393**	**0.44609624**
Calochortus fuscus	**6**	**1**	**0.8197**	**0.41402167**
Calochortus purpureus	6	1	0.9398	0.69297323
Calochortus spatulatus	**7**	**1**	**0.7386**	**0.12543021**
Calochortus venustulus	5	1	0.9161	0.66097833
Cardiostigma longispatha	7	1	0.9577	0.68044933
Cipura campanulata	6	1	0.8116	0.54080625
Cipura paludosa	12	3	0.8422	0.55688337
Corallorhiza bulbosa	**4**	**1**	**0.813**	**0.25310707**
Crinum americanum	6	1	0.8354	0.64181007
Cypripedium irapeanum	9	2	0.859	0.54855003
Cypripedium molle	8	1	0.9687	0.86731995
Deiregyne densiflora	7	1	0.8883	0.58741236
Dichromanthus cinnabarinus	**12**	**3**	**0.7711**	**0.34643085**

Dichromanthus michuacanus	12	2	0.8699	0.56454748
Echeandia echeandioides	8	1	0.8564	0.74431166
Echeandia flavescens	**9**	**2**	**0.8127**	**0.41622052**
Echeandia flexuosa	8	1	0.9235	0.6444232
Echeandia longipedicellata	4	1	0.9368	0.53669535
Echeandia luteola	5	1	0.9716	0.56596558
Echeandia mexicana	13	3	0.8743	0.55895475
Echeandia nana	6	1	0.9617	0.65559273
Echeandia occidentalis	**8**	**2**	**0.813**	**0.41833971**
Echeandia paniculata	8	1	0.9364	0.61116953
Echeandia parviflora	10	2	0.8965	0.64369025
Echeandia ramosissima	8	1	0.9001	0.68956342
Echeandia reflexa	4	1	0.9305	0.53161249
Echeandia scabrella	5	1	0.9223	0.51655513
Echeandia skinneri	15	3	0.9059	0.52560548
Echeandia vestita	14	3	0.9503	0.74859783
Eleutherine bulbosa	**4**	**1**	**0.8434**	**0.45927342**
Eleutherine latifolia	6	1	0.8889	0.74512428
Govenia alba	5	1	0.7843	0.54271829
Govenia liliacea	12	2	0.8062	0.54604844
Govenia mutica	**5**	**1**	**0.8477**	**0.40095602**
Govenia purpusii	6	1	0.9159	0.61470682
Govenia superba	**10**	**2**	**0.8436**	**0.47327916**
Habenaria clypeata	16	4	0.9252	0.57573295
Habenaria novemfida	8	1	0.9333	0.68218611
Habenaria strictissima	**4**	**1**	**0.9231**	**0.40305927**
Habranthus longifolius	**4**	**1**	**0.7611**	**0.42866475**
Hexalectris grandiflora	**8**	**2**	**0.7414**	**0.38237731**
Hymenocallis acutifolia	**4**	**1**	**0.8346**	**0.49840663**
Hymenocallis harrisiana	4	1	0.8605	0.55928936
Hymenocallis littoralis	8	2	0.8602	0.52173359
Hypoxis colliculata	**4**	**1**	**0.969**	**0.46741555**
Hypoxis potosina	5	1	0.8851	0.63223391
Hypoxys decumbens	21	5	0.921	0.66775653
Hypoxys mexicana	18	4	0.8521	0.56217336
Liparis vexillifera	**11**	**2**	**0.8066**	**0.40489165**
Malaxis carnosa	7	1	0.8741	0.63996176
Malaxis myurus	**9**	**2**	**0.7915**	**0.49869344**
Maranta gibba	6	1	0.9412	0.72294455
Milla biflora	140	35	0.8967	0.6412014
Nemastylis tenuis	43	10	0.8467	0.54286807
Nothoscordum bivalve	21	5	0.8797	0.53514659
Nothoscordum gracile	**9**	**2**	**0.7233**	**0.34665392**

Orthrosanthus exssertus	**6**	**1**	**0.8398**	**0.47761313**
Polianthes geminiflora	9	2	0.9001	0.53832059
Polianthes longiflora	8	2	0.9725	0.85521033
Polianthes montana	7	1	0.9394	0.60462078
Polianthes palustris	11	2	0.9273	0.75506692
Polianthes platyphylla	10	2	0.9483	0.79899618
Polianthes pringlei	4	1	0.9342	0.71641173
Polianthes sessiliflora	**6**	**1**	**0.94**	**0.46061185**
Polianthes tuberosa	8	1	0.8591	0.77833015
Ponthieva mexicana	7	1	0.9264	0.58659975
Ponthieva schaffneri	12	2	0.9048	0.71139261
Sacoila lanceolata	**14**	**3**	**0.7592**	**0.43840025**
Sarcoglottis assurgens	**4**	**1**	**0.7543**	**0.32633843**
Sarcoglottis schaffneri	12	2	0.89	0.56661887
Schiedeella affinis	**5**	**1**	**0.8043**	**0.40736138**
Schiedeella eriophora	8	2	0.8944	0.55833333
Sotoa confusa	4	1	0.8945	0.63768324
Sprekelia formosissima	21	5	0.9012	0.66059273
Tigridia ehrenbergii	**4**	**1**	**0.8495**	**0.47987572**
Tigridia meleagris	4	1	0.9383	0.71121734
Tigridia multiflora	10	2	0.8467	0.5900733
Tigridia pavonia	**15**	**3**	**0.8618**	**0.44687699**
Trimezia steyermarkii	4	1	0.9501	0.81537604
Triphora gentianoides	4	1	0.8816	0.53677502
Weldenia candida	6	1	0.9788	0.84788082
Zephyranthes brevipes	5	1	0.903	0.52530274
Zephyranthes carinata	11	2	0.9156	0.6805768
Zephyranthes lindleyana	**6**	**1**	**0.6576**	**0.40025494**
Zephyranthes verecunda	5	1	0.9663	0.83217017
